# An Integrated Model of Minor Intron Emergence and Conservation

**DOI:** 10.3389/fgene.2019.01113

**Published:** 2019-11-13

**Authors:** Marybeth Baumgartner, Kyle Drake, Rahul N. Kanadia

**Affiliations:** ^1^Department of Physiology and Neurobiology, University of Connecticut, Mansfield, CT, United States; ^2^Institute of Brain and Cognitive Sciences, University of Connecticut, Mansfield, CT, United States; ^3^Institute of Systems Genomics, University of Connecticut, Mansfield, CT, United States

**Keywords:** minor introns, minor spliceosome, eukaryotic evolution, essential genes, scaling, multicellularity, animal domestication, human disease

## Abstract

Minor introns constitute <0.5% of the introns in the human genome and have remained an enigma since their discovery. These introns are removed by a distinct splicing complex, the minor spliceosome. Both are ancient, tracing back to the last eukaryotic common ancestor (LECA), which is reflected by minor intron enrichment in specific gene families, such as the mitogen activated-protein kinase kinases, voltage-gated sodium and calcium ion channels, and E2F transcription factors. Most minor introns occur as single introns in genes with predominantly major introns. Due to this organization, minor intron-containing gene (MIG) expression requires the coordinated action of two spliceosomes, which increases the probability of missplicing. Thus, one would expect loss of minor introns *via* purifying selection. This has resulted in complete minor intron loss in at least nine eukaryotic lineages. However, minor introns are highly conserved in land plants and metazoans, where their importance is underscored by embryonic lethality when the minor spliceosome is inactivated. Conditional inactivation of the minor spliceosome has shown that rapidly dividing progenitor cells are highly sensitive to minor spliceosome loss. Indeed, we found that MIGs were significantly enriched in a screen for genes essential for survival in 341 cycling cell lines. Here, we propose that minor introns inserted randomly into genes in LECA or earlier and were subsequently conserved in genes crucial for cycling cell survival. We hypothesize that the essentiality of MIGs allowed minor introns to endure through the unicellularity of early eukaryotic evolution. Moreover, we identified 59 MIGs that emerged after LECA, and that many of these are essential for cycling cell survival, reinforcing our essentiality model for MIG conservation. This suggests that minor intron emergence is dynamic across eukaryotic evolution, and that minor introns should not be viewed as molecular fossils. We also posit that minor intron splicing was co-opted in multicellular evolution as a regulatory switch for *en masse* control of MIG expression and the biological processes they regulate. Specifically, this mode of regulation could control cell proliferation and thus body size, an idea supported by domestication syndrome, wherein MIGs are enriched in common candidate animal domestication genes.

## Introduction

For most eukaryotic protein-coding genes to be expressed, non-coding intronic sequences must be removed from their pre-mRNA transcripts and their coding exons ligated together. In many eukaryotes, there are two types of introns: major introns, which comprise the vast majority (> 99.5%) of introns, and minor introns (< 0.5% of introns) (Levine and Durbin, 2001; Patel and Steitz, 2003; Alioto, 2007). The difference in consensus sequences of the major and minor introns requires that two different splicing machineries recognize and excise these introns (Patel and Steitz, 2003). These splicing machineries, known as spliceosomes, are ribonucleoprotein complexes containing five small nuclear RNAs (snRNAs) and a collection of associated proteins (Turunen et al., 2013). Major introns can only be removed by the major spliceosome, which contains the snRNAs U1, U2, U4, U5, and U6 (Patel and Steitz, 2003). Minor introns are excised by the minor spliceosome, which consists of the unique snRNAs U11, U12, U4atac, and U6atac, along with the shared U5 snRNA (Tarn and Steitz, 1996a; Tarn and Steitz, 1996b). Both major introns and minor introns, along with their respective spliceosomes, can be traced back to the last eukaryotic common ancestor (LECA) or earlier (Russell et al., 2006).

Most introns in minor intron-containing genes (MIGs) are major introns, with only one or two minor introns (Sheth et al., 2006; Alioto, 2007; Olthof et al., 2019). Therefore, expression of MIGs not only requires the expression, formation, and recruitment of both spliceosomes, but it also necessitates spatiotemporal coordination of major spliceosome and minor spliceosome activity along the pre-mRNA transcript (Gornemann et al., 2005; Listerman et al., 2006). The expression and formation of two separate spliceosomes increases the metabolic load on the cell, because both spliceosomes use the same transcription and processing pathways. For example, the expression of all spliceosomal snRNAs requires RNA polymerase II and RNA polymerase III activity (Kiss, 2004; Patel and Bellini, 2008). Furthermore, these nascent snRNAs must then be processed into their mature forms by shared pathways (Kiss, 2004; Pessa et al., 2008). The formation of the minor spliceosome also requires the U5 snRNA and >30 proteins shared between the minor and major spliceosomes (Tarn and Steitz, 1996b; Will et al., 1999; Will et al., 2004). Therefore, the expression/assembly of the minor spliceosome necessitates increased production of numerous nuclear, nuclear envelope-bound, and cytoplasmic proteins to maintain normal rates of gene expression and major spliceosome assembly. Based on the complex spliceosomal coordination required for MIG expression and the high metabolic load linked to the maintenance of the minor spliceosome, one would anticipate strong purifying selection against minor introns and the minor spliceosome. Specifically, since i) minor introns and the minor spliceosome emerged in early, unicellular eukaryotes, ii) unicellular species generally have high effective population sizes, and iii) effective population size is positively correlated with the strength of purifying selection, one would expect that minor introns would have been completely lost in early eukaryotic evolution (Lynch, 2002; Lynch and Conery, 2003). While nine independent events of complete loss of minor introns/the minor spliceosome have been identified throughout eukaryotic evolution, minor introns and the minor spliceosome are observed in many modern genomes, indicating that they endured in multiple lineages (Davila Lopez et al., 2008; Bartschat and Samuelsson, 2010).

In particular, minor introns and the minor spliceosome are highly conserved in metazoans and land plants (Russell et al., 2006; Szczesniak et al., 2013). In fact, in land plants and animals, the positions of minor introns are more highly conserved than the positions of major introns (Basu et al., 2008a). This high degree of conservation in these specific species suggests two possibilities: i) minor intron splicing conferred an advantage unique to these lineages despite the costs associated with maintaining two parallel splicing machineries, or ii) in these lineages, the cost of losing minor introns and the minor spliceosome was higher than the cost of maintaining two splicing machineries. In these species, complete inactivation of the minor spliceosome causes early embryonic lethality, indicating that minor introns are fixed in genes essential for early development, when cells are rapidly dividing (Otake et al., 2002; Kim et al., 2010; Jung and Kang, 2014; Xu et al., 2016; Baumgartner et al., 2018; Doggett et al., 2018). In the past year, research using models of conditional inactivation of the minor spliceosome has further underscored the importance of minor splicing for the survival of rapidly dividing progenitor pools, whereas differentiated cells are less affected by minor spliceosome inactivation (Baumgartner et al., 2018; Doggett et al., 2018).

In order to understand the dichotomy of complete minor intron loss *versus* high minor intron conservation across eukaryotic evolution, we sought to unify the insights from these developmental studies with past models of minor intron emergence and evolution. Here, we 1) propose a revised model describing the emergence and conservation of minor introns and the minor spliceosome; 2) postulate how MIGs are linked to each other; and ultimately 3) posit that minor splicing may have been a powerful target to regulate body size in multicellular organism evolution, such as in animal domestication.

### The Emergence of Minor Intron Splicing

**Figure 1:**
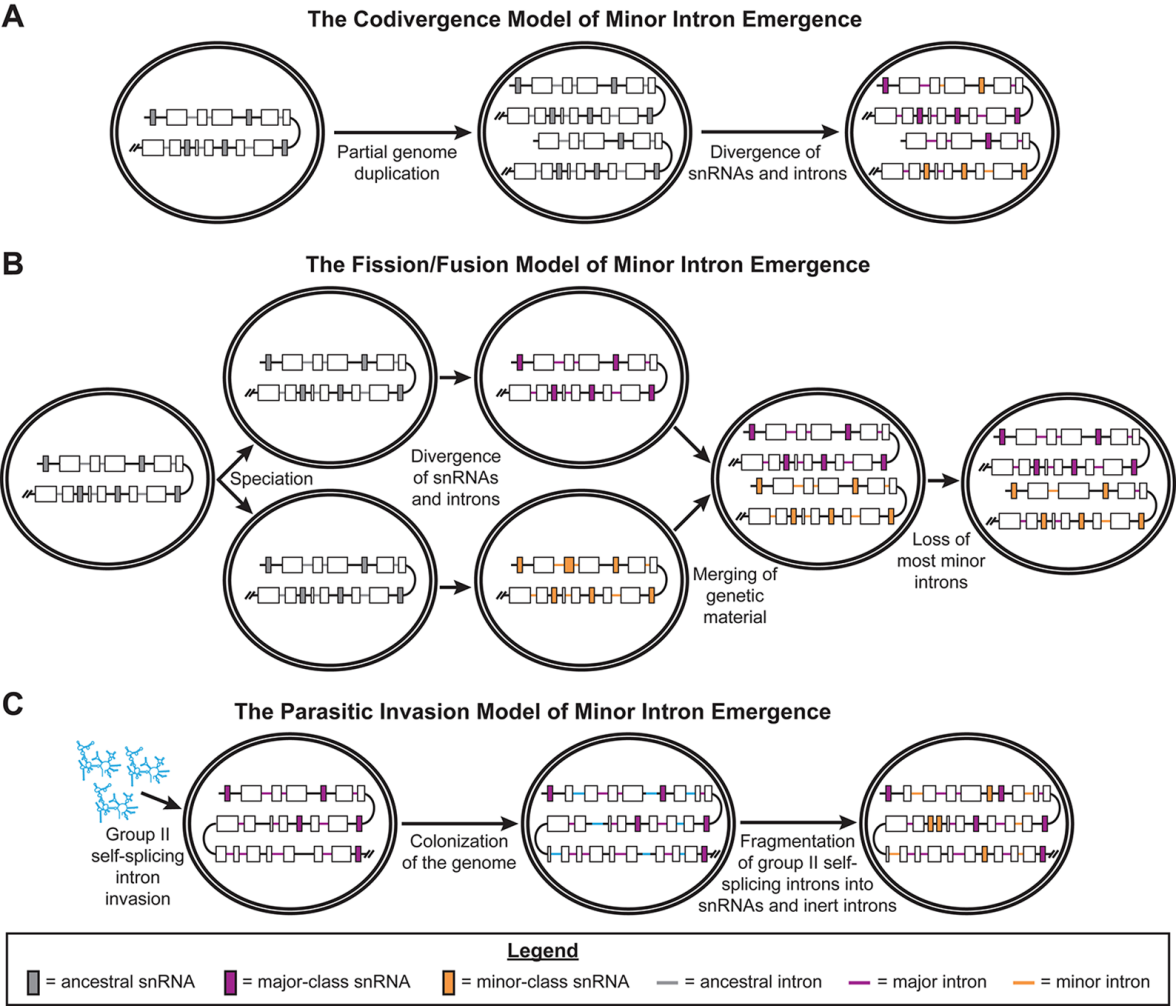
Previously published models of minor intron emergence. Schematics showing the three models of minor intron emergence proposed by Burge et al. (1998), including **(A)** the codivergence model, **(B)** the fission/fusion model, and **(C)** the parasitic invasion model. Unicellular organisms are shown by the double-bordered ovals, while regions of the genome are displayed as lines. Black lines denote intergenic regions, gray lines for ancestral introns, blue lines for major introns, and purple lines for minor introns. Boxes represent coding regions of genes, with colored boxes denoting ancestral snRNA genes (gray), major spliceosomal snRNA genes (blue), or minor spliceosome-specific snRNA genes (purple). Arrows denote the passage of time; events occurring during this period of time are noted in text under the respective arrow. Reproduced with permission from Burge et al., 1998.

In 1998, the Sharp group proposed three different models to explain the emergence of minor introns and the minor spliceosome. Two of these models posit that all spliceosome-dependent introns (“spliceosomal introns”) and spliceosomal snRNAs share common ancestors, and that a period of divergence produced the separate splicing pathways we observe today. Specifically, in the codivergence model, duplication of the ancestral spliceosomal snRNA genes in the early eukaryotic genome triggered the divergence of two distinct spliceosomes, which then drove the emergence of the two types of introns (**Figure 1A**) (Burge et al., 1998). In the fission/fusion model, the trigger for this divergence was a speciation event, producing two lineages with one type of spliceosome and one type of intron each (“fission”). After substantial divergence of these introns and spliceosomal components into the major- and minor-types, the genetic material from these two lineages merged back together (“fusion”) (**Figure 1B**) (Burge et al., 1998). Specifically, Burge et al. proposed this may have occurred *via* endosymbiosis. To explain the presence of components that function in both the major and minor spliceosomes, such as the U5 snRNA and numerous protein components, this model postulates that either i) some components of the ancestral spliceosome diverged less than others, allowing these components to function in both of the derived spliceosomes; or ii) these spliceosomal components evolved after the “fusion” event (Burge et al., 1998).

The third model described by the Sharp group is the parasitic invasion model. This model is very similar to the endosymbiont model, which describes the origin of spliceosomal introns in eukaryotes (Cavalier-Smith, 1991; Sharp, 1991; Koonin, 2006). In both of these models, a wave of group II, self-splicing introns invaded the early eukaryotic genome, prior to the evolution of the nuclear envelope (Martin and Koonin, 2006). Group II, self-splicing introns are retroelements that, when transcribed into RNA, autocatalyze their removal from RNA transcripts. The two-step *trans*-esterification splicing reaction used by this class of self-splicing introns is very similar to the splicing reactions driven by both the major and minor spliceosomes (Lambowitz and Zimmerly, 2011). Group II introns also express an intron-encoded protein (IEP), which contains endonuclease and reverse-transcriptase domains, that allows these introns to “reverse splice” themselves into the genome (Lambowitz and Zimmerly, 2011). Both the endosymbiont and parasitic invasion models invoke the fragmentation of these group II, self-splicing introns into catalytic subunits, which evolved into the spliceosomal snRNA genes, and inert sequences recognized by these catalytic subunits, which evolved into spliceosomal introns (Sharp, 1991; Martin and Koonin, 2006; Lambowitz and Zimmerly, 2011). In the parasitic invasion model proposed by the Sharp group, there were two temporally distinct waves of group II, self-splicing intron invasion in the early eukaryotic lineage. The first wave of invading introns fragmented to produce the major spliceosomal snRNAs and major introns. In this early eukaryotic genome with major introns, a second wave of group II introns invaded and then fragmented to produce the minor spliceosome-specific snRNAs and minor introns (**Figure 1C**) (Burge et al., 1998).

Both the codivergence and fission/fusion models predict that major and minor introns are approximately the same evolutionary age, since they arose from the same ancestral introns. The fission/fusion model also predicts that the initial evolution of the major and minor spliceosomes occurred separately. In contrast, the parasitic invasion model does not presuppose either of these events. The former prediction is partially supported by a genomic scan for minor introns and minor spliceosome components, which were identified in genomes from all five eukaryotic supergroups. Thus, minor introns and the minor spliceosome are likely ancient, tracing back to LECA or earlier (Russell et al., 2006). However, two groups have published findings that contradict these predictions of the codivergence and fission/fusion models. First, the Koonin group employed a bioinformatics-based strategy to determine whether ancient introns were major introns or minor introns (Basu et al., 2008b). From their results, they concluded that major introns likely emerged before minor introns in early eukaryotic evolution, supporting the parasitic invasion model of the emergence of minor introns (Basu et al., 2008b). Second, the Luhrmann group found that most, if not all, of the proteins in the U4/U6.U5 tri-small nuclear ribonucleoprotein (snRNP) are shared with the U4atac/U6atac.U5 tri-snRNP of the minor spliceosome (Will et al., 1999; Nottrott et al., 2002). From this observation, they argued that the minor spliceosome evolved alongside the major spliceosome, in contradiction to the prediction of the fission/fusion model. Like the Koonin group, the Luhrmann group argued in favor of the parasitic invasion model for the emergence of minor introns (Will et al., 1999).

### Conservation of Minor Intron Splicing

While Russell et al. found minor introns in at least one genome from each of the five eukaryotic supergroups, minor splicing components and minor introns could not be identified in numerous species within these supergroups (Russell et al., 2006). In fact, the Samuelsson group found that minor splicing had been independently lost at least nine times across eukaryotic evolution, with two loss events in *Amoebozoa*, in *Dictyostelium* and *Entamoebidae*; one loss event in the diatom lineage; two loss events affecting the green and *Cyanidiophyceae* algal lineages in *Archaeplastida*; one loss event in *Monosiga*; two loss events in fungi, affecting the *Microsporidia* and *Dikarya* clades; and one loss event in metazoa, in the *Caenorhabditis* branch of nematodes (Bartschat and Samuelsson, 2010). Even in lineages that have retained minor introns and the minor spliceosome, the Makałowski group found higher rates of minor intron loss in dipteran insects than in other *Insecta* lineages (Janice et al., 2012). Oddly, the dipteral lineage also contains the only known example of *de novo* minor intron gain in a species—i.e., the emergence of a non-ancestral minor intron. This gene, *Zrsr2*, has been shown to be essential for minor splicing, and the addition of a minor intron in this gene may represent selection toward auto-regulation within the minor splicing pathway (Lin et al., 2010; Madan et al., 2015). Together, these findings underscore the dynamic flux in the maintenance of the minor spliceosome and minor introns across eukaryotic evolution, tracing back to early eukaryotes.

Despite the preponderance of evidence indicating selection against minor splicing throughout eukaryotic evolution, minor introns and the minor spliceosome are highly conserved in most metazoans and plants. In fact, when the Koonin group analyzed the positions of major and minor introns in orthologous human and *Arabidopsis thaliana* genes, they found that the positions of minor introns within a gene are more highly conserved between plants and animals than those of major introns (Basu et al., 2008a). This contradiction is unexpected—why is this pathway so highly conserved among metazoans and plants, when it has been lost multiple times in other eukaryotic lineages? One possibility, proposed by Mount in 1996, is that minor splicing is a molecular fossil (Mount, 1996). In this model, the very presence of inert minor introns in the genome required the conservation of the minor splicing machinery, and the maintenance of these minor introns would be informed by the rates of intron loss in a given species (Mount, 1996). Thus, one would expect that higher overall rates of intron loss in a species would correspond to higher rates of minor intron loss. Indeed, in *Animalia*, lower rates of minor intron loss are observed in vertebrates than invertebrates, mirroring rates of overall intron loss in these lineages (Lin et al., 2010). For example, *Caenorhabditis elegans* has a high rate of intron loss, and this species has completely lost its minor introns and the minor spliceosome (Carmel et al., 2007; Davila Lopez et al., 2008; Bartschat and Samuelsson, 2010; Lin et al., 2010). Minor intron loss can occur *via* two distinct mechanisms. In the first, a processed mRNA undergoes reverse transcription to produce intronless cDNA, followed by homologous recombination of this cDNA with the genome, resulting in intron loss and exon fusion (Burge et al., 1998; Coulombe-Huntington and Majewski, 2007). Loss of entire introns is correlated with gene expression, such that higher expression of a gene increases the availability of its processed mRNA and thus the likelihood of recombination-driven intron loss (Coulombe-Huntington and Majewski, 2007). Second, minor intron loss can occur through conversion of a minor intron into a major intron, driven by mutation of the minor-class consensus sequences (Dietrich et al., 1997; Burge et al., 1998). This requires sequential single nucleotide polymorphisms in the minor-class consensus sequences, which would produce intermediate, weak consensus sequences. Consequently, neither the major nor minor spliceosomes could recognize this intron, impairing its splicing and gene expression. Therefore, for minor-to-major intron conversion, one would expect higher rates of minor intron loss in genes with low expression and/or non-essential functions. Based on these observations and the molecular fossil model, one would predict that the conservation of minor introns and the minor spliceosome is dependent on the rates of intron loss in a given species, the expression and function of MIGs, and the method of minor intron loss.

Another possibility is that minor intron splicing evolved additional regulatory roles, separate from its requirement to remove ancestral minor introns from crucial genes. This concept was first proposed by the Steitz group, when they observed inefficient minor intron splicing in HeLa and S2 *Drosophila melanogaster* cell lines, which they linked to low levels of the minor-class snRNAs (Patel et al., 2002). These unspliced transcripts would either be trapped in the nucleus, be degraded in the nucleus by the exosome, or be degraded in the cytoplasm *via* nonsense-mediated decay (Patel et al., 2002; Hirose et al., 2004; Niemela et al., 2014). In this model, minor intron splicing is a regulatory switch that allows the cell to regulate the expression of MIGs *en masse*, by altering the levels of the minor spliceosome components and thereby controlling the rate of minor intron splicing. For this manuscript, we will refer to this as the regulatory switch model. The regulatory switch model is supported by work from the Dreyfuss group, who found that U6atac was the rate-limiting component of the minor spliceosome (Younis et al., 2013). When they stabilized U6atac snRNA in HeLa cells, thus increasing U6atac levels, they observed increased rates of minor intron splicing both in a transfected splicing construct and across multiple endogenous genes (Younis et al., 2013).

Additionally, the minor spliceosome and minor introns may play a regulatory role in alternative splicing. In *Drosophila*, the MIG *prospero*, which encodes a neuronal transcription factor, has a major intron nested within a minor intron; this nested intron organization was called a twintron. Minor intron splicing of this twintron would remove 29 amino acids, 5 of which encode the N-terminal homeodomain, but would still maintain the codon frame (Otake et al., 2002). In 2004, Scamborova et al. showed that the nested major intron is preferentially spliced during early embryogenesis, while the outer minor intron is preferentially spliced in later embryonic development (Scamborova et al., 2004). Genes with similar twintron architectures have been identified in numerous insect and vertebrate genomes, indicating that this type of isoform regulation may be relatively common (Mount et al., 2007; Janice et al., 2013). Other MIGs have staggered major- and minor-type consensus sequences, such that the decision to recruit the major *vs.* the minor spliceosome would result in mutually exclusive exon selection (Letunic et al., 2002; Chang et al., 2007; Janice et al., 2013). Moreover, the U11 snRNA itself has also been shown to regulate alternative splicing by base-pairing to intronic U11 snRNP-binding splicing enhancers (USSEs), which contain tandem repeats of minor-type 5’ splice site-like sequences (Verbeeren et al., 2010). While USSEs have only been identified in *SNRNP48* and *RNPC3* thus far, it is notable that these genes encode minor spliceosome-specific proteins, indicating the presence of an auto-regulatory circuit in the minor splicing pathway (Verbeeren et al., 2010). Finally, cross-talk between the major and minor spliceosomes has been suggested by Madan et al., based on their RNAseq analysis of myelodysplastic syndrome (MDS) patients with *ZRSR2* mutations (Madan et al., 2015). Moreover, the Kanadia group recently identified numerous events of alternative splicing across minor introns that are executed by the major spliceosome, which are regulated in a tissue-specific manner in both mouse and human (Olthof et al., 2019). Together, these findings indicate that alternative splicing decisions mediated by the major spliceosome may be informed by either minor introns or the minor spliceosome itself, although the underlying mechanism remains unknown.

### The Functions of Minor Intron-Containing Genes

Ever since MIGs were first identified, investigators have sought to understand why these specific genes have such highly conserved minor introns. Both models of minor splicing conservation predict that MIGs shared specific traits, such as specific sets of functions or expression patterns, that informed the conservation of their minor introns. For example, the regulatory switch model predicts that MIGs will share a small set of functions, allowing tight control of a specific subset of biological processes. To discuss MIG functions, we have divided the following section based on the three general approaches that have been employed in MIG function research: 1) identification of minor intron-enriched gene families; 2) functional classification of MIGs; and 3) identification of the biological processes disrupted by inactivation of the minor spliceosome.

#### Identification of Minor Intron Enrichment in Gene Families

Since MIGs trace back to LECA, any gene family produced by duplication of an ancestral MIG would be expected to be minor intron-rich. Indeed, minor intron enrichment is observed in numerous gene families, which can be found in the minor intron database (MIDB; midb.pnb.uconn.edu) (Olthof et al., 2019). The enrichment of MIGs in specific gene families was the first indication that minor splicing might control specific functions. For example, in 1999, Wu and Krainer noted the presence of multiple MIGs within the voltage-gated sodium channel (VGSC) and voltage-gated calcium channel (VGCC) α subunit gene families, which encode the pore region of these voltage-gated ion channels (Wu and Krainer, 1999; Catterall et al., 2005; Buraei and Yang, 2010). Based on the updated list of human minor introns in the MIDB, 100% of VGSC and VGCC α subunit genes contain at least one minor intron (Catterall et al., 2005; Buraei and Yang, 2010; Olthof et al., 2019). Moreover, all four VGCC auxiliary α2δ subunit genes, which function in VGCC trafficking and gating modulation, are MIGs (Davies et al., 2007; Olthof et al., 2019). Together, these observations strongly suggest that cell excitability, particularly action potential propagation and muscle contraction, and vesicle fusion are regulated by minor splicing (Catterall et al., 2005; Davies et al., 2007; Buraei and Yang, 2010).

Numerous other MIG-rich gene families have been identified, spanning a wide variety of functions. For example, minor introns are found in all four matrilin genes, implicating minor splicing in the regulation of extracellular matrix assembly, particularly in cartilage (Muratoglu et al., 2000; Klatt et al., 2011). Enrichment of MIGs is also observed in the E2F transcription factor (75% of genes), phospholipase C (60% of genes), diaphanous-related formin (100% of genes), GPN-loop GTPase (100% of genes), mitogen activated-protein kinase (MAPK; 69% of genes), phosphatase 2 (PP2a) regulatory B55 subunit (100% of genes), and a subset of guanine nucleotide exchange factor (GEF) gene families, including 100% of Ras guanyl-releasing (RASGRP) genes and 71% of Rap-GEF genes (Levine and Durbin, 2001; NCBI, 2018; Olthof et al., 2019). Notably, the RASGRP and MAPK gene families converge on the Ras signaling pathway, which modulates cell survival, growth, and differentiation (Cargnello and Roux, 2011). Moreover, the E2F transcription factors are well-known regulators of cell proliferation and differentiation, while the B55 subunit of PP2A conveys substrate specificity, allowing for the dephosphorylation of retinoblastoma family and Smad proteins, in turn inhibiting E2F activity (Dimova and Dyson, 2005; Kurimchak and Grana, 2015). Given the high percentage of MIGs found in all of these gene families, it is plausible that minor splicing also plays a role in regulating cell proliferation and differentiation. Indeed, as detailed in the following *Biological Processes Affected by Minor Spliceosome Inhibition* subsection, disruptions in minor splicing have been associated with abnormal cell cycle regulation and differentiation (Madan et al., 2015; Gault et al., 2017; Baumgartner et al., 2018; Doggett et al., 2018).

#### Functional Classification of Minor Intron-Containing Genes

The regulatory switch model predicts that all MIGs are linked by a set of shared functions. While gene family analysis provides insight into some functions highly sensitive to minor splicing, it cannot assess functional relationships among all MIGs. Attempts to classify all MIGs into specific functional roles occurred simultaneously with the gene family-based analyses described in the previous subsection. The first functional category proposed was “information processing,” when Burge et al. reported that many (25%) of the 56 MIGs they identified in their multi-genome scan were involved in functions such as DNA replication, DNA repair, transcription, RNA processing, and translation (Burge et al., 1998). Later efforts continued to identify the enrichment of MIGs in similar “information processing” functions, such as RNA metabolism pathways, spanning both mRNA and non-coding RNA processing; DNA replication/repair; and transcription regulation (Chang et al., 2007; Merico et al., 2015). By leveraging mouse phenotypes from the Mammalian Phenotype Ontology dataset and functional gene-set resources, Merico et al. found that the 744 MIGs they identified in the human genome were also enriched for ion channels, cell cycle, neurodevelopment, skeletal development, and embryonic survival (Merico et al., 2015). In plants, MIG functions have been similarly investigated, using Pfam domain analysis to study MIGs conserved between maize (*Zea mays*) and human (Gault et al., 2017). Their initial analysis of 408 maize MIGs revealed functional enrichments in cell cycle, RNA processing, transcription, translation, protein folding/degradation, and metabolism, similar to the functions identified in previous reports of human MIGs (Gault et al., 2017).

While identifying the function of a MIG in isolation is the first step toward understanding the processes controlled by MIGs, the *in vivo* role of MIGs can only be analyzed in the light of MIG expression. For example, we have previously found dynamic shifts in MIG expression across mouse retinal development: MIGs that were highly expressed in the embryonic retina predominantly perform RNA processing, ribosome biogenesis, and cell cycle functions, while MIGs enriched in the postnatal retina control vesicle-mediated transport and VGCC activity (Baumgartner et al., 2015). However, even these functions were disparate; the largest functional category we identified, cell cycle, was supported by only 12% of the embryonically enriched MIGs (Baumgartner et al., 2015). Thus, MIGs that share a function, but are not expressed simultaneously, will have different consequences when disrupted. In all, minor introns are found in disparate genes and gene families that do not overlap in a neat set of molecular/biological pathways.

#### Biological Processes Affected by Minor Spliceosome Inhibition

In the past decade, minor spliceosome inactivation in different model systems has been used to identify the functions that require normal MIG expression. The most common phenotype associated with complete minor spliceosome inactivation is early lethality. In *D. melanogaster*, P element-mediated disruption of the U12 genes resulted in embryonic lethality (Otake et al., 2002). In numerous reports, the Kang group has shown that T-DNA-mediated disruption of distinct minor spliceosome-specific protein genes, including the *U11/U12-31K*, *U11/U12-48K*, and *U11/U12-65K* genes, all result in embryonic lethality in *A. thaliana* (Kim et al., 2010; Jung and Kang, 2014; Xu et al., 2016). In mice, constitutive deletion of the *Rnu11* gene, which encodes the U11 snRNA, or the gene *Rnpc3*, which encodes the U11/U12-65K protein, results in early embryonic lethality (Baumgartner et al., 2018; Doggett et al., 2018). In all of these experiments, early embryonic lethality has prevented researchers from pinpointing the biological pathways regulated by the minor spliceosome.

Many investigators have therefore employed knockdown of minor spliceosome components to understand the biological pathways controlled by minor splicing. In *Drosophila*, P element-mediated disruption of the U6atac gene results in third instar death instead of embryonic lethality, due to maternally contributed wild-type U6atac snRNA (Otake et al., 2002). Analysis of the transcriptional changes in these larvae revealed significant downregulation of general metabolism genes in the mutant larvae (Pessa et al., 2010). In *A. thaliana*, artificial microRNA (amiRNA)-mediated knockdown of distinct minor spliceosome-specific protein genes results in a consistent set of phenotypes, characterized by leaf serration, arrested meristem growth, and post-bolting rosette leaf production (Kim et al., 2010; Jung and Kang, 2014; Xu et al., 2016). Notably, external application of the hormone gibberellic acid, which induces cell division and expansion, could rescue the inflorescence stem growth in the transgenic lines; qRT-PCR analysis revealed downregulation of gibberellic acid metabolism genes in the amiRNA-treated plants (Kim et al., 2010; Xu et al., 2016). In zebrafish (*Danio rerio*), the Heath group found that mutation in an intron of *rnpc3*, is the causative mutation for the *clbn* mutant line (Markmiller et al., 2014). The *clbn* mutants display microcephaly, microphthalmia, swim bladder defects, pancreas and liver hypoplasia, intestinal epithelium abnormalities, and death between 6.5 to 9.5 days post-fertilization (Markmiller et al., 2014). RNAseq analysis revealed that MIGs involved in mRNA quality control and cell cycle repression were differentially expressed or displayed some degree of minor intron retention in the mutant larvae (Markmiller et al., 2014). This finding led them to propose a model in which disruption of these mRNA control pathway MIGs leads to the destabilization of overall gene expression, minor and major intron-containing alike, by affecting multiple levels of mRNA processing, including transcription, splicing, mRNA transport, and mRNA degradation (Markmiller et al., 2014). To summarize the expression-level phenotypes of these models, knockdown of U6atac in *D. melanogaster* disrupted metabolism gene expression; knockdown of three different minor spliceosome-specific protein genes in *A. thaliana* caused abnormal gibberellic acid metabolism; and knockdown of *rnpc3* in zebrafish triggered abnormal mRNA processing (Kim et al., 2010; Pessa et al., 2010; Markmiller et al., 2014). Currently, it is unclear i) if these disparate effects are due to species-specific expression of MIGs; ii) if, as the findings from functional classification approaches suggest, MIGs perform disparate functions among all species tested; or iii) if the identity of the minor spliceosome component targeted for knockdown differentially affects MIGs.

In the past year, the use of conditional knockout approaches to study minor intron splicing has become more prevalent in the field. In 2018, we reported that deletion of U11 snRNA in the embryonic mouse cortex (pallium) resulted in severe microcephaly (Baumgartner et al., 2018). Both histological and RNAseq analyses of the mouse pallium revealed significant minor intron retention in cell cycle-related MIGs; numerous cell cycle defects, affecting S-phase, mitosis, and cytokinesis; DNA damage; and p53-mediated apoptosis, primarily in G1-phase progenitor cells (Baumgartner et al., 2018). In the developing cortex, there are two types of progenitor cells: the radial glial cells, which comprise the proliferative pool, and intermediate progenitor cells, which are produced from radial glial cells and divide to produce neurons (Noctor et al., 2004; Gotz and Huttner, 2005; Kowalczyk et al., 2009). Unexpectedly, U11 loss predominantly affected the self-amplifying radial glial cells, while radial glial cells undergoing differentiative divisions to produce intermediate progenitor cells/neurons, intermediate progenitor cells, and neurons were less affected (Baumgartner et al., 2018). This trend of cell type-specific sensitivity to minor spliceosome inactivation seemed to correspond to the degree of cell proliferation. Moreover, when Doggett et al. induced constitutive *Rnpc3* deletion in adult mice, the mutant mice displayed significantly lower levels of lymphocytes, monocytes, erythrocytes, and thrombocytes; decreased thymus size; and substantial degeneration of the lining of the gastrointestinal tract within a week of tamoxifen injection (Doggett et al., 2018). The degeneration of the gastrointestinal mucosa occurred alongside significant cell death and reduced cell proliferation (Doggett et al., 2018). Within eight days of *Rnpc3* deletion, these mice displayed signs of severe malnutrition, at which point they were euthanized (Doggett et al., 2018). While Doggett et al. did not investigate the effects of *Rnpc3* depletion on the hematopoietic stem cells of the red bone marrow, their findings also indicated that *Rnpc3* is required for normal hematopoiesis (Jagannathan-Bogdan and Zon, 2013; Doggett et al., 2018). These findings, combined with the embryonic lethality observed after complete and constitutive minor spliceosome inactivation, suggest that proper MIG expression is required for the survival of rapidly dividing cycling cell populations, while differentiated cells are less sensitive to minor spliceosome inactivation (Baumgartner et al., 2018; Doggett et al., 2018).

### Minor Intron-Containing Genes and Cycling Cell Survival

The observations that cycling cells are highly sensitive to minor spliceosome inactivation suggested to us a possible unifying feature shared by MIGs that is independent of the functions they execute: their requirement for cycling cell survival. Thus, we predicted that minor introns are more prevalent in genes essential for survival of cycling cells.

#### Minor Intron-Containing Genes Are Enriched in Genes Essential for Rapidly Cycling Cell Survival

Serendipitously, Meyers et al. of the Broad Institute have identified genes essential for the survival of various rapidly dividing cell lines, with correction for gene copy number per cell line (Doench et al., 2016; Cancer, 2017; Meyers et al., 2017). For this, the authors employed clustered regularly interspaced short palindromic repeats (CRISPR/Cas9) to disrupt the expression of most protein-coding genes in the human genome, one gene at a time, in order to determine their requirement for cell survival in 341 different cancer cell lines (Meyers et al., 2017). If MIGs are essential for the survival of cycling cells, then one would expect MIGs to be enriched in these essential genomes (essentialomes). To test this, we first extracted lists of 1) all genes interrogated by the guide RNA library employed, and 2) the genes comprising the 341 cell-line essentialomes (**Supplementary Methods**). The 341 cell-line essentialomes were also combined into a single, “total” essentialome of 4,360 genes, consisting of all genes shown to be essential for the survival of at least one cell line. In parallel, we extracted a list of all human MIGs from the MIDB (Olthof et al., 2019); any MIGs that were not interrogated by Meyers et al. were excluded from the downstream analysis, resulting in a list of 596 human MIGs.

**Figure 2:**
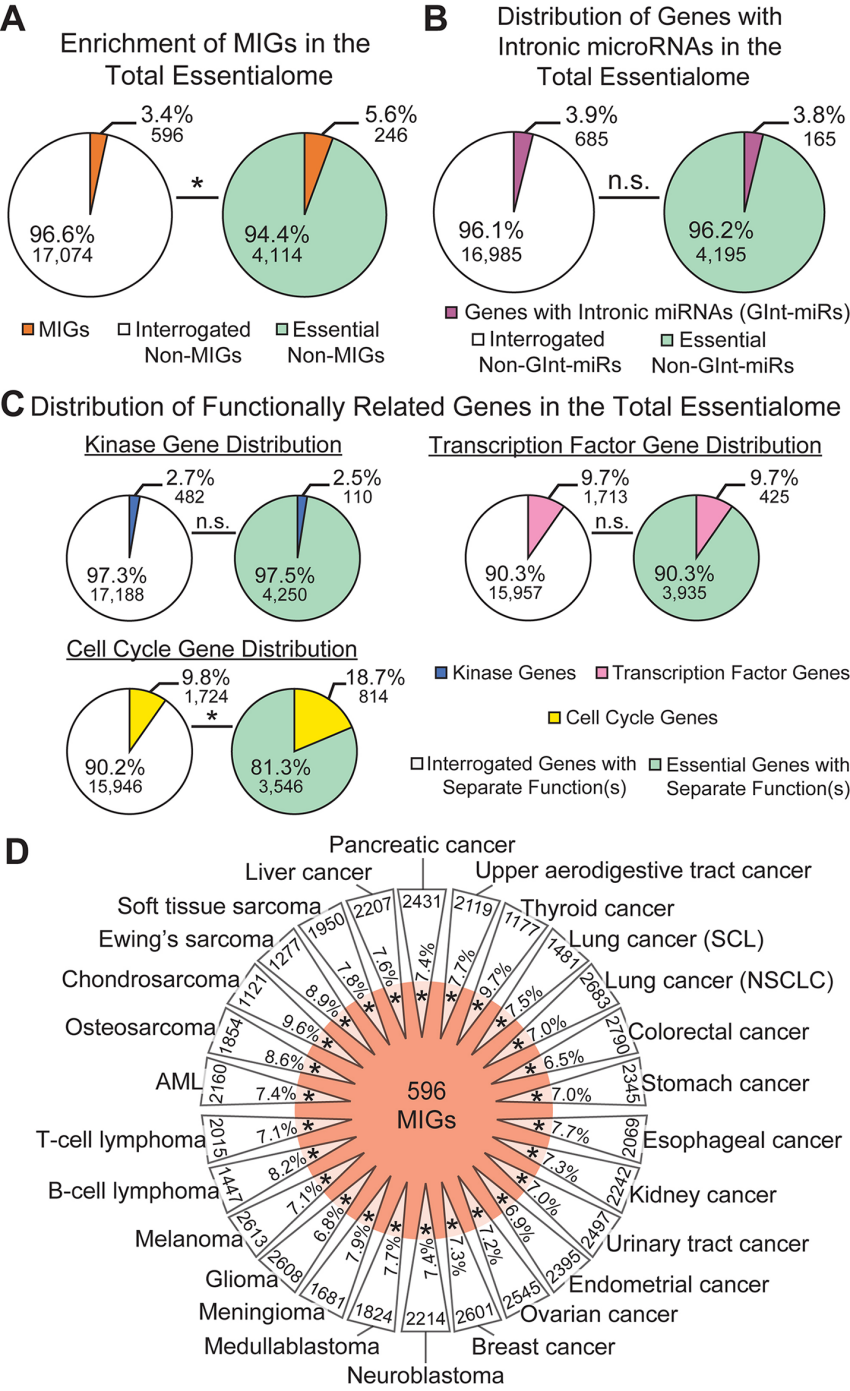
Minor intron-containing gene (MIGs) are enriched in the essentialome. **(A)** Pie charts showing the percentage of MIGs (orange) in all genes interrogated (left) and in all essential genes (right). **(B)** Pie charts showing the percentage of genes with intronic microRNA genes (GInt-miRs) in all interrogated genes (left) and in the total essentialome (right). **(C)** Pie charts showing the percentage of genes with kinase activity (top left), transcription factor activity (right), or a role in cell cycle regulation (bottom left) in all interrogated genes and in the total essentialome. **(D)** Daisy model representing the 26 cancer-type essentialomes (triangular petals) and the 596 MIGs (orange center). The overlap of the center with the petals indicates the enrichment of MIGs in the cell-line essentialomes, with the percentage of MIG enrichment in each petal. Gene number within each cancer-type essentialome is located on the outside of each petal. Statistical significance was determined by Fisher’s exact test. AML; acute myeloid leukemia, SCL; small cell lung cancer, NSCLC; non-small cell lung cancer. N.s., not significant; * *P* ≤ 5.45E−16.

Next, we examined the percentage of MIGs found in these gene sets. We observed significant enrichment of MIGs in the total essentialome (5.5%; *P* = 1.95E−19) compared to their proportion in the list of all genes interrogated (3.5%; **Figure 2A**). To further verify this enrichment, we sought to calculate the enrichment of a series of control gene lists, each linked by either a common function or a common feature, in the total essentialome. Since MIGs were chosen based on the presence of a specific intronic feature, we first sought to test whether genes sharing a different intronic feature would also be significantly enriched in the total essentialome. For this, we turned to microRNA (miRNA) genes. While some miRNA genes are found in intergenic regions of the genome, many miRNA genes are found embedded in the introns of protein-coding genes (Olena and Patton, 2010). Using the miRBase database (Kozomara and Griffiths-Jones, 2014), we extracted 685 protein-coding genes with intronic miRNA genes in the human genome. We did not observe significant enrichment for these genes in the total essentialome (3.8%; *P* = 0.75) compared to their proportion in the interrogated gene list (3.9%, **Figure 2B**), suggesting that a shared intronic feature alone does not explain the significant MIG enrichment we observe. We next sought to investigate the role of shared function on gene list enrichment in the total essentialome. In prior essentialome publications, genes involved in cell signaling were depleted in the essentialome, while genes regulating RNA processing, transcription, translation, and cell cycle were enriched (Blomen et al., 2015; Hart et al., 2015; Wang et al., 2015). Therefore, to generate two negative control lists, we extracted 482 human kinase genes (the kinome) and 1,713 human transcription factor genes from the Gene Ontology knowledgebase (The Gene Ontology, 2017). For a positive control list, we also extracted 1,724 human cell cycle genes (The Gene Ontology, 2017). As expected, analysis of either the human kinome (2.5%; *P* = 0.36) or the transcription factors (9.7%; *P* = 0.88) did not reveal significant enrichment in the total essentialome, compared to all interrogated genes (2.7 and 9.7%, respectively; **Figure 2C**). In contrast, cell cycle genes were significantly enriched in the total essentialome (18.7%; *P* = 2.56E−102), relative to the interrogated gene list (9.8%; **Figure 2C**). Together, these results suggest that the enrichment of MIGs in the total essentialome is likely driven by shared essential function, not by a shared intronic feature. We then further dissected the total essentialome by cell type, by combining the essentialomes of cell lines sharing the same cancer origin, which resulted in 26 cancer-type essentialomes (**Figure 2D**) (Meyers et al., 2017). Analysis of these cancer-type essentialomes revealed even higher enrichment of MIGs, ranging from 6.5 to 9.7% (**Figure 2D**; *P* ≤ 5.45E−16). This was mirrored by MIG enrichment in the individual, 341 cell-line essentialomes, which ranged from 6.6 to 10.1% (**Supplementary Table 1**; *P* ≤ 4.31E−12).

**Figure 3:**
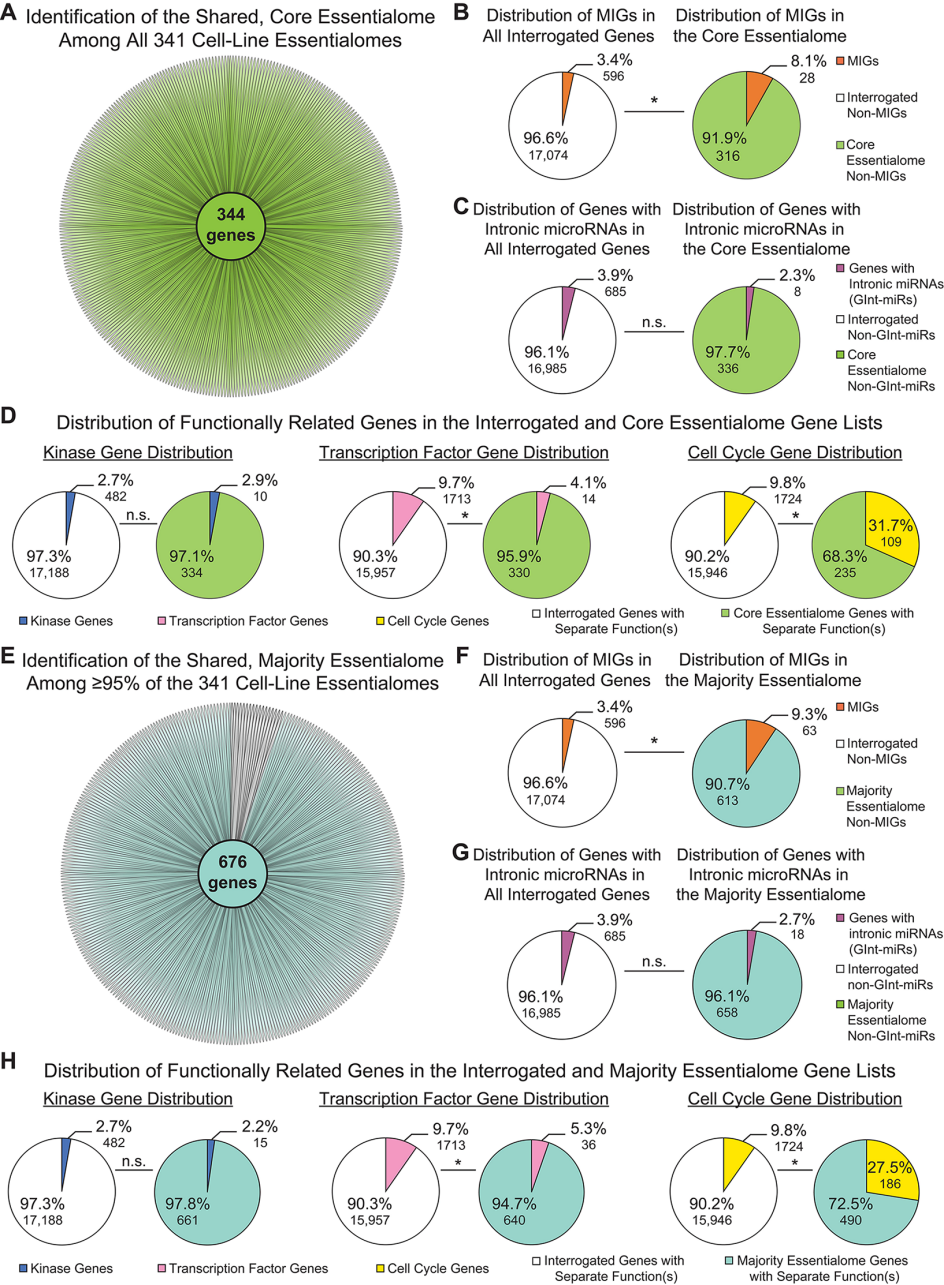
Minor intron-containing genes (MIGs) are enriched in shared essential genes. **(A)** Daisy model showing the number of essential genes common to all 341 cell-line essentialomes (“core essentialome”; green circle). Each petal corresponds to one cell-line essentialome. **(B)** Pie charts showing the percentage of MIGs in all interrogated genes (left) and in the core essentialome (right). **(C**) Pie charts showing the percentage of genes with intronic microRNA genes (GInt-miRs) in all interrogated genes (left) and in the core essentialome (right). **(D**) Pie charts showing the percentage of genes with kinase activity (leftmost), transcription factor activity (middle), or a role in cell cycle regulation (rightmost) in all interrogated genes and in the core essentialome. **(E)** Daisy model showing the number of essential genes common to the majority (≥95%) of the 341 cell-line essentialomes (“majority essentialome”; teal circle). **(F)** Pie charts showing the percentage of MIGs in all interrogated genes (left) and in the majority essentialome (right). **(G)** Pie charts showing the percentage of genes with intronic microRNA genes (GInt-miRs) in all interrogated genes (left) and the majority essentialome (right). **(H)** Pie charts showing the percentage of genes with kinase activity (leftmost), transcription factor activity (middle), or a role in cell cycle regulation (rightmost) in all interrogated genes and in the majority essentialome. Statistical significance was determined by Fisher’s exact test. N.s., not significant, * *P* ≤ 1.40E−04.

We observed little variation in MIG enrichment among the individual cell-line essentialomes. This could indicate that a core set of essential MIGs might be shared among the 341 cell-line essentialomes. To investigate this, we extracted 1) genes shared among all 341 cell-line essentialomes, producing a core essentialome of 344 genes (**Figure 3A**); and 2) genes shared among the vast majority (≥ 95%) of the 341 cell-line essentialomes (the majority essentialome), which produced a list of 676 genes (**Figure 3E**). In the core essentialome, we observed a significant, 2.4-fold MIG enrichment (8.1%; *P* = 2.35E−05) compared to the proportion of MIGs among all interrogated genes (3.4%, **Figure 3B**). Similarly, in the majority essentialome, MIG enrichment was significantly higher (2.7-fold, 9.3%; *P* = 2.29E−13) relative to the fraction of MIGs in all interrogated genes (3.4%, **Figure 3F**). We used the same control gene lists from the total essentialome analysis (**Figures 2B, C**) to further verify these enrichments in the shared essentialome gene lists. We did not observe significant enrichment for the genes with intronic miRNA genes or for the kinome in either the core essentialome (2.3%, *P* = 0.16; 2.9%, *P* = 0.74) or the majority essentialome (2.7%, *P* = 0.10; 2.2%, *P* = 0.47), compared to their proportions in the interrogated gene list (3.9 and 2.7%, **Figures 3C, D, G, H**). Transcription factors were significantly *depleted* in the core essentialome (4.1%; *P* = 1.40E−04) and the majority essentialome (5.3%; *P* = 3.62E−05), relative to the interrogated gene list (9.7%; **Figures 3D, H**). In contrast, cell cycle genes were significantly enriched in both the core essentialome, with a 3.2-fold increase (31.7 *vs.* 9.8%, *P* = 4.13E−30; **Figure 3D**), and the majority essentialome, with a 2.8-fold increase (27.5 *vs.* 9.8%, *P* = 2.71E−41; **Figure 3H**). These results further underscore that MIG enrichment in these essential gene sets is likely driven by shared essential functions.

#### Most Essential Minor Intron-Containing Genes Trace Back to the Last Eukaryotic Common Ancestor and Are Enriched in the Ancient Essentialome

**Figure 4:**
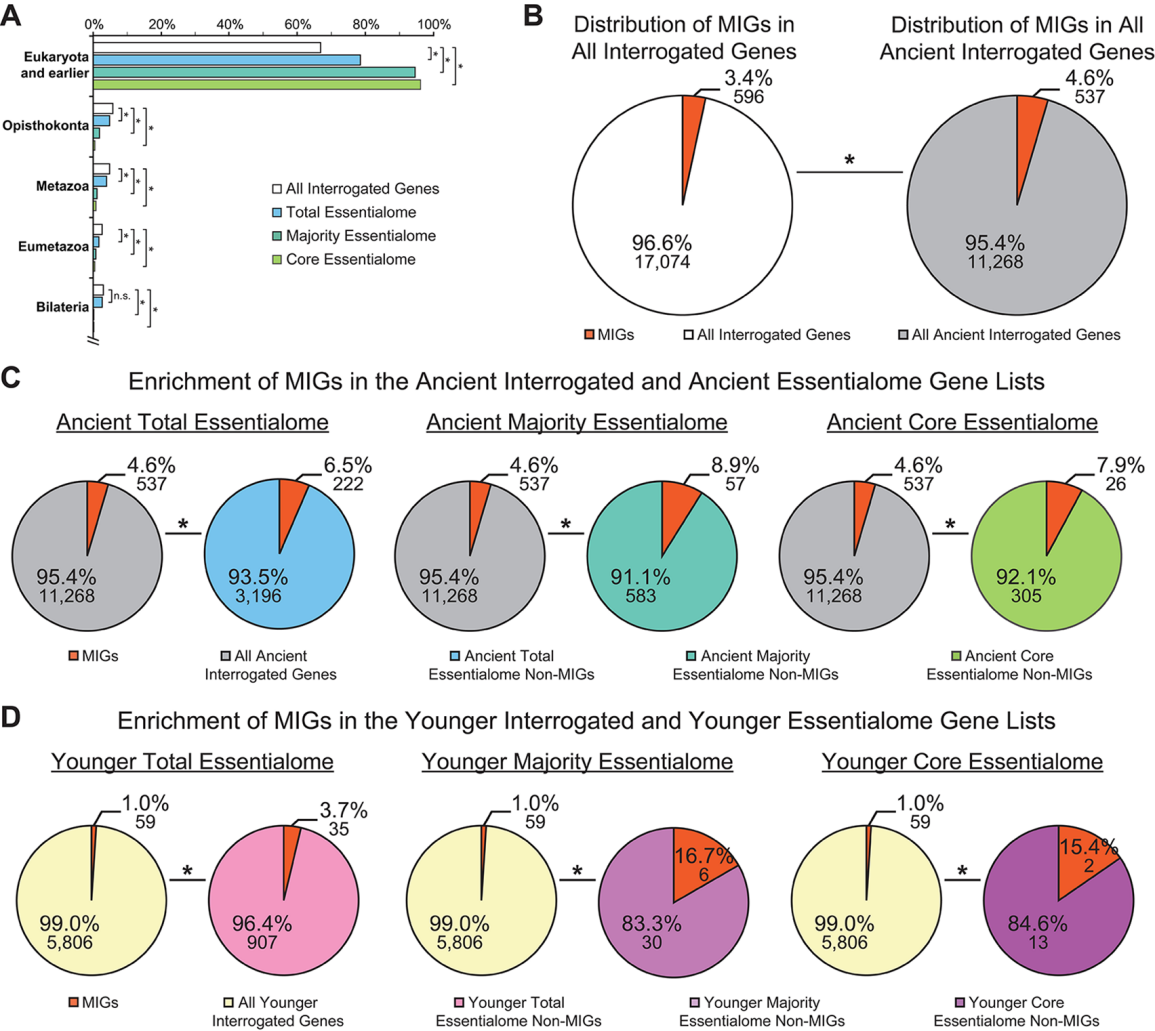
Minor intron-containing genes (MIGs) are enriched in essential genes regardless of age. **(A)** Evolutionary age of all interrogated genes (white), the total essentialome (blue), the majority essentialome (teal), and the core essentialome (green). Clades are listed on the y-axis. The x-axis shows the percentage of genes that can be traced to the listed clade, but not to an older clade. **(B)** Pie charts showing the enrichment of MIGs (orange) in the all interrogated genes (left) and all ancient genes (tracing back to *Eukaryota* and earlier; right). **(C)** Pie charts showing the enrichment of MIGs (orange) in the ancient total essentialome (blue, left), the ancient majority essentialome (teal, middle), and the ancient core essentialome (green, right), compared to all ancient interrogated genes (gray). **(D)** Pie charts showing the enrichment of MIGs (orange) in the younger (tracing back to *Opisthokonta* or later) total essentialome (pink, left), the younger majority essentialome (light purple, middle), and the younger core essentialome (dark purple, right), compared to all younger interrogated genes (yellow). Statistical significance, relative to the percentage of MIGs present in the respective interrogated gene lists, was determined by Fisher’s exact test. N.s., not significant; * *P* ≤ 0.007.

In 2015, two groups performed similar gene essentiality studies on a handful of cancer cell lines, then extracted the core essentialome of the cell lines they independently investigated. When Hart et al. analyzed the core essentialome of the five cell lines they studied, they observed enrichment of genes that overlapped with essential genes in yeast, *C. elegans*, *D. melanogaster*, and mouse. Likewise, when Blomen et al. extracted the core essentialome of the two cell lines they investigated, they identified a subset of ancestral genes. Therefore, we sought to interrogate whether the essential genes we identified—those in the total essentialome, those in the majority essentialome, and those in the core essentialome—were similarly ancient. For this, we employed a similar ortholog identification approach as that used by Blomen et al., which utilized the evolutionary genealogy of genes: Non-supervised Orthologous Groups (eggNOG) database to identify ancient essential genes (Huerta-Cepas et al., 2019). We found that significantly more of the genes in the total essentialome (78.4%, *P* = 3.49E−82), the majority essentialome (94.7%, *P* = 5.49E−72), and the core essentialome (96.2%, *P* = 4.06E−42) trace back to the last eukaryotic common ancestor (LECA) or further, compared to all interrogated genes (66.8%; **Figure 4A**). Thus, even within the modern essentialome, there is an ancient essentialome consisting of genes present in or before LECA, and a younger essentialome that emerged in *Opisthokonta* or later.

It is widely accepted that MIGs are ancient, with only one known example of recent minor intron gain (Lin et al., 2010). To verify this, we determined the prevalence of the 596 MIGs in all ancient interrogated genes, compared to the prevalence of MIGs in all interrogated genes. As expected, the majority (90.1%) of MIGs were ancient, and they were significantly enriched in the ancient gene list (4.6 *vs.* 3.4%, *P* = 1.46E−41; **Figure 4B**). When we investigated the enrichment of essential MIGs (350) and non-essential MIGs (246) in the ancient gene list, we observed significant enrichment of both essential MIGs (1.9 *vs.* 1.4%; *P* = 6.78E−18) and non-essential MIGs (2.7 *vs.* 2.0%; *P* = 2.13E−24), compared to all interrogated genes. Thus, regardless of whether MIGs are essential, they are more likely to be ancient than expected by random chance. Similarly, essential genes are more likely to be ancient than expected by random chance (**Figure 4A**). Therefore, it is possible that MIG enrichment in essential gene lists is due to their age, rather than their shared essential functions. To remove age as a potential variable, we considered only ancient interrogated genes, ancient essential genes, and ancient MIGs in our analysis. Compared to their prevalence in the ancient interrogated gene list (4.6%), ancient MIGs were significantly enriched in the ancient total essentialome (6.5%; *P* = 2.41E−05), the ancient majority essentialome (8.9%; *P* = 1.15E−06), and in the ancient core essentialome (7.9%; *P* = 0.007; **Figure 4C**). Therefore, age alone does not explain the enrichment of MIGs in these essential gene lists. To further test whether MIG age impacts MIG enrichment in essential gene lists, one could assess the prevalence of MIGs in the younger essentialome. For this, we turned to the MIGs that were not ancient.

#### Nearly 10% of Minor Intron-Containing Genes Emerged After the Last Eukaryotic Common Ancestor and Are Enriched in Younger Essential Genes

Of all interrogated MIGs, 59 (9.9%) did not trace back to LECA or earlier. These younger MIGs had a wide variety of ages, tracing back to *Opisthokonta* (13 MIGs), *Metazoa* (12), *Eumetazoa* (12), *Bilateria* (10), *Deuterostomia* (2), *Chordata* (3), *Euteleostomi* (6), and *Amniota* (1; **Supplementary Table 2**). We sought to determine whether these younger MIGs were enriched in younger essential genes, i.e., those tracing back to *Opisthokonta* or later. To control for gene age, we used all younger interrogated genes as the background for our analyses. Strikingly, this approach revealed significant enrichment of MIGs in the younger total essentialome (3.7%; *P* = 3.93E−14), the younger majority essentialome (16.7%; *P* = 1.23E−06), and the younger core essentialome (15.4%; *P* = 0.007), relative to all younger interrogated genes (1.0%; **Figure 4D**). This pattern of younger MIG enrichment in the younger essential gene lists echoed the pattern of ancient MIG enrichment in the ancient gene lists (**Figures 4C, D**). Therefore, regardless of their age, MIGs are significantly enriched in essential genes.

#### Many Shared Essential Minor Intron-Containing Genes Function in Cell Cycle Regulation

To investigate the biological processes performed by the MIGs found in the majority essentialome and the core essentialome, we manually curated the literature on all MIGs of the majority essentialome and extracted their functions (**Supplementary Table 3**). Of the 63 MIGs of the majority essentialome, nearly half (26 genes) function in cell cycle regulation; moreover, 14 of these 26 MIGs are specifically involved in mitosis (**Supplementary Table 3**). The next largest functional category is transcription and its regulation, in which 16 MIGs function, followed by RNA processing, comprising 15 MIGs. These 15 MIGs can be further subdivided into two categories: those functioning in pre-mRNA splicing (11 MIGs) and those that regulate RNA metabolism (4 MIGs). The fourth largest functional category is non-coding RNA biogenesis, which is regulated by nine MIGs. The remaining functional classifications are listed in **Supplementary Table 3**. When focusing on the 28 MIGs of the core essentialome, the distribution of MIG functions is similar: the most common functions are cell cycle (11 MIGs, 5 of which regulate mitosis), RNA processing (9 MIGs, most of which regulate mRNA splicing), and transcription and its regulation (8 MIGs). However, the fourth most common function of the core essentialome MIGs is translation (5 MIGs), whereas only a single MIG in the core essentialome regulates non-coding RNA biogenesis (**Supplementary Table 3**).

Of the 63 MIGs in the majority essentialome, 57 trace back to LECA or earlier. The six younger MIGs are *AHCTF1*, which is required for proper mitosis progression; *C1orf109*, a cell cycle regulator; *C3orf17*, a gene that maintains cortical neural progenitor identity by inhibiting differentiation; *MTBP*, which regulates both DNA replication and mitosis; *SPC24*, a gene encoding a component of the kinetochore; and *TAF1C*, which regulates rRNA transcription (**Supplementary Tables 2** and **3**). Of these, *MTBP* and *SPC24* were also identified in the core essentialome. For both the ancient majority essentialome and the ancient core essentialome, the most common MIG functions are cell cycle (21 MIGs and 9 MIGs, respectively), transcription and its regulation (16 and 8 MIGs), RNA processing (15 and 9 MIGs), translation (8 MIGs in both), and, for the ancient majority essentialome, non-coding RNA biogenesis (8 MIGs) (**Supplementary Table 3**).

#### Minor Intron-Containing Genes Are Expressed Throughout the Cell Cycle

**Figure 5:**
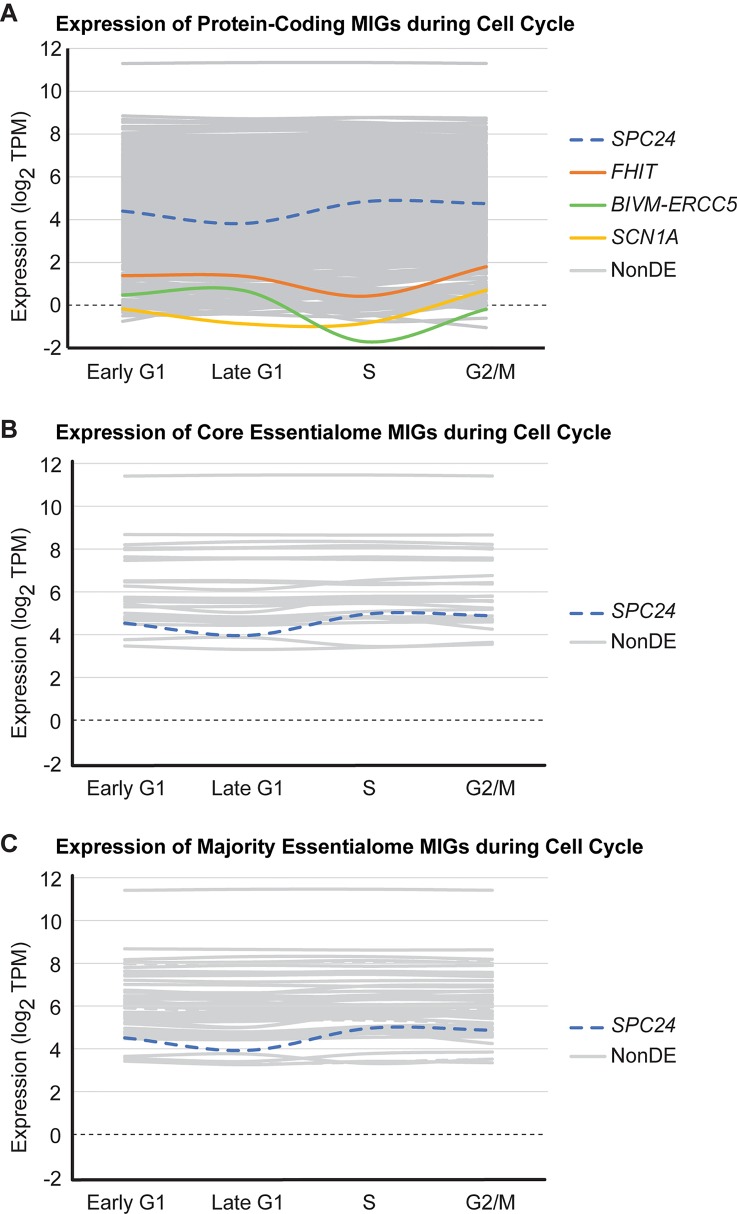
Most minor intron-containing genes (MIGs) are expressed stably during cell cycle. Data plotted for **(A)** 624 protein-coding MIGs, **(B)** 28 core essentialome MIGs, and **(C)** 63 majority essentialome MIGs deemed to be expressed during cell cycle (as determined by TPM≥1 in at least one stage of cell cycle). Data obtained from analysis of RNAseq generated by Singh et al. (2013). Each line represents the expression of one MIG throughout successive stages of cell cycle (early G1, late G1, S, and G2/M). Colored lines indicate MIGs that showed differential expression in at least one stage of cell cycle relative to the previous stage, gray lines are MIGs that are non-differentially expressed (NonDE) in all stages, and dashed lines represent MIGs in the younger essentialome.

Given that i) the shared essential genes were derived from experiments using rapidly dividing cancer cell lines, and ii) cell cycle-regulating MIGs are enriched in all shared essential gene lists interrogated, we suspected that the shared essential MIGs are highly expressed throughout all phases of the cell cycle. Moreover, we have previously shown that inactivation of the minor spliceosome in cortical progenitor cells causes cell cycle defects in multiple cell cycle phases (Baumgartner et al., 2018). To test this prediction, we leveraged RNAseq data from Singh et al. (2013), who used the fluorescence ubiquitination cell cycle indicator (FUCCI) plasmid to obtain four populations of human embryonic stem cells—those in early G1, late G1, S, and G2/M—through fluorescence activated cell sorting (**Supplementary Methods**). We found that 577 MIGs are expressed (as determined by transcripts per million (TPM) ≥ 1) throughout all stages of the cell cycle (**Figure 5A**). Moreover, the expression of MIGs is highly stable. Only 4 of the 624 MIGs expressed in at least one stage of cell cycle (0.64%) were differentially expressed at any stage of cell cycle relative to the previous stage (**Figure 5A**). Out of the 28 core essentialome MIGs, all are expressed in each stage, and only one MIG (*SPC24*, a younger MIG) shows differential expression (from late G1 to S; *P* < 0.05) (**Figure 5B**, **Supplementary Table 2**). Similarly, when we extended this analysis to the majority essentialome, all 63 MIGs were expressed at each stage of the cell cycle, and only *SPC24* showed differential expression (**Figure 5C**). We argue that the high percentage of MIGs expressed throughout cell cycle at a stable level is indicative of their essentiality for proper cell cycle progression.

### Incorporating Minor Intron Conservation Into a Revised Parasitic Minor Intron Invasion Model

The conservation of minor introns and the minor spliceosome through early eukaryotic evolution has remained enigmatic. Despite the disadvantages associated with minor introns and the link between large, unicellular populations and strong purifying selection, minor introns and the minor spliceosome have persisted through the unicellularity of early eukaryotic evolution. Here, we found that MIGs are significantly enriched in ancient, essential genes, even after controlling for MIG age (**Figure 4C**). The presence of minor introns in crucial genes of the ancestral eukaryotic genome would necessitate the conservation of the minor splicing machinery. Moreover, the enrichment of MIGs in ancient genes of the modern essentialome further indicates that minor introns in these ancient, essential genes were highly conserved across eukaryotic evolution. Therefore, we propose a revision of the parasitic minor intron invasion model to encompass this pattern of minor intron conservation. As described in the original parasitic minor intron invasion model, minor introns were randomly distributed across the genome, in both non-essential and essential genes consisting primarily of major introns. This gene organization would result in inefficient splicing and reduction in the amount of mRNA encoding full-length proteins. This would produce strong selective pressure to remove these minor introns from the genome, consistent with observations of progressive minor intron loss across eukaryotic evolution, *via* fusion of exons neighboring minor introns or mutation-based conversion of minor introns into major introns (Burge et al., 1998; Bartschat and Samuelsson, 2010; Lin et al., 2010). Both mechanisms of minor intron loss can cause perturbation in MIG expression levels or the production of aberrant proteins, ultimately impacting the functions executed by MIG-encoded proteins. In particular, the sequential mutations required for minor-to-major intron conversion would likely produce intermediate, weak consensus sequences, severely impairing gene expression (Burge et al., 1998; Lin et al., 2010). For the organism, the risk associated with minor intron loss in a specific MIG would depend on the importance of that MIG’s function for survival. For example, for a MIG executing a non-essential function, minor intron loss-associated perturbations in expression would have little impact on organism survival. In contrast, for a MIG required for survival, minor intron loss-associated perturbations would have a much higher risk to organism survival. Thus, one would expect limited loss of the minor introns in these essential genes. Indeed, our data support this idea, as we observed significant enrichment of MIGs in all essentialome lists, particularly in the genes shared among essentialomes (**Figures 2A, C**, **3B, F**, and **4C**). We argue that the presence of minor introns in ancient genes essential for cycling cell survival and cell cycle progression ensured the maintenance of minor introns and the minor spliceosome in early eukaryotic evolution. Specifically, reproduction in early, unicellular eukaryotes would require proper cell cycle progression and the survival of the newly born unicellular organisms post-division. Therefore, the presence of minor introns in genes essential for both of these processes would guarantee the conservation of minor introns and the minor spliceosome through the unicellular period of early eukaryotic evolution.

In addition to these ancient minor introns, our data suggest that subsequent minor intron gain events may have impacted the maintenance of minor introns in eukaryotic evolution. We identified 59 MIGs that did not trace back to LECA, indicating that these genes gained minor introns after the initial parasitic minor intron invasion (**Supplementary Table 2**). These younger MIGs were also significantly enriched in the younger essential gene lists (**Figure 4D**). Moreover, the most common function performed by these 59 younger MIGs is cell cycle regulation, similar to the trend observed in the ancient shared essentialome MIG lists (**Supplementary Tables 2** and **3**). Thus, we propose that these minor intron gain events further raised the prevalence of minor introns in genes essential for cycling cell survival, thereby increasing the conservation of minor introns and the minor spliceosome throughout eukaryotic evolution.

The identification of these younger MIGs also necessitates a major shift in the minor splicing field. Due to the stringency of the minor-class consensus sequences, it had been assumed that minor intron gain is extremely rare; indeed, only one minor intron gain event had been previously identified (Lin et al., 2010). However, in our human MIG-specific analysis, we have identified dozens of new candidate minor intron gain events. Notably, only 7 of the 59 younger MIGs represent minor intron-rich gene families, including the *CRTC* (3 MIGs), *PROX* (2 MIGs), and *ERICH* (2 MIGs) families, where a single minor intron gain event could produce multiple MIGs *via* ancestral MIG duplication (**Supplementary Table 2**). Thus, when counting minor intron gain events, each of these minor intron-rich gene families can be collapsed to a single minor intron gain event in its respective ancestral gene. With these minor intron-rich gene families consolidated, we have identified 55 candidate minor intron gain events in this branch of eukaryotic evolution. Since our eggNOG-based approach can only estimate gene age, not the age of minor introns, it is unclear when or the mechanism by which these minor intron gain events occurred in this eukaryotic lineage. However, we suspect that these minor intron gain events occurred across a wide swath of eukaryotic evolution, since the ages of these younger MIGs range from *Opisthokonta* (∼1.1 billion years) to *Amniota* (∼300 million years) (**Supplementary Table 2**) (Kumar et al., 2017). Together, these findings indicate that minor intron evolution is far more dynamic than previously thought, even within a single eukaryotic lineage, and raise myriad questions about the mechanisms of minor intron gain, how minor intron gain and minor intron loss rates correlate across eukaryotes, and the potential impact of minor intron gain on alternative splicing regulation.

Ultimately, our model of minor intron emergence and evolution emphasizes the importance of studying minor intron conservation and MIG function in concert, particularly in organisms with few MIGs—i.e., species in lineages that have undergone profound minor intron loss/conversion. We also raise the possibility that the rates of minor-to-major intron conversion may differ between essential *versus* non-essential genes, particularly compared to the rates of complete minor intron loss in these gene populations. We hope this model, and our identification of 55 candidate minor intron gain events, will spark new investigations into the evolution of minor intron splicing, a subfield of minor splicing research that has been relatively dormant in recent years.

### A Role for Minor Splicing in Multicellular Organism Evolution?

**Figure 6:**
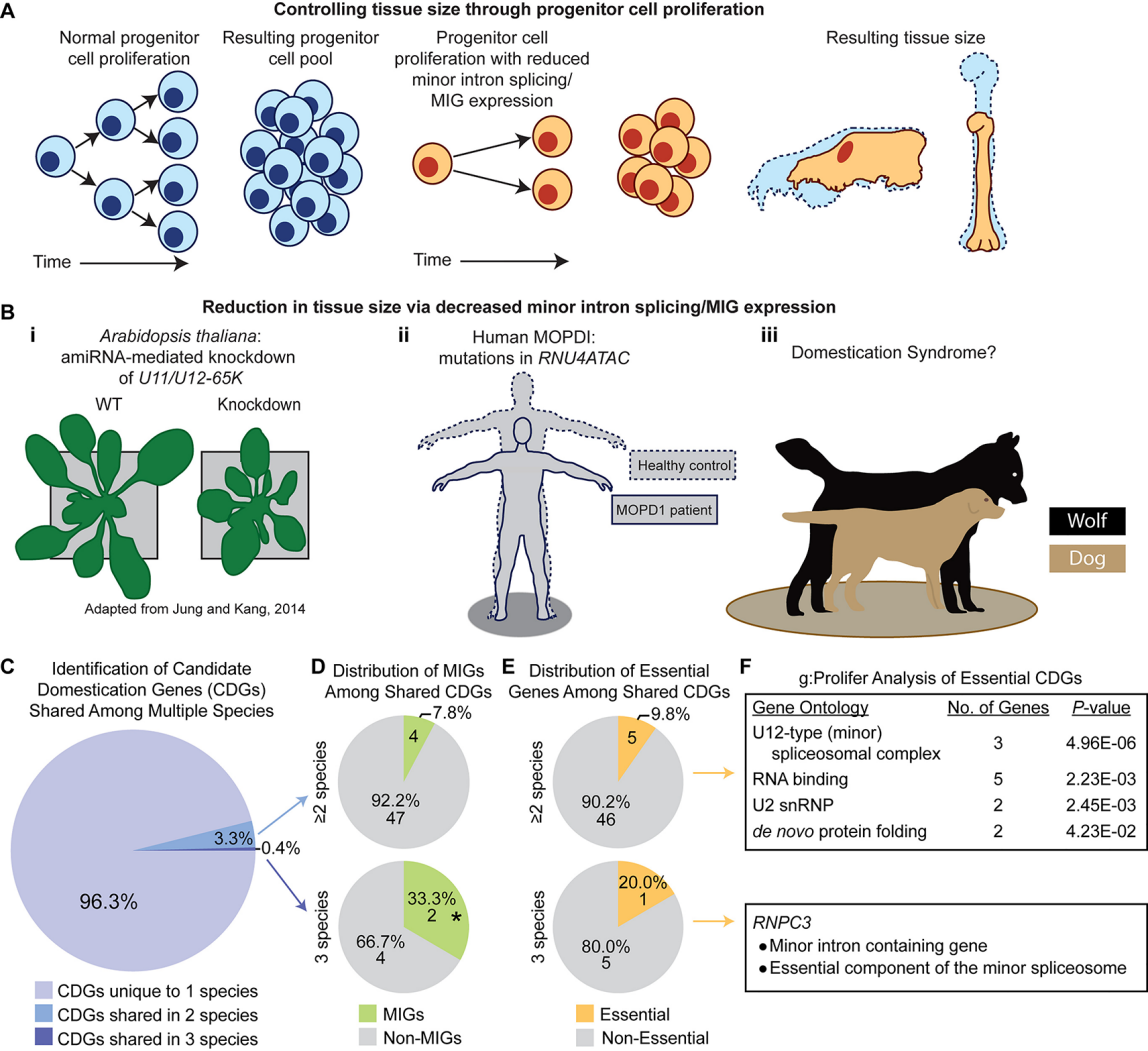
A potential role for the minor spliceosome in domestication. **(A)** A model demonstrating how tissue size can be controlled through progenitor cell proliferation. **(B)** Evidence for tissue size reduction upon impairment of minor intron splicing/minor intron-containing gene (MIG) expression. **i**) Plant size is smaller in *A. thaliana* upon knockdown of the minor spliceosome-specific *U11/U12-65K*. Reproduced with permission from Jung and Kang, 2014. **ii**) Mutations in *RNU4ATAC* cause three diseases in human, such as microcephalic osteodysplastic primordial dwarfism type 1 (MOPD1), wherein patients’ display reductions in multiple tissues causing a severely reduced body plan relative to healthy controls (theoretical output). **iii**) Previous studies suggest a link between reduced minor intron splicing/MIG expression and domestication syndrome, of which one prominent example is the domestication of dog (beige) from wolf (black). **(C)** Pie-chart representing the distribution of candidate domestication genes (CDGs) from dog, cat, cattle, horse, and anatomically modern human curated from Theofanopoulou et al. (2017) that are unique to one species (light purple), overlap in two species (blue), or are shared among three species (dark purple). **(D)** Pie-chart showing the number of MIGs (green) or non-MIGs (gray) found within the list of CDGs shared between two (top) or three (bottom) species. **(E)** Pie-chart showing the number of essential (yellow) or non-essential (gray) genes found within the list of CDGs shared between two (top) or three (bottom) species. **(F)** Description of gene enrichment (as determined through g:Profiler) for essential genes shared among two or three species (only one gene, *RNPC3*, is both essential and a shared CDG among three species). Significance determined by Fisher’s exact test; * *P* < 0.05.

In unicellular organisms, cell division is utilized for reproduction. However, in multicellular organisms, cell division has been repurposed for organism growth and the production of specialized structures, tissues, and cells within a single organism. Therefore, the requirement of MIG expression for cycling cell survival and the preponderance of MIGs regulating the cell cycle implicates minor splicing in multicellular evolution, particularly in tissue and organism size. During the growth process, a pool of progenitor cells must undergo proliferation, to increase the size of the progenitor cell pool, followed by differentiation, to produce cell types with more limited proliferative potential. These differentiated cells will comprise specialized tissues in the mature organism. The more progenitor cells that are present at the start of growth, the more differentiated cells can be produced, thus increasing organism or tissue size. If the growth process starts with a small population of progenitor cells, or those progenitor cells divide at a slow rate, then fewer differentiated cells will be produced, and organism/tissue size could be reduced. If MIG expression is required to ensure the survival of this proliferative pool, or if MIG expression is necessary for efficient cell cycle progression, then the efficiency of minor intron splicing could profoundly impact the size of the proliferating population (**Figure 6A**). For example, if the expression of the minor spliceosome components were suppressed to a low level, MIG splicing would be highly inefficient, limiting MIG expression. Given the functions of MIGs, this could trigger increased progenitor cell death and/or slowing of the cell cycle. In either case, the proliferative pool would shrink, thereby restricting organism growth. If suppression of minor splicing were restricted to specific progenitor niches, these progenitor pools would shrink and produce fewer differentiated cells, ultimately scaling down the size of derived tissues/features (**Figure 6A**). Therefore, we hypothesize that minor splicing represents a powerful target for controlling the scale of an organism and/or its tissues/features.

From this theory, one would expect that suppression of minor splicing activity would result in decreased organism and/or tissue size. In fact, knockdown of minor spliceosome-specific proteins in *A. thaliana* causes reduced overall size, both in height and width (Kim et al., 2010; Jung and Kang, 2014; Xu et al., 2016) (**Figure 6B**). Moreover, minor spliceosome disruption in humans is linked to multiple developmental disorders, all of which are associated with a combination of growth defects and brain hypoplasia (Edery et al., 2011; He et al., 2011; Merico et al., 2015; Farach et al., 2018). Three of these diseases—microcephalic osteodysplastic primordial dwarfism type 1 (MOPD1), Roifman syndrome (RS), and Lowry-Wood syndrome (LWS)—result from mutation in *RNU4ATAC*, which encodes the U4atac snRNA (Edery et al., 2011; He et al., 2011; Merico et al., 2015; Farach et al., 2018). The cardinal symptoms of these diseases are microcephaly, micrognathia, and primordial dwarfism, each of which represents a reduction in tissue size with severity proportional to the suspected level of minor spliceosome inhibition (MOPD1 > RS ≥ LWS) (Edery et al., 2011; He et al., 2011; Merico et al., 2015; Farach et al., 2018). This suggests that the level of minor spliceosome activity has direct consequences on organ/tissue size, which we suspect is mediated through regulation of progenitor population size. If true, we suspected that regulation of minor splicing activity and/or MIG expression would underlie evolution-driven tissue reduction in other lineages, of which one well-studied example is in animal domestication (**Figure 6B**).

In domestication, animals are bred based on their tameness, yet they develop a suite of seemingly disparate phenotypic traits consisting of smaller cranial capacity, micrognathia, skeletal shortening, floppiness of ears, curling of the tail, depigmentation, and neoteny (Wilkins et al., 2014). Together, these traits are referred to as animal domestication syndrome (Wilkins et al., 2014). It is thought that tameness is driven by deficits in neural crest cell amplification, which reduces the size of the adrenal medulla (Wilkins et al., 2014). Consequently, other neural crest-derived tissues, such as the brain, craniofacial skeleton, ear cartilage, and skin (specifically melanocytes), also receive reduced cellular input and thus undergo population/size reduction (Wilkins et al., 2014). This phenomenon of domestication-driven size reduction parallels the phenotypic output seen in diseases caused by deficits in minor intron splicing, as described in MOPD1, Roifman syndrome, and Lowry-Wood syndrome. Thus, we hypothesized that MIGs would be integral genes selected for in the animal domestication process.

To interrogate this hypothesis, we referred to a comparative genomics study by Theofanopoulou et al. (2017), who curated lists of candidate domestication genes for dog, cat, cattle, horse, and anatomically modern humans (*Homo sapiens*), which Theofanopoulou et al. argue have undergone self-domestication compared to other members of *Homo*, such as Neanderthals and Denisovans. We first sought to determine the prevalence of MIGs in the list of candidate domestication genes for each species. We found that MIGs comprise 6.6% of candidate domestication genes in dog, 2.4% in cat, 4.1% in cattle, 8.3% in horse, and 4.5% in human. For the human candidate domestication genes, MIGs were not significantly enriched by Fisher’s exact test (4.5%, 33 of 742; *P* = 0.0535), when compared to the prevalence of MIGs among all protein-coding genes in the human genome (3.2%, 648 of 20,444). Since the number of MIGs in the genomes of the remaining species is unknown, statistical analysis of MIG enrichment in the candidate domestication genes of these species could not be determined. Out of the 1,386 candidate domestication genes curated from all five species, 62 (4.5%) are MIGs. We found that MIGs were significantly enriched among these candidate domestication genes by Fisher’s exact test (4.5%; *P* = 0.0067), when compared to the prevalence of MIGs among all human protein-coding genes (3.2%, 648 of 20,444). The targeted selection of MIGs in domestication becomes apparent as one begins to identify parallel selection pressures overlapping multiple species. There are 51 candidate domestication genes that are shared among at least two species; 4 of these are MIGs (7.84%; *BRAF, CACNA1D, RNPC3, VEZT*) (**Figure 6D**). Moreover, six candidate domestication genes are shared among three species; two of these are MIGs (33.3%; *RNPC3, BRAF*) and this enrichment is significant by Fisher’s exact test (*P* = 0.02; **Figure 6D**).

We next sought to determine whether candidate domestication genes were also found within the essentialome. Out of the 51 candidate domestication genes shared among at least two species, 5 (*RNPC3, HSPD1, HSPE1, SF3B1, SNRPD1*) are found in the essentialome (**Figure 6E**). To identify the cellular pathway enriched by these genes, we employed g:Profiler, which revealed U12-type (minor) spliceosome as the top hit (**Figure 6F**) (Reimand et al., 2007). Moreover, of the six candidate domestication genes shared among three species, one gene (*RNPC3*) is also found in the essentialome (**Figures 6E, F**). *RNPC3* is both a MIG and a crucial component of the minor spliceosome (Benecke et al., 2005; Olthof et al., 2019). Taken together, this data suggests that domestication may act by targeting MIGs, the minor spliceosome, or both.

One can imagine that if these genes truly play a role in driving domestication, mutations in these genes in human may result in phenotypes closely resembling domestication syndrome. To understand this better, we explored diseases caused by candidate domestication MIGs, as well as candidate domestication MIGs from the same gene family, whose selection overlaps in multiple species. An example of the latter is *SLC9A6*, a candidate domestication gene in dog, and *SLC12A5*, a candidate domestication gene in human (Theofanopoulou et al., 2017). Both genes are members of the solute carrier family, which regulates ion exchange and thus membrane potential (Gilfillan et al., 2008; Ohgaki et al., 2011). Alterations in these genes may influence neural plasticity, specifically through glutamate metabolism, which has been suggested to be a key access point in dog domestication *via* reductions of fear responses toward humans (Li et al., 2014). In addition, mutation in *SLC9A6* causes Christianson syndrome in humans, which is characterized by neoteny, microcephaly, ataxia, and craniofacial defects (Gilfillan et al., 2008; Schroer et al., 2010).

In addition to *SLC*-family genes, the *TCTN* family shows overlap in selection: *TCTN3* is a candidate domestication gene in dog, and *TCTN1* is a candidate domestication gene in horse (Theofanopoulou et al., 2017). These genes encode proteins that constitute the tectonic-like complex, which is involved in mediating Hedgehog signal transduction as well as protein trafficking (Reiter and Skarnes, 2006; Gong et al., 2018). Expression of the tectonic-like complex is known to be critically important for neural tube development, and mutations in both *TCTN3* and *TCTN1* result in Joubert syndrome (Huppke et al., 2015; Wang et al., 2018). Patients with Joubert syndrome display intellectual disability due to underdevelopment of the brain, specifically the cerebellar vermis and brainstem, as well as craniofacial defects (Huppke et al., 2015). Again, these deficits strongly correlate with the morphological changes observed in domesticates relative to their wild counterparts (Wilkins et al., 2014).

Given that *BRAF* and *RNPC3* show selection in three species, we consider them the top two candidate domestication MIGs. BRAF is involved in the MAPK/ERK signaling pathway, which promotes cell proliferation (Sumimoto et al., 2006). Defects in BRAF function would disrupt progenitor cell amplification. In human, mutation in *BRAF* results in multiple types of cancers, as well as Noonan syndrome and Costello syndrome, both of which share characteristics of short stature, skeletal abnormalities, craniofacial defects, and heart malfunction (Davies et al., 2002; Tartaglia et al., 2011). Moreover, mutation in *RNPC3*, which encodes the minor spliceosome-specific U11/U12-65K protein, results in isolated growth hormone deficiency (IGHD) (Argente et al., 2014; Norppa et al., 2018). Patients with IGHD have short stature due to pituitary hypoplasia and thus reduced growth hormone production (Argente et al., 2014). Consistently, mutations in genes suspected to drive domestication syndrome produce diseases with overlapping phenotypes, highlighting the potential mechanism by which MIGs and/or the minor spliceosome may have been co-opted for evolutionary diversification.

The evidence we have presented highlights the consistent relationship between minor spliceosome and/or MIG inhibition and tissue size reductions. However, one can imagine that increasing the levels of minor spliceosome activity, and therefore MIG expression, may be able to increase progenitor cell proliferation, thereby driving an increase in tissue size, which has been observed in the domestication of land plants (i.e., crop plants) (Doebley et al., 2006). Unfortunately, current genomic data is insufficient to identify and cross-reference candidate domestication MIGs across domesticated land plants to that of animals, but is of intrigue for future studies.

### Implications for Our Understanding of Minor Splicing Evolution

Most research on the emergence and evolution of minor introns and minor splicing spanned the late 1990s until approximately 2012. Since 2012, there has been an explosion of research into the biological role of minor splicing, particularly in development and disease. Here, we have revisited models of minor splicing emergence and conservation through the lens of these new disease and developmental findings, which has led us to propose 1) that MIGs are linked by their requirement for cycling cell survival, 2) a revised model for minor intron emergence and conservation, and 3) that minor intron gain is far more common in eukaryotic evolution than previously appreciated. The predictions of this model, along with the identification of an ancient core essentialome, open new avenues of research into the conservation of these specific MIGs and their minor introns across eukaryotic evolution. Moreover, our identification of 55 candidate minor intron gain events paves the way for novel analyses of minor intron gain rates across eukaryotic lineages and the mechanism(s) of their emergence. We also hope our theories will encourage increased interdisciplinarity in the minor splicing field, where the majority of work has been either biochemical or bioinformatical.

We propose a novel role of minor splicing in multicellular evolution, which addresses the high conservation of minor introns in the plant and animal lineages, both of which are rich in multicellular life (Becker and Marin, 2009; Bartschat and Samuelsson, 2010; Sebe-Pedros et al., 2017). In particular, we argue that minor splicing is a target that, when manipulated, powerfully regulates proliferation in progenitor cells, thereby regulating organism growth in the evolution of multicellular life. Moreover, the evolution of tissue-specific regulation of minor spliceosome components, MIG expression, and alternative splicing of MIGs could drive the scaling of specific features/tissues during multicellular evolution (Olthof et al., 2019). Both whole-organism and tissue scaling have been integral to multicellular evolution, in which changes in body plan and/or specific tissue sizes allowed exploitation of novel niches, new adaptations to climate, avoidance of predators, or access to prey. We also present evidence of the link between minor splicing and tissue scaling in the multiple MIGs affected in animal domestication syndrome, in turn suggesting that suppression of minor splicing or MIG expression in the neural crest cell population allowed for decreased craniofacial and nervous tissue size during animal domestication. Our meta-analysis also suggests that minor splicing and MIGs may represent exciting new targets in crop plant domestication, where specific plant tissues, such as the seed and fruit, are hypermorphic (Doebley et al., 2006). These connections to new candidate genes in animal and crop domestication syndromes will be particularly relevant for researchers working in agriculture, crop optimization, and evolution of domestication research.

The high degree of minor intron conservation in plant and animal lineages is striking, and we expect that the minor spliceosome serves as a regulatory switch for numerous features unique to these specific lineages of multicellular life, in addition to its general role in controlling organism and tissue scaling. For example, in humans, all VGSC and VGCC alpha subunit genes contain minor introns, implicating proper minor splicing in neuronal function. Therefore, regulation of minor splicing may be a potent controller of neuron excitability, action potential propagation, and synaptic vesicle release; thus, in animal evolution, the minor splicing pathway may have been used to fine-tune neuronal activity. Given the rapid pace of research into the role of minor splicing in development and disease, we predict there will be a sharp increase in the identification of animal- and plant-specific regulatory pathways involving either MIGs or minor splicing. These newly identified, lineage-specific pathways, all linked by minor spliceosome activity, will provide new nodes of research for clinicians, botanists, evolutionary biologists, and agriculture scientists to pursue.

## Data Availability Statement

Publicly available datasets were analyzed in this study. This data can be found here: https://figshare.com/articles/Broad_Institute_Cancer_Dependency_Map_CRISPR_Avana_dataset_17Q4/5520160/1, https://journals.plos.org/plosone/article?id=10.1371/journal.pone.0185306. The term SRP034606 refers to the SRA for the RNAseq dataset produced by Singh et al., 2013. The GEO database link for this dataset is: https://www.ncbi.nlm.nih.gov/geo/query/acc.cgi?acc=GSE53481.

## Author Contributions

MB and KD extracted and analyzed the published essentialome (MB), cell cycle RNAseq (KD), and candidate animal domestication gene datasets (KD). MB and KD also generated figures and supplementary tables. MB, KD, and RK wrote and edited the manuscript, which was conceptualized by RK.

## Funding

This work was supported by grants from the National Institute of Neurological Disorders and Stroke (#1R21NS096684-01A1 and #5R01NS102538-02) and the University of Connecticut (internal) to RK. In addition, this material is based upon work supported by the National Science Foundation under Grant No. 2018257410 to KD.

## Conflict of Interest

The authors declare that the research was conducted in the absence of any commercial or financial relationships that could be construed as a potential conflict of interest.
