# Supplementary material for: An Integrated Model of Minor Intron Emergence and Conservation
Source: Front Genet. 2019 Nov 13;10:1113. doi: 10.3389/fgene.2019.01113 (PMC6865273; doi:10.3389/fgene.2019.01113)
Supplement: Supplementary file 2 [file Table_2.docx]

**Supplementary Table 2. Mammalian functions of the 59 younger MIGs.** Columns three through five indicate whether that MIG is found in the total essentialome, the majority (Maj.) essentialome, or the core essentialome, respectively; Y=yes, N=no. Column six lists the oldest clade that each MIG can be traced to, according to eggNOG v5. Based on the findings of literature curation, listed in the Function Description column, MIGs were classified into different functional categories (eighth column). Syn=synonyms; Essent.=essentialomes; CNS=central nervous system.

| **MIG** | **Syn** | **Total Essent.?** | **Maj.**  **Essent.?** | **Core**  **Essent.?** | **Oldest Clade** | **Function Description** | **Functional Categories** |
| --- | --- | --- | --- | --- | --- | --- | --- |
| *HSBP1L1* |  | N | N | N | Amniota | mammalian function unknown | mammalian function unknown |
| *COA3* | *CCDC56, COX25, HSPC009, MITRAC12* | Y | N | N | Bilateria | a mitochondrial transmembrane protein that functions as a core component of the MITRAC complex, which regulates the assembly of cytochrome c oxidase (COX, complex IV), the final enzyme of the electron transport chain (Mick et al., 2012; Clemente et al., 2013); also regulates translation of COX1, a central subunit of complex IV (Mick et al. 2012; Clemente et al., 2013) | metabolism (cellular respiration) |
| *CRTC1* | *MAML2, MECT1, TORC1* | N | N | N | Bilateria | coactivator of the transcription factor CREB (Conkright et al., 2003); also modulates activity of the transcription factor complex AP-1, which promotes cellular proliferation (Canettieri et al., 2009); nuclear translocation is induced by coincident calcium and cAMP signaling, linking calcium and cAMP levels to CREB activity regulation (Kovacs et al., 2007); via role in linking coincident calcium and cAMP signaling to CREB activity, regulates long-term potentiation in the hippocampus and informs memory stabilization (Kovacs et al., 2007; Sekeres et al., 2012; Parra-Damas et al., 2017); similarly functions in the amygdala to regulate long-term fear memory (Nonaka et al., 2014); via role in CREB activity, involved in circadian clock regulation (Jagannath et al., 2013); in mice, genetic trapping results in energy imbalance and obesity, likely by disrupting leptin signaling (Altarejos et al., 2008… | transcription regulation; neuron function; energy balance; liver function |
| **MIG** | **Syn** | **Total Essent.?** | **Maj.**  **Essent.?** | **Core**  **Essent.?** | **Oldest Clade** | **Function Description** | **Functional Categories** |
| *CRTC1* | *MAML2, MECT1, TORC1* | N | N | N | Bilateria | … Breuillaud et al., 2009); via role in linking cAMP levels to NCoR corepressor activity, prevents expression of lipogenic genes in the liver (Kim, 2016) | transcription regulation; neuron function; energy balance; liver function |
| *CRTC2* | *TORC2* | Y | N | N | Bilateria | coactivator of the transcription factor CREB (Conkright et al., 2003); nuclear translocation is induced by coincident calcium and cAMP signaling, linking calcium and cAMP levels to CREB activity regulation (Conkright et al., 2003); nuclear translocation is triggered by ER stress, followed by interaction with the transcription factor ATF6, increasing expression of ER stress response genes (Wang et al., 2009); in the liver, links both chronic hyperglycemia and glucagon to increased CREB activity, resulting in upregulation of gluconeogenesis genes (Koo et al., 2005; Dentin et al., 2008); in contrast, insulin triggers localization of CRTC2 to the cytoplasm, suppressing expression of gluconeogenesis genes (Dentin et al., 2007); in the hypothalamus, hyperglycemia triggers nuclear localization and increased CREB activity at genes in the energy-sensing pathway, while fasting does not (Lerner et al., 2009); via role in linking coincident calcium and cAMP signaling to CREB activity, regulates response to glucose and gut hormones in pancreatic cells (Screaton et al., 2004; Jansson et al., 2008); prostaglandin E2-mediated CRTC2 nuclear localization, and subsequently increased CREB activity, modulates cytokine expression, drives differentiation in helper T 17 (Th17)… | transcription regulation; glucose regulation; energy balance; immune response; endoplasmic reticulum integrity |
| **MIG** | **Syn** | **Total Essent.?** | **Maj.**  **Essent.?** | **Core**  **Essent.?** | **Oldest Clade** | **Function Description** | **Functional Categories** |
| *CRTC2* | *TORC2* | Y | N | N | Bilateria | cells, and regulates bone marrow hematopoiesis (Hernandez et al., 2015; Kim et al., 2017) | transcription regulation; glucose regulation; energy balance; immune response; endoplasmic reticulum integrity |
| *CRTC3* | *TORC3* | Y | N | N | Bilateria | coactivator of the transcription factor CREB (Conkright et al., 2003); leptin and beta-adrenergic receptor agonists increase nuclear accumulation of CRTC3 in adipose tissue, inducing CREB-mediated transcription of RGS2, a cAMP pathway inhibitor (Song et al., 2010); in brown adipose tissue, CRTC3 interacts with the transcription factor C/EBPbeta to control the expression of brown adipose tissue differentiation and thermogenesis genes (Shan et al., 2016; Yoon et al., 2018); knockout mice display increased energy expenditure and body temperature, tied to increased lipolysis in white and brown adipose tissue (Song et al., 2010); in macrophages, regulates expression of anti-inflammatory cyto-kines linked to regulatory macrophage phenotype, via role as CREB coactivator (Clark et al., 2012) | transcription regulation; energy balance; immune response |
| *FAM178A* | *SLF2, C10orf6* | N | N | N | Bilateria | complexes with RAD18 to recruit the SMC5/6 cohesion complex to DNA double-strand breaks, which relaxes the surrounding chromatin to allow for further recruitment of DNA damage response proteins (Raschle et al., 2015); knockdown results in mitotic defects (Raschle et al., 2015) | genome integrity (DNA damage); cell cycle (mitosis) |
| **MIG** | **Syn** | **Total Essent.?** | **Maj.**  **Essent.?** | **Core**  **Essent.?** | **Oldest Clade** | **Function Description** | **Functional Categories** |
| *KIAA1377* | *CEP126* | N | N | N | Bilateria | a ~126 kDa centrosomal protein, which localizes to the centrosome and pericentriolar satellites during interphase, the centrosome and spindle during mitosis, and the midbody during cytokinesis (Chen et al., 2009; Tipton et al., 2012; Bonavita et al., 2014); knockdown in cultured cells results in mislocalization of the pericentriolar satellites, impaired microtubule organization, defects in primary cilium formation, dis-organization of the mitotic spindle, and cytokinesis defects (Chen et al., 2009; Bonavita et al., 2014) | cytoskeleton organization (centrosome); cell cycle (mitosis, cytokinesis); ciliogenesis |
| *PROX1* |  | Y | N | N | Bilateria | prospero homeobox transcription factor 1; regulates numerous aspects of embryonic development, including development of the CNS, lens, endothelial lymphatic system, liver, pancreas, and heart, by controlling cell proliferation/ differentiation, cell fate, migration, and sarcomere protein localization (reviewed by Elsir et al., 2012 and Stergiopoulos et al., 2014) | transcription regulation; cell cycle (proliferation, differentiation), cell migration, development (multiple organs) |
| *PROX2* |  | N | N | N | Bilateria | prospero homeobox transcription factor 2; knockout mice are born at expected Mendelian ratios and appear phenotypically normal (Nishijima and Ohtoshi, 2006) | transcription regulation |
| *SELM* | *SELENOMSEPM* | N | N | N | Bilateria | selenoprotein M, a selenoprotein localized to the endoplasmic reticulum (Korotkov et al., 2002); protective against oxidative stress (Reeves et al., 2010); knockout mice display adult-onset obesity with increased adoposity (Pitts et al., 2013) | stress response (oxidative stress); energy balance |
| *SPTSSA* | *C14orf147, SSSPTA* | Y | N | N | Bilateria | a small subunit of the serine palmitoyltransferase complex, which catalyzes the first step of sphingolipid biosynthesis (Han et al., 2009); based on knockdown studies, involved in fatty acid… | lipid metabolism |
| **MIG** | **Syn** | **Total Essent.?** | **Maj.**  **Essent.?** | **Core**  **Essent.?** | **Oldest Clade** | **Function Description** | **Functional Categories** |
| *SPTSSA* | *C14orf147, SSSPTA* | Y | N | N | Bilateria | remodeling of phosphatidylinositol, possibly by controlling LPIAT1 localization to the mitochon-dria-associated membrane (Hirata et al., 2013) | lipid metabolism |
| *C11orf85* | *MAJIN* | N | N | N | Chordata | a transmembrane protein found in the inner nuclear membrane of meiotic cells, which complexes with TERB1 and TERB2 to bind telomeres to the inner nuclear membrane during early meiosis, in order to pair homologous chromosomes together (Shibuya et al., 2015; Dunce et al., 2018; Wang et al., 2019) | cell cycle (meiosis) |
| *C16orf59* | *TEDC2* | Y | N | N | Chordata | mammalian function unknown | mammalian function unknown |
| *SH3TC2* | *CMT4C, MNMN* | N | N | N | Chordata | an effector protein of Rab11, which regulates endosome recycling, in Schwann cells, where it localizes to the perinuclear endocytic recycling compartment (Arnaud et al., 2009; Roberts et al., 2010; Stendel et al., 2010); also informs the internalization of ErbB2 in Schwann cells, an important step in myelination regulation (Gouttenoire et al., 2013); knockout in mice causes peripheral nerve hypomyelination, widening of the nodes of Ranvier, and reduced conduction velocity (Arnaud et al., 2009) | transport (endosomal); neuron function (myelination) |
| *ICE1* | *KIAA0947* | Y | N | N | Deuterostomia | a scaffold subunit of the little elongation complex (LEC), which functions in RNA polymerase II-driven snRNA transcription (Smith et al., 2011); ICE1 is specifically required for LEC recruitment and the initiation step of snRNA transcription (Hu et al., 2013); also functions as a peripheral exon junction complex (EJC) factor to enhance EJC-dependent nonsense-mediated decay (Baird et al., 2018) | non-coding RNA biogenesis (snRNA); RNA processing (nonsense-mediated decay) |
| **MIG** | **Syn** | **Total Essent.?** | **Maj.**  **Essent.?** | **Core**  **Essent.?** | **Oldest Clade** | **Function Description** | **Functional Categories** |
| *VEZT* | *VEZATIN* | Y | N | N | Deuterostomia | a transmembrane protein component of adherens cell junctions, which interacts with myosin VIIA and cadherin-catenin complexes (Kussel-Andermann et al., 2000); knockdown in pre-implantation mouse embryos results in developmental arrest, likely through disruption of cell-cell contacts (Hyenne et al., 2005); similarly, knockout in mice results in pre-implantation embryonic lethality, due to disruption of adherens junctions (Hyenne et al., 2007); in adult hippocampal neurons, localizes to dendritic spines and regulates dendritic spine shape/maturation, which in turn affects anxiety and memory formation (Danglot et al., 2012) | cell adhesion; neuron function (spine morphogenesis) |
| *ALS2CR11* | *C2CD6* | N | N | N | Eumetazoa | mammalian function unknown | mammalian function unknown |
| *C14orf93* | *RTFC* | N | N | N | Eumetazoa | based on an *in vitro* thyroid differentiation assay in mouse embryonic stem cells, enhances thyroid differentiation (Yu et al., 2017); *C14orf93* knockout mice are phenotypically normal, while females display mildly impaired thyroid function (Yu et al., 2017) | energy balance; thyroid function |
| *C1orf109* |  | Y | Y | N | Eumetazoa | functions in cancer cell proliferation, based on misexpression and knockdown studies (Liu et al., 2012) | cell cycle (proliferation) |
| *C3orf17* | *NEPRO, NET17* | Y | Y | N | Eumetazoa | involved in maintenance of neocortex neural progenitor cells downstream of Notch and plays a role in repression of proneural gene expression; misexpression causes inhibition of neuronal differentiation in the early neocortex, while knock-down drives neuron differentiation (Muroyama and Saito, 2009); localized to the nucleolus, and… | cell cycle (proliferation/  progenitor cell identity) |
| **MIG** | **Syn** | **Total Essent.?** | **Maj.**  **Essent.?** | **Core**  **Essent.?** | **Oldest Clade** | **Function Description** | **Functional Categories** |
| *C3orf17* | *NEPRO, NET17* | Y | Y | N | Eumetazoa | … knockout in mice causes impaired blastocyst formation and apoptosis (Hashimoto et al., 2015) | cell cycle (proliferation/  progenitor cell identity) |
| *C5orf34* |  | N | N | N | Eumetazoa | functions in cancer cell proliferation and migration, based on knockdown studies (He et al., 2019) | cell cycle (proliferation); cell migration |
| *DCTN3* | *DCTN22* | Y | N | N | Eumetazoa | a dynactin light chain subunit necessary for mitosis progression and chromosome segregation (Ozaki et al., 2011; Fan et al., 2015) | cell cycle (mitosis) |
| *MEI1* | *HYDM3, SPATA38* | N | N | N | Eumetazoa | evidence indicates a role in initiation of meiotic recombination (Reinholdt and Schimenti, 2005; Liebe et al., 2006); in mice, mutation results in sterility, due to impaired meiotic chromosome synapsis and meiotic prophase arrest in spermatocytes and defects in metaphase I in oocytes (Libby et al., 2002) | cell cycle (meiosis); fertility |
| *MTBP* | *MDM2BP* | Y | Y | Y | Eumetazoa | an MDM2-binding protein that enhances MDM2-mediated p53 degradation (Boyd et al., 2000;Brady et al., 2005); knockdown reduces MAD1 and MAD2 kinetochore localization and triggers chromosome missegregation (Agarwal et al., 2011); via interaction with the DNA replication factor TICRR, regulates DNA replication initiation (Boos et al., 2013) | cell cycle (DNA replication/S phase, mitosis) |
| *PALLD* | *CGI151, MYN, PNCA1, SIH002* | N | N | N | Eumetazoa | a cytoskeleton-associated protein that regulates actin and microtubule organization (Goicoechea et al., 2008;Sun et al., 2017;Zhang et al., 2017); via its role in actin organization, regulates cell motility and phagocytosis (Goicoechea et al., 2008;Nguyen et al., 2014); via its role in microtubule organization, mediates mitotic spindle… | cytoskeleton organization (actin, microtubules); cell migration; cell cycle (mitosis)… |
| **MIG** | **Syn** | **Total Essent.?** | **Maj.**  **Essent.?** | **Core**  **Essent.?** | **Oldest Clade** | **Function Description** | **Functional Categories** |
| *PALLD* | *CGI151, MYN, PNCA1, SIH002* | N | N | N | Eumetazoa | orientation (Zhang et al., 2017); knockout in mice results in embryonic lethality and neurulation defects, tied to cell adherence and migration defects (Luo et al., 2005); knockdown in myoblasts affects muscle differentiation, indicating a role in myogenesis (Nyugen et al., 2014) | cytoskeleton organization (actin, microtubules); cell migration; cell cycle (mitosis); development (CNS, muscle) |
| *SPC24* | *SPBC24* | Y | Y | Y | Eumetazoa | NDC80 kinetochore complex component; required to establish and maintain kinetochore-microtubule attachment in mitosis (McCleland et al., 2004) | cell cycle (mitosis) |
| *TAF1C* | *SL1, TAFI110, TAFI95* | Y | Y | N | Eumetazoa | TATA box-binding protein associated factor (TAF) for RNA polymerase I; part of SL1 complex, which directs RNA polymerase I transcription and can independently interaction with rDNA promoters (Friedrich et al., 2005) | non-coding RNA biogenesis (rRNA) |
| *WLS* | *GPR177, EVI, C1orf139, MRP* | N | N | N | Eumetazoa | a transmembrane protein essential for Wnt secretion, due to its role in trafficking Wnts from the endoplasmic reticulum to the plasma membrane (Banziger et al., 2006; Bartscherer et al., 2006; Najdi et al., 2012; Yu et al., 2014); in mice, knockout results in early embryonic lethality due to disrupted patterning of the anterior-posterior axis (Fu et al., 2009) | transport (endosomal); protein secretion; development (patterning) |
| *C11orf80* | *HYDM4, TOP6BL, TOPOVIBL* | N | N | N | Euteleostomi | an interactor of SPO11, which together initiate meiotic recombination (Robert et al., 2016) | cell cycle (meiosis); fertility |
| *C14orf39* | *Six6os1* | N | N | N | Euteleostomi | a subunit of the central element, itself part of the synaptonemal complex, which is required for chromosome synapsis in meiotic prophase I (Gomez et al., 2016) | cell cycle (meiosis); fertility) |
| **MIG** | **Syn** | **Total Essent.?** | **Maj.**  **Essent.?** | **Core**  **Essent.?** | **Oldest Clade** | **Function Description** | **Functional Categories** |
| *CDCA2* | *PPP1R81, Repo-Man* | N | N | N | Euteleostomi | a targeting protein for serine/threonine phosphatase PP1 (Trinkle-Mulcahy et al., 2006); through its targeting function, regulates chromatin architecture/condensation during interphase and mitosis, Aurora B localization during mitotic spindle assembly, chromatin-localized ATM activation in the DNA damage response, and coordination of chromosome organization and nuclear envelope organization post-mitosis (Vagnarelli et al., 2006; Peng et al., 2010; Qian et al., 2011; Vagnarelli et al., 2011; Vagnarelli and Earnshaw, 2012; Wurzenberger et al., 2012) | chromatin organization; cell cycle (mitosis, nuclear envelope reformation); genome integrity (DNA damage) |
| *CNST* | *C1orf71, PPP1R64* | N | N | N | Euteleostomi | consortin, a transmembrane protein of the *trans*-Golgi network that interacts with Golgi clathrin adaptors to traffic transmembrane proteins to the plasma membrane (del Castillo et al., 2010); via its role in regulating connexin trafficking to the plasma membrane, controls cell adhesion (del Castillo et al., 2010) | transport (transmembrane protein); cell adhesion |
| *TCTN3* | *C10orf61, JBTS18, OFD4, TECT3* | N | N | N | Euteleostomi | a transmembrane protein member of the tectonic family, which is localized to the transition zone of primary cilia and functions in Hedgehog signaling transduction (Thomas et al., 2012); is required for the proper localization of ciliary membrane proteins (Wang et al., 2017); knockout in mice causes embryonic lethality, with disrupted Hedgehog-induced patterning and impaired ciliogenesis (Wang et al., 2017; Wang et al., 2018a) | ciliogenesis; development (patterning) |
| *TMEM44* |  | N | N | N | Euteleostomi | a predicted transmembrane protein whose mammalian function is poorly understood (Moyer et al., 2009) | mammalian function unknown |
| **MIG** | **Syn** | **Total Essent.?** | **Maj.**  **Essent.?** | **Core**  **Essent.?** | **Oldest Clade** | **Function Description** | **Functional Categories** |
| *BCAR1* | *CAS, CAS1, CASS1, CRKAS* | Y | N | N | Metazoa | a member of the Cas family of adaptor protein that has numerous signal transduction functions, including proliferation, cytoskeleton organization, cell migration, cell adhesion, mechano-transduction, axon guidance, myogenic differentiation, and bone resorption (reviewed by Tikhmyanova et al., 2010 and Shahmoradi et al., 2015); knockout in mice causes embryonic lethality and widespread cardiovascular defects (Honda et al., 1998) | signal transduction; cytoskeleton organization; cell migration; cell cycle (proliferation, differentiation); cell adhesion; development (cardiovascular, muscle, CNS); bone function |
| *C18orf63* | *DKFZP781G0119* | N | N | N | Metazoa | mammalian function unknown | mammalian function unknown |
| *CCDC28A* | *C6orf80, CCRL1AP* | N | N | N | Metazoa | mammalian function unknown | mammalian function unknown |
| *CNEP1R1* | *C16orf69, NEP1R1, TMEM188* | Y | N | N | Metazoa | the regulatory subunit of a complex formed with CTDNEP1, which together dephosphorylate lipins, thereby regulating lipid metabolism and diacylglycerol (DAG) formation (Han et al., 2012) | lipid metabolism; signal transduction |
| *CUEDC1* |  | N | N | N | Metazoa | mammalian function unknown | mammalian function unknown |
| *ERICH6* | *C3orf44A, FAM194A* | N | N | N | Metazoa | mammalian function unknown | mammalian function unknown |
| *ERICH6B* | *FAM194B* | N | N | N | Metazoa | mammalian function unknown | mammalian function unknown |
| **MIG** | **Syn** | **Total Essent.?** | **Maj.**  **Essent.?** | **Core**  **Essent.?** | **Oldest Clade** | **Function Description** | **Functional Categories** |
| *INO80D* | *FLJ20309* | N | N | N | Metazoa | a subunit of the INO80 complex that is dispensable for this complex's nucleosome sliding and DNA-dependent ATPase activity, based on cell culture and *in vitro* work, but is suggested to play a role in INO80-transcription factor interactions (Chen et al., 2011) | transcription regulation |
| *INTS10* | *C8orf35, INT10* | Y | N | N | Metazoa | a member of the integrator complex, which associates with RNA polymerase II and mediates 3' end processing of snRNAs (Baillat et al., 2005) | non-coding RNA biogenesis (snRNA) |
| *NOL11* |  | Y | N | N | Metazoa | a component of the human ribosomal small subunit processome, whose knockdown results in defects in cleavage steps required to generate mature 18S rRNA and in rDNA transcription (Freed et al., 2012) | non-coding RNA biogenesis (rRNA) |
| *TTC23* |  | N | N | N | Metazoa | a protein component of the Ellis-van-Creveld zone of the primary cilium; CRISPR-mediated disruption impairs Hedgehog signaling transduction (Breslow et al., 2018) | signal transduction |
| *TTC23L* |  | N | N | N | Metazoa | mammalian function unknown | mammalian function unknown |
| *AHCTF1* | *ELYS, MST108* | Y | Y | N | Opisthokonta | in HeLa cells, localizes to nuclear pore and kinetochores, and knockdown causes cytokinesis defects (Rasala et al., 2006); required for nuclear pore assembly (Rasala et al., 2006); siRNA-mediated knockdown in HeLa cells results in mislocalization of LBR, which is important for reforming the nuclear envelope post-mitosis (Clever et al., 2012); mouse knockout results in early embryonic lethality (Okita et al., 2004) | cell cycle (mitosis, cytokinesis); transport (nucleo-cytoplasmic) |
| **MIG** | **Syn** | **Total Essent.?** | **Maj.**  **Essent.?** | **Core**  **Essent.?** | **Oldest Clade** | **Function Description** | **Functional Categories** |
| *ARRB1* | *ARB1, ARR1* | Y | N | N | Opisthokonta | β-arrestin 1, an adaptor protein for G protein-coupled receptors (GPCRs) that mediates GPCR desensitization, clathrin-mediated trafficking of GPCRs and various other receptors, receptor ubiquitination, and arrestin-based signaling (reviewed by Tian et al., 2014 and Ranjan et al., 2017); in the nucleus, regulates transcription (Kang et al., 2005; reviewed by Rosano and Bagnato, 2016); due to its roles in many signal transduction pathways and its association with various transcription factors, ARRB1 is linked to numerous biological processes, such as immune system activation, cell proliferation and survival, adipogenesis and thermogenesis, photo-transduction, and stress response (reviewed by Peterson and Luttrell, 2017; also see Wang et al., 2006; Nikonov et al., 2008; Hara et al., 2011; Yang et al., 2012; Rosano and Bagnato, 2016; Wang et al., 2016) | signal transduction; transcription regulation; transport (transmembrane protein); cell cycle (proliferation); energy balance; immune response; neuron function |
| *C17orf75* | *SRI2* | Y | N | N | Opisthokonta | forms a complex with WDR11 and FAM91A1, but appears to be dispensable for proper complex localization to the trans-Golgi network (Navarro Negredo et al., 2018); it is not currently known if C17orf75 is necessary for this complex's vesicle tethering activity (Navarro Negredo et al., 2018) | mammalian function unknown |
| *C3orf20* |  | N | N | N | Opisthokonta | mammalian function unknown | mammalian function unknown |
| *CCDC134* |  | N | N | N | Opisthokonta | in its secreted form, promotes T-cell activation, proliferation, and cytotoxic function (Huang et al., 2014); when nuclear, binds with ADA2 in the p300/CBP-associated factor (PCAF) complex, which functions in chromosome remodeling… | transcription regulation; genome integrity (DNA damage)… |
| **MIG** | **Syn** | **Total Essent.?** | **Maj.**  **Essent.?** | **Core**  **Essent.?** | **Oldest Clade** | **Function Description** | **Functional Categories** |
| *CCDC134* |  | N | N | N | Opisthokonta | … and transcription factor coactivation (Huang et al., 2012); knockdown studies also implicate CCDC134 in the DNA damage response (Huang et al., 2012); knockout in mice is embryonically lethal, with impaired brain development, cardiovascular defects, and liver hypoplasia (Yu et al., 2018) | transcription regulation; genome integrity (DNA damage); immune response; development (CNS, liver, cardiovascular) |
| *CCDC43* |  | Y | N | N | Opisthokonta | mammalian function unknown | mammalian function unknown |
| *CYBA* | *p22-PHOX* | N | N | N | Opisthokonta | a subunit of most NADPH oxidases (NOXs), a source of superoxides in the cell, that is important for the stabilization of these complexes (Stasia, 2016); with NOX2, forms cytochrome *b*_558_, which phagocytes utilize to kill pathogenic microorganisms (reviewed by Stasia, 2016); with NOX3, forms a separate cytochrome required for vestibular development in mice (Nakano et al., 2008) | superoxide generation; immune response; development (inner ear) |
| *KIAA1524* | *CIP2A* | Y | N | N | Opisthokonta | an oncoprotein that inhibits protein phosphatase 2A and enhances proliferation of both neural progenitor cell and spermatogonial progenitor cells (Kerosuo et al., 2010;Ventela et al., 2012); via interaction with NEK2, regulates centrosome separation and mitotic spindles during mitosis (Jeong et al., 2014); knockdown experiments indicate roles in primary cilia disassembly and cell metabolism (Jeong et al., 2018) | cell cycle (proliferation); ciliogenesis; metabolism (cellular) |
| *NOL8* | *C9orf34, NOP132* | Y | N | N | Opisthokonta | associates primarily with proteins involved in ribosome biogenesis and RNA metabolism, such as the helicase DDX47, which functions in… | non-coding RNA biogenesis (rRNA) |
| **MIG** | **Syn** | **Total Essent.?** | **Maj.**  **Essent.?** | **Core**  **Essent.?** | **Oldest Clade** | **Function Description** | **Functional Categories** |
| *NOL8* | *C9orf34, NOP132* | Y | N | N | Opisthokonta | … rRNA processing (Sekiguchi et al., 2006); knockdown results in improper localization of DDX47, implicating NOL8 in the recruitment of pre-rRNA processing proteins (Sekiguchi et al., 2006) | non-coding RNA biogenesis (rRNA) |
| *RNF220* | *C1orf164* | N | N | N | Opisthokonta | an E3 ubiquitin ligase known to target Sin3B, which functions in transcription regulation, and the transcription factors DBX1/2 and NKX2.2 for degradation, while it functions in the stabilization of beta-catenin, a key component of the Wnt signaling pathway (Kong et al., 2010; Ma et al., 2014; Kim et al., 2018); knockout in mice disrupts patterning of the developing spinal cord alongside impaired neuron production, implicating RNF220 in cell differentiation/cell cycle exit (Kim et al., 2018) | protein degradation (ubiquitination); transcription regulation; development (patterning, CNS); cell cycle (exit/ differentiation) |
| *SFI1* | *PISD, PPP1R139* | N | N | N | Opisthokonta | a centrosomal protein that binds with centrin (Kilmartin, 2003); interacts with serine/threonine phosphatase 1 (PP1) (Hendrickx et al., 2009); mammalian function unclear | mammalian function unknown |
| *TBC1D32* | *BROMI, C6orf170, C6orf171* | N | N | N | Opisthokonta | also known as Broad-minded, which connects ciliogenesis/cilium morphology and Hedgehog signaling (Ko et al., 2010; Wang et al., 2018b); knockout in mice causes embryonic lethality and exencephaly, alongside defects in cilia morphology (Wang et al., 2018b) | ciliogenesis; development (patterning) |
| *WDR41* | *MSTP048* | N | N | N | Opisthokonta | a Golgi-associated protein that regulates autophagy, by mediating GEF activity targeting RAB8A and RAB8B (Sellier et al., 2016; Sullivan et al., 2016) | autophagy |

**Supplementary References**

Agarwal, N., Tochigi, Y., Adhikari, A.S., Cui, S., Cui, Y., and Iwakuma, T. (2011). MTBP plays a crucial role in mitotic progression and chromosome segregation. *Cell Death Differ* 18**,** 1208-1219.

Altarejos, J.Y., Goebel, N., Conkright, M.D., Inoue, H., Xie, J., Arias, C.M., Sawchenko, P.E., and Montminy, M. (2008). The Creb1 coactivator Crtc1 is required for energy balance and fertility. *Nat Med* 14**,** 1112-1117.

Arnaud, E., Zenker, J., De Preux Charles, A.S., Stendel, C., Roos, A., Medard, J.J., Tricaud, N., Kleine, H., Luscher, B., Weis, J., Suter, U., Senderek, J., and Chrast, R. (2009). SH3TC2/KIAA1985 protein is required for proper myelination and the integrity of the node of Ranvier in the peripheral nervous system. *Proc Natl Acad Sci U S A* 106**,** 17528-17533.

Baillat, D., Hakimi, M.A., Naar, A.M., Shilatifard, A., Cooch, N., and Shiekhattar, R. (2005). Integrator, a multiprotein mediator of small nuclear RNA processing, associates with the C-terminal repeat of RNA polymerase II. *Cell* 123**,** 265-276.

Baird, T.D., Cheng, K.C., Chen, Y.C., Buehler, E., Martin, S.E., Inglese, J., and Hogg, J.R. (2018). ICE1 promotes the link between splicing and nonsense-mediated mRNA decay. *Elife (Cambridge)* 7.

Banziger, C., Soldini, D., Schutt, C., Zipperlen, P., Hausmann, G., and Basler, K. (2006). Wntless, a conserved membrane protein dedicated to the secretion of Wnt proteins from signaling cells. *Cell* 125**,** 509-522.

Bartscherer, K., Pelte, N., Ingelfinger, D., and Boutros, M. (2006). Secretion of Wnt ligands requires Evi, a conserved transmembrane protein. *Cell* 125**,** 523-533.

Bonavita, R., Walas, D., Brown, A.K., Luini, A., Stephens, D.J., and Colanzi, A. (2014). Cep126 is required for pericentriolar satellite localisation to the centrosome and for primary cilium formation. *Biol Cell* 106**,** 254-267.

Boos, D., Yekezare, M., and Diffley, J.F. (2013). Identification of a heteromeric complex that promotes DNA replication origin firing in human cells. *Science* 340**,** 981-984.

Boyd, M.T., Vlatkovic, N., and Haines, D.S. (2000). A novel cellular protein (MTBP) binds to MDM2 and induces a G1 arrest that is suppressed by MDM2. *J Biol Chem* 275**,** 31883-31890.

Brady, M., Vlatkovic, N., and Boyd, M.T. (2005). Regulation of p53 and MDM2 activity by MTBP. *Mol Cell Biol* 25**,** 545-553.

Breslow, D.K., Hoogendoorn, S., Kopp, A.R., Morgens, D.W., Vu, B.K., Kennedy, M.C., Han, K., Li, A., Hess, G.T., Bassik, M.C., Chen, J.K., and Nachury, M.V. (2018). A CRISPR-based screen for Hedgehog signaling provides insights into ciliary function and ciliopathies. *Nat Genet* 50**,** 460-471.

Breuillaud, L., Halfon, O., Magistretti, P.J., Pralong, F.P., and Cardinaux, J.R. (2009). Mouse fertility is not dependent on the CREB coactivator Crtc1. *Nat Med* 15**,** 989-990; author reply 991.

Canettieri, G., Coni, S., Della Guardia, M., Nocerino, V., Antonucci, L., Di Magno, L., Screaton, R., Screpanti, I., Giannini, G., and Gulino, A. (2009). The coactivator CRTC1 promotes cell proliferation and transformation via AP-1. *Proc Natl Acad Sci U S A* 106**,** 1445-1450.

Chen, L., Cai, Y., Jin, J., Florens, L., Swanson, S.K., Washburn, M.P., Conaway, J.W., and Conaway, R.C. (2011). Subunit organization of the human INO80 chromatin remodeling complex: an evolutionarily conserved core complex catalyzes ATP-dependent nucleosome remodeling. *J Biol Chem* 286**,** 11283-11289.

Chen, T.C., Lee, S.A., Hong, T.M., Shih, J.Y., Lai, J.M., Chiou, H.Y., Yang, S.C., Chan, C.H., Kao, C.Y., Yang, P.C., and Huang, C.Y. (2009). From midbody protein-protein interaction network construction to novel regulators in cytokinesis. *J Proteome Res* 8**,** 4943-4953.

Clark, K., Mackenzie, K.F., Petkevicius, K., Kristariyanto, Y., Zhang, J., Choi, H.G., Peggie, M., Plater, L., Pedrioli, P.G., Mciver, E., Gray, N.S., Arthur, J.S., and Cohen, P. (2012). Phosphorylation of CRTC3 by the salt-inducible kinases controls the interconversion of classically activated and regulatory macrophages. *Proc Natl Acad Sci U S A* 109**,** 16986-16991.

Clemente, P., Peralta, S., Cruz-Bermudez, A., Echevarria, L., Fontanesi, F., Barrientos, A., Fernandez-Moreno, M.A., and Garesse, R. (2013). hCOA3 stabilizes cytochrome c oxidase 1 (COX1) and promotes cytochrome c oxidase assembly in human mitochondria. *J Biol Chem* 288**,** 8321-8331.

Clever, M., Funakoshi, T., Mimura, Y., Takagi, M., and Imamoto, N. (2012). The nucleoporin ELYS/Mel28 regulates nuclear envelope subdomain formation in HeLa cells. *Nucleus* 3**,** 187-199.

Conkright, M.D., Canettieri, G., Screaton, R., Guzman, E., Miraglia, L., Hogenesch, J.B., and Montminy, M. (2003). TORCs: transducers of regulated CREB activity. *Mol Cell* 12**,** 413-423.

Danglot, L., Freret, T., Le Roux, N., Narboux Neme, N., Burgo, A., Hyenne, V., Roumier, A., Contremoulins, V., Dauphin, F., Bizot, J.C., Vodjdani, G., Gaspar, P., Boulouard, M., Poncer, J.C., Galli, T., and Simmler, M.C. (2012). Vezatin is essential for dendritic spine morphogenesis and functional synaptic maturation. *J Neurosci* 32**,** 9007-9022.

Del Castillo, F.J., Cohen-Salmon, M., Charollais, A., Caille, D., Lampe, P.D., Chavrier, P., Meda, P., and Petit, C. (2010). Consortin, a trans-Golgi network cargo receptor for the plasma membrane targeting and recycling of connexins. *Hum Mol Genet* 19**,** 262-275.

Dentin, R., Hedrick, S., Xie, J., Yates, J., 3rd, and Montminy, M. (2008). Hepatic glucose sensing via the CREB coactivator CRTC2. *Science* 319**,** 1402-1405.

Dentin, R., Liu, Y., Koo, S.H., Hedrick, S., Vargas, T., Heredia, J., Yates, J., 3rd, and Montminy, M. (2007). Insulin modulates gluconeogenesis by inhibition of the coactivator TORC2. *Nature* 449**,** 366-369.

Dunce, J.M., Milburn, A.E., Gurusaran, M., Da Cruz, I., Sen, L.T., Benavente, R., and Davies, O.R. (2018). Structural basis of meiotic telomere attachment to the nuclear envelope by MAJIN-TERB2-TERB1. *Nat Commun* 9**,** 5355.

Elsir, T., Smits, A., Lindstrom, M.S., and Nister, M. (2012). Transcription factor PROX1: its role in development and cancer. *Cancer Metastasis Rev* 31**,** 793-805.

Fan, Y., Zhao, H.C., Liu, J., Tan, T., Ding, T., Li, R., Zhao, Y., Yan, J., Sun, X., Yu, Y., and Qiao, J. (2015). Aberrant expression of maternal Plk1 and Dctn3 results in the developmental failure of human in-vivo- and in-vitro-matured oocytes. *Sci Rep* 5**,** 8192.

Freed, E.F., Prieto, J.L., Mccann, K.L., Mcstay, B., and Baserga, S.J. (2012). NOL11, implicated in the pathogenesis of North American Indian childhood cirrhosis, is required for pre-rRNA transcription and processing. *PLoS Genet* 8**,** e1002892.

Friedrich, J.K., Panov, K.I., Cabart, P., Russell, J., and Zomerdijk, J.C. (2005). TBP-TAF complex SL1 directs RNA polymerase I pre-initiation complex formation and stabilizes upstream binding factor at the rDNA promoter. *J Biol Chem* 280**,** 29551-29558.

Fu, J., Jiang, M., Mirando, A.J., Yu, H.M., and Hsu, W. (2009). Reciprocal regulation of Wnt and Gpr177/mouse Wntless is required for embryonic axis formation. *Proc Natl Acad Sci U S A* 106**,** 18598-18603.

Goicoechea, S.M., Arneman, D., and Otey, C.A. (2008). The role of palladin in actin organization and cell motility. *Eur J Cell Biol* 87**,** 517-525.

Gomez, H.L., Felipe-Medina, N., Sanchez-Martin, M., Davies, O.R., Ramos, I., Garcia-Tunon, I., De Rooij, D.G., Dereli, I., Toth, A., Barbero, J.L., Benavente, R., Llano, E., and Pendas, A.M. (2016). C14ORF39/SIX6OS1 is a constituent of the synaptonemal complex and is essential for mouse fertility. *Nat Commun* 7**,** 13298.

Gouttenoire, E.A., Lupo, V., Calpena, E., Bartesaghi, L., Schupfer, F., Medard, J.J., Maurer, F., Beckmann, J.S., Senderek, J., Palau, F., Espinos, C., and Chrast, R. (2013). Sh3tc2 deficiency affects neuregulin-1/ErbB signaling. *Glia* 61**,** 1041-1051.

Han, G., Gupta, S.D., Gable, K., Niranjanakumari, S., Moitra, P., Eichler, F., Brown, R.H., Jr., Harmon, J.M., and Dunn, T.M. (2009). Identification of small subunits of mammalian serine palmitoyltransferase that confer distinct acyl-CoA substrate specificities. *Proc Natl Acad Sci U S A* 106**,** 8186-8191.

Han, S., Bahmanyar, S., Zhang, P., Grishin, N., Oegema, K., Crooke, R., Graham, M., Reue, K., Dixon, J.E., and Goodman, J.M. (2012). Nuclear envelope phosphatase 1-regulatory subunit 1 (formerly TMEM188) is the metazoan Spo7p ortholog and functions in the lipin activation pathway. *J Biol Chem* 287**,** 3123-3137.

Hara, M.R., Kovacs, J.J., Whalen, E.J., Rajagopal, S., Strachan, R.T., Grant, W., Towers, A.J., Williams, B., Lam, C.M., Xiao, K., Shenoy, S.K., Gregory, S.G., Ahn, S., Duckett, D.R., and Lefkowitz, R.J. (2011). A stress response pathway regulates DNA damage through beta2-adrenoreceptors and beta-arrestin-1. *Nature* 477**,** 349-353.

Hashimoto, M., Sato, T., Muroyama, Y., Fujimura, L., Hatano, M., and Saito, T. (2015). Nepro is localized in the nucleolus and essential for preimplantation development in mice. *Dev Growth Differ* 57**,** 529-538.

He, D., Wu, Z., He, J., Wang, Y., Li, Z., and Gao, S. (2019). Up-regulation of C5orf34 promotes lung adenocarcinoma migration and is correlated with worse prognosis. *Gene* 696**,** 47-53.

Hendrickx, A., Beullens, M., Ceulemans, H., Den Abt, T., Van Eynde, A., Nicolaescu, E., Lesage, B., and Bollen, M. (2009). Docking motif-guided mapping of the interactome of protein phosphatase-1. *Chem Biol* 16**,** 365-371.

Hernandez, J.B., Chang, C., Leblanc, M., Grimm, D., Le Lay, J., Kaestner, K.H., Zheng, Y., and Montminy, M. (2015). The CREB/CRTC2 pathway modulates autoimmune disease by promoting Th17 differentiation. *Nat Commun* 6**,** 7216.

Hirata, Y., Yamamori, N., Kono, N., Lee, H.C., Inoue, T., and Arai, H. (2013). Identification of small subunit of serine palmitoyltransferase a as a lysophosphatidylinositol acyltransferase 1-interacting protein. *Genes Cells* 18**,** 397-409.

Honda, H., Oda, H., Nakamoto, T., Honda, Z., Sakai, R., Suzuki, T., Saito, T., Nakamura, K., Nakao, K., Ishikawa, T., Katsuki, M., Yazaki, Y., and Hirai, H. (1998). Cardiovascular anomaly, impaired actin bundling and resistance to Src-induced transformation in mice lacking p130Cas. *Nat Genet* 19**,** 361-365.

Hu, D., Smith, E.R., Garruss, A.S., Mohaghegh, N., Varberg, J.M., Lin, C., Jackson, J., Gao, X., Saraf, A., Florens, L., Washburn, M.P., Eissenberg, J.C., and Shilatifard, A. (2013). The little elongation complex functions at initiation and elongation phases of snRNA gene transcription. *Mol Cell* 51**,** 493-505.

Huang, J., Xiao, L., Gong, X., Shao, W., Yin, Y., Liao, Q., Meng, Y., Zhang, Y., Ma, D., and Qiu, X. (2014). Cytokine-like molecule CCDC134 contributes to CD8(+) T-cell effector functions in cancer immunotherapy. *Cancer Res* 74**,** 5734-5745.

Huang, J., Zhang, L., Liu, W., Liao, Q., Shi, T., Xiao, L., Hu, F., and Qiu, X. (2012). CCDC134 interacts with hADA2a and functions as a regulator of hADA2a in acetyltransferase activity, DNA damage-induced apoptosis and cell cycle arrest. *Histochem Cell Biol* 138**,** 41-55.

Hyenne, V., Louvet-Vallee, S., El-Amraoui, A., Petit, C., Maro, B., and Simmler, M.C. (2005). Vezatin, a protein associated to adherens junctions, is required for mouse blastocyst morphogenesis. *Dev Biol* 287**,** 180-191.

Hyenne, V., Souilhol, C., Cohen-Tannoudji, M., Cereghini, S., Petit, C., Langa, F., Maro, B., and Simmler, M.C. (2007). Conditional knock-out reveals that zygotic vezatin-null mouse embryos die at implantation. *Mech Dev* 124**,** 449-462.

Jagannath, A., Butler, R., Godinho, S.I.H., Couch, Y., Brown, L.A., Vasudevan, S.R., Flanagan, K.C., Anthony, D., Churchill, G.C., Wood, M.J.A., Steiner, G., Ebeling, M., Hossbach, M., Wettstein, J.G., Duffield, G.E., Gatti, S., Hankins, M.W., Foster, R.G., and Peirson, S.N. (2013). The CRTC1-SIK1 pathway regulates entrainment of the circadian clock. *Cell* 154**,** 1100-1111.

Jansson, D., Ng, A.C., Fu, A., Depatie, C., Al Azzabi, M., and Screaton, R.A. (2008). Glucose controls CREB activity in islet cells via regulated phosphorylation of TORC2. *Proc Natl Acad Sci U S A* 105**,** 10161-10166.

Jeong, A.L., Ka, H.I., Han, S., Lee, S., Lee, E.W., Soh, S.J., Joo, H.J., Sumiyasuren, B., Park, J.Y., Lim, J.S., Park, J.H., Lee, M.S., and Yang, Y. (2018). Oncoprotein CIP2A promotes the disassembly of primary cilia and inhibits glycolytic metabolism. *EMBO Rep* 19.

Jeong, A.L., Lee, S., Park, J.S., Han, S., Jang, C.Y., Lim, J.S., Lee, M.S., and Yang, Y. (2014). Cancerous inhibitor of protein phosphatase 2A (CIP2A) protein is involved in centrosome separation through the regulation of NIMA (never in mitosis gene A)-related kinase 2 (NEK2) protein activity. *J Biol Chem* 289**,** 28-40.

Kang, J., Shi, Y., Xiang, B., Qu, B., Su, W., Zhu, M., Zhang, M., Bao, G., Wang, F., Zhang, X., Yang, R., Fan, F., Chen, X., Pei, G., and Ma, L. (2005). A nuclear function of beta-arrestin1 in GPCR signaling: regulation of histone acetylation and gene transcription. *Cell* 123**,** 833-847.

Kerosuo, L., Fox, H., Perala, N., Ahlqvist, K., Suomalainen, A., Westermarck, J., Sariola, H., and Wartiovaara, K. (2010). CIP2A increases self-renewal and is linked to Myc in neural progenitor cells. *Differentiation* 80**,** 68-77.

Kilmartin, J.V. (2003). Sfi1p has conserved centrin-binding sites and an essential function in budding yeast spindle pole body duplication. *J Cell Biol* 162**,** 1211-1221.

Kim, H. (2016). The transcription cofactor CRTC1 protects from aberrant hepatic lipid accumulation. *Sci Rep* 6**,** 37280.

Kim, J., Choi, T.I., Park, S., Kim, M.H., Kim, C.H., and Lee, S. (2018). Rnf220 cooperates with Zc4h2 to specify spinal progenitor domains. *Development* 145.

Kim, J.H., Hedrick, S., Tsai, W.W., Wiater, E., Le Lay, J., Kaestner, K.H., Leblanc, M., Loar, A., and Montminy, M. (2017). CREB coactivators CRTC2 and CRTC3 modulate bone marrow hematopoiesis. *Proc Natl Acad Sci U S A* 114**,** 11739-11744.

Ko, H.W., Norman, R.X., Tran, J., Fuller, K.P., Fukuda, M., and Eggenschwiler, J.T. (2010). Broad-minded links cell cycle-related kinase to cilia assembly and hedgehog signal transduction. *Dev Cell* 18**,** 237-247.

Kong, Q., Zeng, W., Wu, J., Hu, W., Li, C., and Mao, B. (2010). RNF220, an E3 ubiquitin ligase that targets Sin3B for ubiquitination. *Biochem Biophys Res Commun* 393**,** 708-713.

Koo, S.H., Flechner, L., Qi, L., Zhang, X., Screaton, R.A., Jeffries, S., Hedrick, S., Xu, W., Boussouar, F., Brindle, P., Takemori, H., and Montminy, M. (2005). The CREB coactivator TORC2 is a key regulator of fasting glucose metabolism. *Nature* 437**,** 1109-1111.

Korotkov, K.V., Novoselov, S.V., Hatfield, D.L., and Gladyshev, V.N. (2002). Mammalian selenoprotein in which selenocysteine (Sec) incorporation is supported by a new form of Sec insertion sequence element. *Mol Cell Biol* 22**,** 1402-1411.

Kovacs, K.A., Steullet, P., Steinmann, M., Do, K.Q., Magistretti, P.J., Halfon, O., and Cardinaux, J.R. (2007). TORC1 is a calcium- and cAMP-sensitive coincidence detector involved in hippocampal long-term synaptic plasticity. *Proc Natl Acad Sci U S A* 104**,** 4700-4705.

Kussel-Andermann, P., El-Amraoui, A., Safieddine, S., Nouaille, S., Perfettini, I., Lecuit, M., Cossart, P., Wolfrum, U., and Petit, C. (2000). Vezatin, a novel transmembrane protein, bridges myosin VIIA to the cadherin-catenins complex. *EMBO J* 19**,** 6020-6029.

Lerner, R.G., Depatie, C., Rutter, G.A., Screaton, R.A., and Balthasar, N. (2009). A role for the CREB co-activator CRTC2 in the hypothalamic mechanisms linking glucose sensing with gene regulation. *EMBO Rep* 10**,** 1175-1181.

Libby, B.J., De La Fuente, R., O'brien, M.J., Wigglesworth, K., Cobb, J., Inselman, A., Eaker, S., Handel, M.A., Eppig, J.J., and Schimenti, J.C. (2002). The mouse meiotic mutation mei1 disrupts chromosome synapsis with sexually dimorphic consequences for meiotic progression. *Dev Biol* 242**,** 174-187.

Liebe, B., Petukhova, G., Barchi, M., Bellani, M., Braselmann, H., Nakano, T., Pandita, T.K., Jasin, M., Fornace, A., Meistrich, M.L., Baarends, W.M., Schimenti, J., De Lange, T., Keeney, S., Camerini-Otero, R.D., and Scherthan, H. (2006). Mutations that affect meiosis in male mice influence the dynamics of the mid-preleptotene and bouquet stages. *Exp Cell Res* 312**,** 3768-3781.

Liu, S.S., Zheng, H.X., Jiang, H.D., He, J., Yu, Y., Qu, Y.P., Yue, L., Zhang, Y., and Li, Y. (2012). Identification and characterization of a novel gene, c1orf109, encoding a CK2 substrate that is involved in cancer cell proliferation. *J Biomed Sci* 19**,** 49.

Luo, H., Liu, X., Wang, F., Huang, Q., Shen, S., Wang, L., Xu, G., Sun, X., Kong, H., Gu, M., Chen, S., Chen, Z., and Wang, Z. (2005). Disruption of palladin results in neural tube closure defects in mice. *Mol Cell Neurosci* 29**,** 507-515.

Ma, P., Yang, X., Kong, Q., Li, C., Yang, S., Li, Y., and Mao, B. (2014). The ubiquitin ligase RNF220 enhances canonical Wnt signaling through USP7-mediated deubiquitination of beta-catenin. *Mol Cell Biol* 34**,** 4355-4366.

Mccleland, M.L., Kallio, M.J., Barrett-Wilt, G.A., Kestner, C.A., Shabanowitz, J., Hunt, D.F., Gorbsky, G.J., and Stukenberg, P.T. (2004). The vertebrate Ndc80 complex contains Spc24 and Spc25 homologs, which are required to establish and maintain kinetochore-microtubule attachment. *Curr Biol* 14**,** 131-137.

Mick, D.U., Dennerlein, S., Wiese, H., Reinhold, R., Pacheu-Grau, D., Lorenzi, I., Sasarman, F., Weraarpachai, W., Shoubridge, E.A., Warscheid, B., and Rehling, P. (2012). MITRAC links mitochondrial protein translocation to respiratory-chain assembly and translational regulation. *Cell* 151**,** 1528-1541.

Moyer, B.D., Hevezi, P., Gao, N., Lu, M., Kalabat, D., Soto, H., Echeverri, F., Laita, B., Yeh, S.A., Zoller, M., and Zlotnik, A. (2009). Expression of genes encoding multi-transmembrane proteins in specific primate taste cell populations. *PLoS One* 4**,** e7682.

Muroyama, Y., and Saito, T. (2009). Identification of Nepro, a gene required for the maintenance of neocortex neural progenitor cells downstream of Notch. *Development* 136**,** 3889-3893.

Najdi, R., Proffitt, K., Sprowl, S., Kaur, S., Yu, J., Covey, T.M., Virshup, D.M., and Waterman, M.L. (2012). A uniform human Wnt expression library reveals a shared secretory pathway and unique signaling activities. *Differentiation* 84**,** 203-213.

Nakano, Y., Longo-Guess, C.M., Bergstrom, D.E., Nauseef, W.M., Jones, S.M., and Banfi, B. (2008). Mutation of the Cyba gene encoding p22phox causes vestibular and immune defects in mice. *J Clin Invest* 118**,** 1176-1185.

Navarro Negredo, P., Edgar, J.R., Manna, P.T., Antrobus, R., and Robinson, M.S. (2018). The WDR11 complex facilitates the tethering of AP-1-derived vesicles. *Nat Commun* 9**,** 596.

Nguyen, N.U., Liang, V.R., and Wang, H.V. (2014). Actin-associated protein palladin is required for migration behavior and differentiation potential of C2C12 myoblast cells. *Biochem Biophys Res Commun* 452**,** 728-733.

Nikonov, S.S., Brown, B.M., Davis, J.A., Zuniga, F.I., Bragin, A., Pugh, E.N., Jr., and Craft, C.M. (2008). Mouse cones require an arrestin for normal inactivation of phototransduction. *Neuron* 59**,** 462-474.

Nishijima, I., and Ohtoshi, A. (2006). Characterization of a novel prospero-related homeobox gene, Prox2. *Mol Genet Genomics* 275**,** 471-478.

Nonaka, M., Kim, R., Fukushima, H., Sasaki, K., Suzuki, K., Okamura, M., Ishii, Y., Kawashima, T., Kamijo, S., Takemoto-Kimura, S., Okuno, H., Kida, S., and Bito, H. (2014). Region-specific activation of CRTC1-CREB signaling mediates long-term fear memory. *Neuron* 84**,** 92-106.

Okita, K., Kiyonari, H., Nobuhisa, I., Kimura, N., Aizawa, S., and Taga, T. (2004). Targeted disruption of the mouse ELYS gene results in embryonic death at peri-implantation development. *Genes Cells* 9**,** 1083-1091.

Ozaki, Y., Matsui, H., Nagamachi, A., Asou, H., Aki, D., and Inaba, T. (2011). The dynactin complex maintains the integrity of metaphasic centrosomes to ensure transition to anaphase. *J Biol Chem* 286**,** 5589-5598.

Parra-Damas, A., Rubio-Ferrarons, L., Shen, J., and Saura, C.A. (2017). CRTC1 mediates preferential transcription at neuronal activity-regulated CRE/TATA promoters. *Sci Rep* 7**,** 18004.

Peng, A., Lewellyn, A.L., Schiemann, W.P., and Maller, J.L. (2010). Repo-man controls a protein phosphatase 1-dependent threshold for DNA damage checkpoint activation. *Curr Biol* 20**,** 387-396.

Peterson, Y.K., and Luttrell, L.M. (2017). The Diverse Roles of Arrestin Scaffolds in G Protein-Coupled Receptor Signaling. *Pharmacol Rev* 69**,** 256-297.

Pitts, M.W., Reeves, M.A., Hashimoto, A.C., Ogawa, A., Kremer, P., Seale, L.A., and Berry, M.J. (2013). Deletion of selenoprotein M leads to obesity without cognitive deficits. *J Biol Chem* 288**,** 26121-26134.

Qian, J., Lesage, B., Beullens, M., Van Eynde, A., and Bollen, M. (2011). PP1/Repo-man dephosphorylates mitotic histone H3 at T3 and regulates chromosomal aurora B targeting. *Curr Biol* 21**,** 766-773.

Ranjan, R., Dwivedi, H., Baidya, M., Kumar, M., and Shukla, A.K. (2017). Novel Structural Insights into GPCR-beta-Arrestin Interaction and Signaling. *Trends Cell Biol* 27**,** 851-862.

Rasala, B.A., Orjalo, A.V., Shen, Z., Briggs, S., and Forbes, D.J. (2006). ELYS is a dual nucleoporin/kinetochore protein required for nuclear pore assembly and proper cell division. *Proc Natl Acad Sci U S A* 103**,** 17801-17806.

Raschle, M., Smeenk, G., Hansen, R.K., Temu, T., Oka, Y., Hein, M.Y., Nagaraj, N., Long, D.T., Walter, J.C., Hofmann, K., Storchova, Z., Cox, J., Bekker-Jensen, S., Mailand, N., and Mann, M. (2015). DNA repair. Proteomics reveals dynamic assembly of repair complexes during bypass of DNA cross-links. *Science* 348**,** 1253671.

Reeves, M.A., Bellinger, F.P., and Berry, M.J. (2010). The neuroprotective functions of selenoprotein M and its role in cytosolic calcium regulation. *Antioxid Redox Signal* 12**,** 809-818.

Reinholdt, L.G., and Schimenti, J.C. (2005). Mei1 is epistatic to Dmc1 during mouse meiosis. *Chromosoma* 114**,** 127-134.

Robert, T., Nore, A., Brun, C., Maffre, C., Crimi, B., Bourbon, H.M., and De Massy, B. (2016). The TopoVIB-Like protein family is required for meiotic DNA double-strand break formation. *Science* 351**,** 943-949.

Roberts, R.C., Peden, A.A., Buss, F., Bright, N.A., Latouche, M., Reilly, M.M., Kendrick-Jones, J., and Luzio, J.P. (2010). Mistargeting of SH3TC2 away from the recycling endosome causes Charcot-Marie-Tooth disease type 4C. *Hum Mol Genet* 19**,** 1009-1018.

Rosano, L., and Bagnato, A. (2016). beta-arrestin1 at the cross-road of endothelin-1 signaling in cancer. *J Exp Clin Cancer Res* 35**,** 121.

Screaton, R.A., Conkright, M.D., Katoh, Y., Best, J.L., Canettieri, G., Jeffries, S., Guzman, E., Niessen, S., Yates, J.R., 3rd, Takemori, H., Okamoto, M., and Montminy, M. (2004). The CREB coactivator TORC2 functions as a calcium- and cAMP-sensitive coincidence detector. *Cell* 119**,** 61-74.

Sekeres, M.J., Mercaldo, V., Richards, B., Sargin, D., Mahadevan, V., Woodin, M.A., Frankland, P.W., and Josselyn, S.A. (2012). Increasing CRTC1 function in the dentate gyrus during memory formation or reactivation increases memory strength without compromising memory quality. *J Neurosci* 32**,** 17857-17868.

Sekiguchi, T., Hayano, T., Yanagida, M., Takahashi, N., and Nishimoto, T. (2006). NOP132 is required for proper nucleolus localization of DEAD-box RNA helicase DDX47. *Nucleic Acids Res* 34**,** 4593-4608.

Sellier, C., Campanari, M.L., Julie Corbier, C., Gaucherot, A., Kolb-Cheynel, I., Oulad-Abdelghani, M., Ruffenach, F., Page, A., Ciura, S., Kabashi, E., and Charlet-Berguerand, N. (2016). Loss of C9ORF72 impairs autophagy and synergizes with polyQ Ataxin-2 to induce motor neuron dysfunction and cell death. *EMBO J* 35**,** 1276-1297.

Shahmoradi, L., Ahmadi, M., Sadoughi, F., Piri, Z., and Gohari, M.R. (2015). A comprehensive model for executing knowledge management audits in organizations: a systematic review. *Health Care Manag (Frederick)* 34**,** 28-40.

Shan, T., Xiong, Y., Zhang, P., Li, Z., Jiang, Q., Bi, P., Yue, F., Yang, G., Wang, Y., Liu, X., and Kuang, S. (2016). Lkb1 controls brown adipose tissue growth and thermogenesis by regulating the intracellular localization of CRTC3. *Nat Commun* 7**,** 12205.

Shibuya, H., Hernandez-Hernandez, A., Morimoto, A., Negishi, L., Hoog, C., and Watanabe, Y. (2015). MAJIN Links Telomeric DNA to the Nuclear Membrane by Exchanging Telomere Cap. *Cell* 163**,** 1252-1266.

Smith, E.R., Lin, C., Garrett, A.S., Thornton, J., Mohaghegh, N., Hu, D., Jackson, J., Saraf, A., Swanson, S.K., Seidel, C., Florens, L., Washburn, M.P., Eissenberg, J.C., and Shilatifard, A. (2011). The little elongation complex regulates small nuclear RNA transcription. *Mol Cell* 44**,** 954-965.

Song, Y., Altarejos, J., Goodarzi, M.O., Inoue, H., Guo, X., Berdeaux, R., Kim, J.H., Goode, J., Igata, M., Paz, J.C., Hogan, M.F., Singh, P.K., Goebel, N., Vera, L., Miller, N., Cui, J., Jones, M.R., Consortium, C., Consortium, G., Chen, Y.D., Taylor, K.D., Hsueh, W.A., Rotter, J.I., and Montminy, M. (2010). CRTC3 links catecholamine signalling to energy balance. *Nature* 468**,** 933-939.

Stasia, M.J. (2016). CYBA encoding p22(phox), the cytochrome b558 alpha polypeptide: gene structure, expression, role and physiopathology. *Gene* 586**,** 27-35.

Stendel, C., Roos, A., Kleine, H., Arnaud, E., Ozcelik, M., Sidiropoulos, P.N., Zenker, J., Schupfer, F., Lehmann, U., Sobota, R.M., Litchfield, D.W., Luscher, B., Chrast, R., Suter, U., and Senderek, J. (2010). SH3TC2, a protein mutant in Charcot-Marie-Tooth neuropathy, links peripheral nerve myelination to endosomal recycling. *Brain* 133**,** 2462-2474.

Stergiopoulos, A., Elkouris, M., and Politis, P.K. (2014). Prospero-related homeobox 1 (Prox1) at the crossroads of diverse pathways during adult neural fate specification. *Front Cell Neurosci* 8**,** 454.

Sullivan, P.M., Zhou, X., Robins, A.M., Paushter, D.H., Kim, D., Smolka, M.B., and Hu, F. (2016). The ALS/FTLD associated protein C9orf72 associates with SMCR8 and WDR41 to regulate the autophagy-lysosome pathway. *Acta Neuropathol Commun* 4**,** 51.

Sun, H.M., Chen, X.L., Chen, X.J., Liu, J., Ma, L., Wu, H.Y., Huang, Q.H., Xi, X.D., Yin, T., Zhu, J., Chen, Z., and Chen, S.J. (2017). PALLD Regulates Phagocytosis by Enabling Timely Actin Polymerization and Depolymerization. *J Immunol* 199**,** 1817-1826.

Thomas, S., Legendre, M., Saunier, S., Bessieres, B., Alby, C., Bonniere, M., Toutain, A., Loeuillet, L., Szymanska, K., Jossic, F., Gaillard, D., Yacoubi, M.T., Mougou-Zerelli, S., David, A., Barthez, M.A., Ville, Y., Bole-Feysot, C., Nitschke, P., Lyonnet, S., Munnich, A., Johnson, C.A., Encha-Razavi, F., Cormier-Daire, V., Thauvin-Robinet, C., Vekemans, M., and Attie-Bitach, T. (2012). TCTN3 mutations cause Mohr-Majewski syndrome. *Am J Hum Genet* 91**,** 372-378.

Tian, X., Kang, D.S., and Benovic, J.L. (2014). beta-arrestins and G protein-coupled receptor trafficking. *Handb Exp Pharmacol* 219**,** 173-186.

Tikhmyanova, N., Little, J.L., and Golemis, E.A. (2010). CAS proteins in normal and pathological cell growth control. *Cell Mol Life Sci* 67**,** 1025-1048.

Tipton, A.R., Wang, K., Oladimeji, P., Sufi, S., Gu, Z., and Liu, S.T. (2012). Identification of novel mitosis regulators through data mining with human centromere/kinetochore proteins as group queries. *BMC Cell Biol* 13**,** 15.

Trinkle-Mulcahy, L., Andersen, J., Lam, Y.W., Moorhead, G., Mann, M., and Lamond, A.I. (2006). Repo-Man recruits PP1 gamma to chromatin and is essential for cell viability. *J Cell Biol* 172**,** 679-692.

Vagnarelli, P., and Earnshaw, W.C. (2012). Repo-Man-PP1: a link between chromatin remodelling and nuclear envelope reassembly. *Nucleus* 3**,** 138-142.

Vagnarelli, P., Hudson, D.F., Ribeiro, S.A., Trinkle-Mulcahy, L., Spence, J.M., Lai, F., Farr, C.J., Lamond, A.I., and Earnshaw, W.C. (2006). Condensin and Repo-Man-PP1 co-operate in the regulation of chromosome architecture during mitosis. *Nat Cell Biol* 8**,** 1133-1142.

Vagnarelli, P., Ribeiro, S., Sennels, L., Sanchez-Pulido, L., De Lima Alves, F., Verheyen, T., Kelly, D.A., Ponting, C.P., Rappsilber, J., and Earnshaw, W.C. (2011). Repo-Man coordinates chromosomal reorganization with nuclear envelope reassembly during mitotic exit. *Dev Cell* 21**,** 328-342.

Ventela, S., Come, C., Makela, J.A., Hobbs, R.M., Mannermaa, L., Kallajoki, M., Chan, E.K., Pandolfi, P.P., Toppari, J., and Westermarck, J. (2012). CIP2A promotes proliferation of spermatogonial progenitor cells and spermatogenesis in mice. *PLoS One* 7**,** e33209.

Wang, B., Zhang, Y., Dong, H., Gong, S., Wei, B., Luo, M., Wang, H., Wu, X., Liu, W., Xu, X., Zheng, Y., and Sun, M. (2018a). Loss of Tctn3 causes neuronal apoptosis and neural tube defects in mice. *Cell Death Dis* 9**,** 520.

Wang, C., Li, J., Meng, Q., and Wang, B. (2017). Three Tctn proteins are functionally conserved in the regulation of neural tube patterning and Gli3 processing but not ciliogenesis and Hedgehog signaling in the mouse. *Dev Biol* 430**,** 156-165.

Wang, C., Li, J., Takemaru, K.I., Jiang, X., Xu, G., and Wang, B. (2018b). Centrosomal protein Dzip1l binds Cby, promotes ciliary bud formation, and acts redundantly with Bromi to regulate ciliogenesis in the mouse. *Development* 145.

Wang, C., Zeng, X., Zhou, Z., Zhao, J., and Pei, G. (2016). beta-arrestin-1 contributes to brown fat function and directly interacts with PPARalpha and PPARgamma. *Sci Rep* 6**,** 26999.

Wang, Y., Chen, Y., Chen, J., Wang, L., Nie, L., Long, J., Chang, H., Wu, J., Huang, C., and Lei, M. (2019). The meiotic TERB1-TERB2-MAJIN complex tethers telomeres to the nuclear envelope. *Nat Commun* 10**,** 564.

Wang, Y., Tang, Y., Teng, L., Wu, Y., Zhao, X., and Pei, G. (2006). Association of beta-arrestin and TRAF6 negatively regulates Toll-like receptor-interleukin 1 receptor signaling. *Nat Immunol* 7**,** 139-147.

Wang, Y., Vera, L., Fischer, W.H., and Montminy, M. (2009). The CREB coactivator CRTC2 links hepatic ER stress and fasting gluconeogenesis. *Nature* 460**,** 534-537.

Wurzenberger, C., Held, M., Lampson, M.A., Poser, I., Hyman, A.A., and Gerlich, D.W. (2012). Sds22 and Repo-Man stabilize chromosome segregation by counteracting Aurora B on anaphase kinetochores. *J Cell Biol* 198**,** 173-183.

Yang, X., Zhou, G., Ren, T., Li, H., Zhang, Y., Yin, D., Qian, H., and Li, Q. (2012). beta-Arrestin prevents cell apoptosis through pro-apoptotic ERK1/2 and p38 MAPKs and anti-apoptotic Akt pathways. *Apoptosis* 17**,** 1019-1026.

Yoon, Y.S., Tsai, W.W., Van De Velde, S., Chen, Z., Lee, K.F., Morgan, D.A., Rahmouni, K., Matsumura, S., Wiater, E., Song, Y., and Montminy, M. (2018). cAMP-inducible coactivator CRTC3 attenuates brown adipose tissue thermogenesis. *Proc Natl Acad Sci U S A* 115**,** E5289-E5297.

Yu, B., Zhang, T., Xia, P., Gong, X., Qiu, X., and Huang, J. (2018). CCDC134 serves a crucial role in embryonic development. *Int J Mol Med* 41**,** 381-390.

Yu, J., Chia, J., Canning, C.A., Jones, C.M., Bard, F.A., and Virshup, D.M. (2014). WLS retrograde transport to the endoplasmic reticulum during Wnt secretion. *Dev Cell* 29**,** 277-291.

Yu, Y., Liu, C., Zhang, J., Zhang, M., Wen, W., Ruan, X., Li, D., Zhang, S., Gao, M., and Chen, L. (2017). Rtfc (4931414P19Rik) Regulates in vitro Thyroid Differentiation and in vivo Thyroid Function. *Sci Rep* 7**,** 43396.

Zhang, X., Chen, X., Liu, J., Xu, X., Zhang, Y., Ruan, Z., Xie, Y., Huang, Q., Yin, T., Chen, Z., and Chen, S. (2017). Palladin is a novel microtubule-associated protein responsible for spindle orientation. *Sci Rep* 7**,** 11806.
