# Supplementary material for: An Integrated Model of Minor Intron Emergence and Conservation
Source: Front Genet. 2019 Nov 13;10:1113. doi: 10.3389/fgene.2019.01113 (PMC6865273; doi:10.3389/fgene.2019.01113)
Supplement: Supplementary file 3 [file Table_3.docx]

**Supplementary Table 3. Mammalian functions of the MIGs identified in the majority essentialome.** Column three (“Core Essential.”=core essentialome) indicates whether that MIG is found in the core essentialome, while column four (“Ancient Essent.”=ancient essentialome) notes whether that MIG traces back to the last eukaryotic common ancestor (LECA) or earlier (Y=yes, N=no). Based on the findings of literature curation, listed in the Function Description column, MIGs were classified into different functional categories (sixth column). Syn=synonyms.

| **MIG** | **Syn** | **Core**  **Essent.?** | **Ancient**  **Essent.?** | **Function Description** | **Functional Categories** |
| --- | --- | --- | --- | --- | --- |
| *ACTL6A* | *BAF53A* | N | Y | part of chromatin remodeling complex (Lessard et al., 2007; Bao et al., 2013); necessary and sufficient for neural progenitor proliferation (Lessard et al., 2007); constitutive knockout is early embryonic lethal (Krasteva et al., 2012) | transcription; cell cycle (proliferation); development (central nervous system) |
| *AHCTF1* | *ELYS* | N | N | in HeLa cells, localizes to nuclear pore and kinetochores, and RNAi-mediated knockdown causes cytokinesis defects (Rasala et al., 2006); required for nuclear pore assembly (Rasala et al., 2006); siRNA-mediated knockdown in HeLa cells results in mislocalization of LBR, which is important for reforming nuclear envelope post-mitosis (Clever et al., 2012); mouse knockout results in early embryonic lethality (Okita et al., 2004) | cell cycle (mitosis/cytokinesis) |
| *ARL2* | *ARFL2* | N | Y | centrosomal protein that regulates microtubule nucleation and organization (Zhou et al., 2006); a fraction of ARL2 protein localizes to the mitochondria, where it regulates ATP levels and mitochondrial integrity (Newman et al., 2014); based on work in non-mammalian model systems, mutations in ARF2 may cause shortening of microtubules, aberrant microtubule and spindle organization, cell division and cytokinesis defects (Antoshechkin and Han, 2002; Price et al., 2010) | metabolism; cell cycle (mitosis/  cytokinesis) |
| *ATP6V1A* | *ATP6V1A1* | N | Y | acidifying vacuolar ATPase (vATPase) subunit A (van Hille et al., 1993) | ion transport |
| *BRF1* | *CFDS, GTF3B, TAF3C, TAF3B2, TAFIII90, TIS11b* | N | Y | the 90 kDa beta subunit of RNA polymerase III (Wang and Roeder, 1995); regulates degradation of a subset of mRNAs with AU-rich elements (AREs) in their 3'-untranslated region (Stoecklin et al., 2002; Raineri et al., 2004); via its role in ARE-dependent mRNA turnover, regulates degree of proliferation in mouse embryonic stem cells (Tan and Elowitz, 2014) | transcription; non-coding RNA biogenesis; cell cycle (proliferation) |
| **MIG** | **Syn** | **Core**  **Essent.?** | **Ancient**  **Essent.?** | **Function Description** | **Functional Categories** |
| *C1orf109* |  | N | N | functions in cancer cell proliferation, based on misexpression and knockdown studies (Liu et al., 2012) | cell cycle (proliferation) |
| *C3orf17* | *NEPRO, NET17* | N | N | involved in maintenance of neocortex neural progenitor cells downstream of Notch and plays a role in repression of proneural gene expression; misexpression causes inhibition of neuronal differentiation in the early neocortex, while knockdown drives neuron differentiation (Muroyama and Saito, 2009); localized to the nucleolus, and knockout in mice causes impaired blastocyst formation and apoptosis (Hashimoto et al., 2015) | cell cycle (proliferation/  progenitor cell identity); development (central nervous system) |
| *CDC45* | *CDC45L* | Y | Y | initiation of DNA replication (Gerhardt et al., 2015); recruits DNA polymerase α-primase (see *PRIM1* below) to the DNA replication complex (Kukimoto et al., 1999) | cell cycle (DNA replication/S phase) |
| *CHD4* | *Mi-2β* | N | Y | a catalytic subunit of the nucleosome remodeling and deacetylase (NuRD) complex (Xue et al., 1998; Zhang et al., 1998); controls chromatin relaxation prior to repair of double-stranded DNA breaks (Larsen et al., 2010; Polo et al., 2010; Urquhart et al., 2011; Pan et al., 2012); via roles in chromatin remodeling and p53 deacetylation, regulates self-renewel of embryonic stem cells and cell cycle progression (Larsen et al., 2010; Zhao et al., 2017) | transcription regulation; genome integrity (DNA repair); cell cycle (progression) |
| *CNIH4* | *CNIH2* | N | Y | encodes a GPCR-interacting protein that functions in GPCR export from the endoplasmic reticulum (ER) in the early secretory pathway (Sauvageau et al., 2014) | transport (ER to Golgi) |
| *CRNKL1* | *CLF*, *CRN, HCRN, SYF3* | Y | Y | encodes a protein component of the spliceosome (Chung et al., 2002; Bertram et al., 2017b) | RNA processing (mRNA splicing) |
| *DCTN6* | *WS3,* p27 | N | Y | encodes a subunit of dynactin, located in the pointed end complex (Eckley et al., 1999); via its role as a binding partner for PLK1, regulates the spindle assembly checkpoint and mitotic progression (Yeh et al., 2013) | transport (organelles), cell cycle (mitosis) |
| *DDB1* | *XPE, DDBA* | Y | Y | large subunit of DNA damage-binding complex (Yeh et al., 2012); nucleotide excision repair (Li et al., 2006); involved in ubiquitin… | genome integrity (DNA repair) |
| **MIG** | **Syn** | **Core**  **Essent.?** | **Ancient**  **Essent.?** | **Function Description** | **Functional Categories** |
| *DDB1* | *XPE, DDBA, XAP1, XPCE* | Y | Y | … complex via interaction with the MIG CUL4A (Leung-Pineda et al., 2009); constitutive ablation is embryonically lethal in mice, and brain-specific ablation leads to accumulation of cell cycle regulators, genomic instability, and apoptosis of proliferating NPCs (Cang et al., 2006) | genome integrity (DNA repair) |
| *DDX54* | *DP97* | ~~N~~ | Y | RNA helicase that localizes to the nucleolus; in the DNA damage response, increases splicing efficiency of pre-mRNA transcripts generated in response to DNA damage (Milek et al., 2017); interacts with constitutive androstane nuclear receptor (response to xeno-chemical stimuli) and acts as co-activator to upregulate expression of downstream genes involved in drug metabolism (Kanno et al., 2012); estrogen-dependent interaction with nuclear estrogen receptors results in inhibition of transcriptional activity (Rajendran et al., 2003); constitutive ablation is early embryonically lethal (Dickinson et al., 2016); binds to myelin basic protein (MBP) in oligodendrocytes, and knockdown causes depletion of MBP (Zhan et al., 2013) | transcription regulation; RNA processing (splicing); stress response; neuron function (myelination) |
| *DONSON* | *MIMIS, B17, MSSLA, C24orf60* | Y | Y | a replisome protein that functions in stabilization of the replication fork and the intra-S phase checkpoint (Reynolds et al., 2017); mutation causes microcephalic dwarfism (Evrony et al., 2017; Reynolds et al., 2017); constitutive ablation is early embryonic lethal in mice (Evrony et al., 2017) | genome integrity (DNA repair); cell cycle (checkpoint control); development |
| *EIF3I* | *TRIP1, EIF3S2, PRO2242, eIF3-beta* | Y | Y | translation initiation factor, but not required for translation initiation (Masutani et al., 2007); in intestinal epithelial cells, ectopic overexpression triggers oncogenesis (Qi et al., 2014); mTOR directly interacts with eIF3 (all Eif3s, not just the product of this gene) to increase association to eIF4G (Harris et al., 2006); regulates osteoblast differentiation and proliferation, and knockdown decreases the number of cells in S-phase while increasing cells in G2/M phase (Metz-Estrella et al., 2012); in vitro overexpression triggers increased cell size, increased proliferation, and cell cycle progression (Ahlemann et al., 2006) | translation; cell cycle; development |
| **MIG** | **Syn** | **Core**  **Essent.?** | **Ancient**  **Essent.?** | **Function Description** | **Functional Categories** |
| *ELP3* | *KAT9* | N | Y | encodes a 58 kDa histone acetyltransferase that functions as the catalytic subunit of the Elongator complex, which interacts with RNA polymerase II to facilitate transcription through chromatin, although this role has been questioned in recent years (Hawkes et al., 2002; Kim et al., 2002; Dalwadi and Yip, 2018); plays a conserved role in translation regulation via wobble uridine tRNA modification (Huang et al., 2005; Mehlgarten et al., 2010; Selvadurai et al., 2014; Yoo et al., 2016) | transcription regulation; non-coding RNA biogenesis (tRNA); translation regulation |
| *EXOSC2* | *RRP4, p7, Rrp4p* | N | Y | exosome component; high affinity for binding to phosphorylated Upf1 (involv-ed in nonsense mediated decay pathway; considered signal to recruit mRNA degradation factors to transcript) (Lejeune et al., 2003; Isken et al., 2008); in HEp-2 cells, required for exosome stability, with knockdown inhibiting cell growth (van Dijk et al., 2007); in yeast, required for processing of 7S pre-rRNA to 5.8S rRNA (Mitchell et al., 1996) | RNA metabolism |
| *FAM96B* | *CIAO2B, CIA2B, MIP18* | N | Y | component of the MMXD complex; possibly required for Aurora B localization; localized to mitotic spindle, and knockdown results in abnormal mitotic spindle formation, chromosome missegregation, and multi-nucleation (Ito et al., 2010); interacts with and downregulates E2-2 (role in endothelial cell quiescence) while also enhancing endothelial migration, proliferation, and tube formation (Yang et al., 2011); knockdown experiments indicate target specificity of its Fe-S assembly activity (necessary for maturation of nucleotide metabolism proteins DPYD and GPAT; interacts with the MIG *DNA2*); involved in the regulation of iron homeostasis by decreasing IRE-binding activity and protein levels of IRP2 (Stehling et al., 2013) | cell cycle (mitosis); iron homeostasis |
| *GARS* | *HMN5, CMT2D, DSMAV, SMAD1* | N | Y | glycyl-tRNA synthetase, which covalently links glycine with corresponding tRNAs (Motley et al., 2010); mutations are linked to Charcot-Marie-Tooth (CMT) disease, which specifically affects neurons (Motley et al., 2010); constitutive genetic disruption in mice is embryonically lethal (Seburn et al., 2006) | translation |
| **MIG** | **Syn** | **Core**  **Essent.?** | **Ancient**  **Essent.?** | **Function Description** | **Functional Categories** |
| *GPN1* | *XAB1, MBDIN, NTPBP, RPAP4* | Y | Y | a GTPase that, along with GPN3, is required for the nuclear translocation of RNA polymerase II subunits (Forget et al., 2010; Carre and Shiekhattar, 2011); also regulates the nuclear translocation of XPA, a powerful regulator of nucleotide excision repair (Dong et al., 2010) | transport (nucleocytoplasmic); transcription regulation; genome integrity (DNA repair) |
| *GPN2* | *ATPBD1B* | Y | Y | a GTPase that, in yeast, is required for the nuclear translocation of RNA polymerase II and III subunits and regulates the assembly of RNA polymerase II (Minaker et al., 2013; Zeng et al., 2018) | transport (nucleocytoplasmic); transcription regulation |
| *GPN3* | *ATPBD1C* | Y | Y | a GTPase that, along with GPN1, is required for the nuclear translocation of RNA polymerase II subunits (Calera et al., 2011; Carre and Shiekhattar, 2011); in yeast, regulates the nuclear translocation of RNA polymerase III subunits (Minaker et al., 2013) | transport (nucleocytoplasmic); transcription regulation |
| *HARS* | *HARS1, USH3B* | Y | Y | encodes histidyl-tRNA synthetase (Wasmuth and Carlock, 1986) | translation |
| *IK* | *RED, RER, CSA2* | Y | Y | initially identified as a cytokine that inhibits IFN-gamma-mediated upregulation of MHC class II antigens (Krief et al., 1994); plays multiple roles in mitotic progression, such as the localization of the spindle assembly checkpoint protein MAD1 and the recruitment PP2A to dephosphorylate Aurora B (Yeh et al., 2012; Lee et al., 2014; Lee et al., 2016); found in the spliceosomal B complex, linking the U2 and U5 snRNPs (Bertram et al., 2017a) | cell cycle (mitosis); RNA processing (mRNA splicing) |
| *INTS4* | *INT4* | N | Y | one of 12 subunits of the Integrator complex, which regulates the 3'-end processing of snRNAs (Baillat et al., 2005); required for snRNA export from the nucleus and for proper formation of the Cajal bodies, the site of snRNP and snoRNP maturation (Takata et al., 2012) | non-coding RNA biogenesis (snRNA); RNA processing (mRNA splicing) |
| *LSM8* | *NAA38* | N | Y | one of 7 Sm-like proteins that, together, function in the maturation of rRNAs, tRNAs and the U6 spliceosomal snRNA (Achsel et al., 1999; Kufel et al., 2002; Kufel et al., 2003); found in the spliceosomal B complex, in the U4/U6.U5 tri-snRNP (Bertram et al., 2017a); knockdown results in upregulation of processing… | non-coding RNA biogenesis; RNA processing (mRNA splicing) |
| **MIG** | **Syn** | **Core**  **Essent.?** | **Ancient**  **Essent.?** | **Function Description** | **Functional Categories** |
| *LSM8* | *NAA38* | N | Y | … bodies, formed of LSM1-7, which mediate mRNA degradation (Novotny et al., 2012) | non-coding RNA biogenesis; RNA processing (mRNA splicing) |
| *MTBP* | *MDM2BP* | Y | N | an MDM2-binding protein that enhances MDM2-mediated p53 degradation (Boyd et al., 2000; Brady et al., 2005); knockdown reduces MAD1 and MAD2 kinetochore localization and triggers chromosome missegregation (Agarwal et al., 2011); via interaction with the DNA replication factor TICRR, regulates DNA replication initiation (Boos et al., 2013) | cell cycle (DNA replication/S phase, mitosis) |
| *NAA15* | *NATH, TBDN, NAT1P* | N | Y | an auxillary component of the N-terminal acetyltransferase A complex, which associates with the ribosome (Arnesen et al., 2005) | protein modification |
| *NAT10* | *ALP, NET43* | N | Y | N-acetyltransferase (Shen et al., 2009); direct role in decondensation of chromosomes at mitosis exit; knockdown results in prolonged chromosome condensation (Chi et al., 2007); DNA damage triggers increased amount of Nat10 in mitotic midbody, and results in enhanced acetylation of alpha-tubulin of midbody (Shen et al., 2009); knockdown results in abnormal nucleolus size, lengthened G2/M transition, multi-nucleated cells, and defects in cytokinesis, sometimes resulting in cell death (Shen et al., 2009); associates with U3 snoRNA and is required for 18S rRNA processing; knockdown results in decreased levels of 47S pre-rRNA, indicating that Nat10 is a transcriptional UTP (participates in pre-rRNA transcription) by targeting UBF for acetylation to facilitate association with RNA pol I-associated factor (Kong et al., 2011); 18S rRNA processing (Ito et al., 2014) | non-coding RNA biogenesis (rRNA); transcription (rRNA); cell cycle (mitosis/cytokinesis) |
| *NCBP1* | *CBP80, NCBP* | Y | Y | one of two proteins in the mRNA cap binding protein complex (other = CBP20), which regulates mRNA splicing (Izaurralde et al., 1994); nonsense mutation-containing transcripts are bound to Ncbp1 during nonsense-mediated decay (NMD) (Ishigaki et al., 2001); through interaction with TREX component Aly, allows… | RNA metabolism; RNA processing (mRNA splicing); RNA transport; translation (pioneer round) |
| **MIG** | **Syn** | **Core**  **Essent.?** | **Ancient**  **Essent.?** | **Function Description** | **Functional Categories** |
| *NCBP1* | *CBP80, NCBP* | Y | Y | … for proper mRNA export (Cheng et al., 2006); required for poly(A) RNA export (Gebhardt et al., 2015); as CBC, mediates translation initially for pioneer round, then replaced by eIF4E, which controls steady-state translation (Maquat et al., 2010); associates with Upf1 to promote nonsense-mediated decay (Hosoda et al., 2005; Hwang et al., 2010) | RNA metabolism; RNA processing (mRNA splicing); RNA transport; translation (pioneer round) |
| *NCBP2* | *CBP20, CBC2, NIP1, PIG55* | Y | Y | one of two proteins in the mRNA cap binding protein complex (other being NCBP1), which affects RNA stability, splicing, export (specifically U RNA export), and translation initiation by binding 5' end (Izaurralde et al., 1995); NCBP2 specifically binds the cap (Calero et al., 2002); also involved in processing of 3' end of the mRNA transcript (Flaherty et al., 1997); nonsense mutation-containing transcripts are bound to NCBP2 during NMB (Ishigaki et al., 2001); as CBC, mediated translation initially, then replaced by eIF4E, which controls steady-state translation (Kim et al., 2009) | RNA metabolism; RNA processing (mRNA splicing); RNA transport (snRNA); translation (pioneer round) |
| *NEDD1* | *TUBGCP7* | Y | Y | centrosomal protein that is required for γ-tubulin ring complex localization to the centrosome, with knockdown causing defects in centrosomal microtubule nucleation, aberrant mitotic spindles, and inhibition of centriole duplication (Haren et al., 2006) | cell cycle (mitosis) |
| *NOP2* | *P120, NOL1* | Y | Y | initially discovered as proliferation-associated based on expression, with moderately strong ribosome RNA methyl transferase activity (Freeman et al., 1988); introduction of antisense RNA limited proliferation in NIH 3T3 cells (Valdez et al., 1992); proliferation marker of neural stem cells, and is expressed in the adult brain (Kosi et al., 2015); potential role in neutrophil maturation (Khanna-Gupta et al., 2006) | cell cycle (proliferation) |
| *NUP205* | *NPHS13, C7orf14* | N | Y | a 205 kDa component of the nuclear pore complex that is required for long-term nuclear pore complex maintenance (Grandi et al., 1997; Krull et al., 2004); based on work in *C. elegans*, may inform nuclear pore complex permeability and distribution (Galy et al., 2003) | transport (nucleocytoplasmic) |
| **MIG** | **Syn** | **Core**  **Essent.?** | **Ancient**  **Essent.?** | **Function Description** | **Functional Categories** |
| *PDCD11* | *ALG4, NFBP, RRP5* | N | Y | interacts with the U3 snoRNA (involved in rRNA maturation/ biogenesis), and knockdown represses 18S rRNA maturation (Sweet et al., 2008); part of SSU processome, where it likely recruits U3 to sites of rRNA maturation (Turner et al., 2009) | non-coding RNA biogenesis (rRNA) |
| *POLA2* |  | N | Y | indirectly, regulates DNA replication by increasing protein synthesis and nuclear translocation of catalytic alpha subunit (p180) (Mizuno et al., 1999); hyperphosphorylated by cyclin-dependent kinases in G2 phase, which enhances activation of pol-alpha enzyme (DNA replication) by phosphorylated Rb (Takemura et al., 2006); constitutive knockout is embryonically lethal in mice (Dickinson et al., 2016) | cell cycle (DNA replication/S phase) |
| *POLE2* | *DPE2* | Y | Y | DNA polymerase accessory subunit (epsilon), 55 kDa subunit (Li et al., 1997); possible stabilizing role for DNA polymerase epsilon complex (Li et al., 1997); role in chromatin regulation, based on reporter plasmid assays (Wada et al., 2002) | cell cycle (DNA replication/S phase); transcription regulation |
| *POLR2E* | *RPB5, RPABC1, XAP4* | Y | Y | subunit shared by all RNA polymerases (Cheong et al., 1995); role in transcription activation, according to work in yeast (Miyao and Woychik, 1998); in COS1 cells, directly binds RAP30, component of the general transcription factor IIF (TFIIF) complex; this complex is assembled within the initiation complex and is known to associate with RNA pol II, inhibiting the association of TFIIF and pol II (Wei et al. 2001; Wei et al., 2003) | transcription |
| *POLR2K* | *RPB12, RPABC4, RPB10α, ABC10α* | N | Y | a small subunit of all three DNA-directed RNA polymerases (Shpakovski et al., 1995) | transcription; non-coding RNA biogenesis |
| *POLR3H* | *RPC8* | Y | Y | subunit of RNA polymerase III, acting paralogously to Rpb7 in pol II (Hu et al., 2002) | transcription; non-coding RNA biogenesis |
| *PPIL4* |  | N | Y | a member of the cyclophilin family of peptidyl-prolyl isomerases (Zeng et al., 2001; Wang and Heitman, 2005) | protein folding |
| **MIG** | **Syn** | **Core**  **Essent.?** | **Ancient**  **Essent.?** | **Function Description** | **Functional Categories** |
| *PRIM1* | p49 | Y | Y | catalytic primase subunit of DNA polymerase α-primase (Urban et al., 2010); generates an RNA primer, followed by primer extension to produce RNA-DNA primer, to initiate DNA replication (reviewed by Waga and Stillman, 1998) | cell cycle (DNA replication/S phase) |
| *PSMA1* | *PROS30* | Y | Y | component of 20S proteasome (Cron et al., 2013) | protein metabolism |
| *RABGGTA* | *PTAR3* | N | Y | geranylgeranyl transferase; mutation in splice acceptor site results in gunmetal mouse, which has platelet and megakaryocyte defects (prolonged bleeding), macrothrombocytopenia (Detter et al., 2000) | protein modification; hemostasis |
| *RNPC3* | *IGHD5, RBM40, RNP, SNRNP65* | Y | Y | the minor spliceosome-specific U11/U12 65k protein; when bound to the U11/U12 di-snRNP, it bridges the U12 snRNA and U11-59K protein, stabilizing the di-snRNP and thereby regulating minor splicing (Benecke et al., 2005); germline deletion in mice results in embryonic lethality (Doggett et al., 2018); constitutive deletion in adult mice results in lower levels of lymphocytes, monocytes, erythrocytes, and thrombocytes; decreased thymus size; gastrointestinal mucosa degeneration; and death within 8 days of induction (Doggett et al., 2018); in humans, mutation is linked to isolated familial growth hormone deficiency (Argente et al., 2014) | RNA processing (splicing); development |
| *RPL4* |  | Y | Y | ribosomal protein component of the 60S subunit (Chan et al., 1995) | translation |
| *RPP30* | *TSG15* | N | Y | 30 kDa subunit of the ribonuclease P/MRP complex, which processes tRNA (Jarrous et al., 1998) | non-coding RNA biogenesis |
| *SACM1L* | *SAC1* | N | Y | phosphoinositide phosphatase integral membrane protein localized to endoplasmic reticulum and Golgi apparatus (Nemoto et al., 2000; Rohde et al., 2003); mouse KO is early embryonic lethal (Liu et al., 2008); knockdown causes Golgi apparatus disorganization, delayed G2/M transition, and aberrant mitotic spindle formation (Liu et al., 2008) | cell cycle (mitosis); Golgi-mediated transport |
| **MIG** | **Syn** | **Core**  **Essent.?** | **Ancient**  **Essent.?** | **Function Description** | **Functional Categories** |
| *SBNO1* | *MOP3* | Y | Y | a putative member of the SF2-type DExD/H helicases that regulates transcription (Watanabe et al., 2017); in mice, required for preimplantation development (Watanabe et al., 2017) | transcription regulation |
| *SMC3* | *BAM, HCAP, CSPG6* | N | Y | an ATPase component of cohesin, mediating sister chromatid cohesion and metaphase progression (Schmiesing et al., 1998); mirroring cohesin's role in DNA repair and the DNA damage-induced intra-S and G2/M phase checkpoints, SMC3 is phosphorylated upon DNA damage (Watrin and Peters, 2009); also a subunit of the RC-1 complex, which functions in DNA recombination and repair (Stursberg et al., 1999) | cell cycle (mitosis); genome integrity (DNA repair) |
| *SNRPE* | *SME, Sm-E, HYPT11* | Y | Y | the "E" subunit of the Sm complex, which functions in snRNP maturation; associates with U snRNPs, including those involved in RNA splicing and histone mRNA processing (Pillai et al., 2001; Urlaub et al., 2001; Chari et al., 2008; Pasternack et al., 2013); in cancer cells, misexpression prevents DNA synthesis and arrests cells in G2, while knockdown drives cells through these phases (Li and Putzer, 2008) | RNA processing (splicing); transcription; cell cycle |
| *SPC24* | *SPBC24* | Y | N | NDC80 kinetochore complex component; required to establish and maintain kinetochore-microtubule attachment in mitosis (McCleland et al., 2004) | cell cycle (mitosis) |
| *SRP72* | *BMFF, BMFS1* | N | Y | the 72 kDa subunit of the signal recognition particle, which mediates co-translational protein targeting to the endoplasmic reticulum (Lutcke et al., 1993) | transport (protein, to ER); translation |
| *SSU72* | *HSPC182* | N | Y | phosphatase associated with CTD of RNA pol II (TFIIB) (St-Pierre et al., 2005); regulates RNA pol II activity via CTD phosphatase activity in yeast (Krishnamurthy et al., 2004); phosphorylated by Aurora B to regulate sister chromatid cohesion during mitosis (Kim et al., 2013) | transcription; cell cycle (mitosis) |
| *TAF1C* | *SL1, TAFI110, TAFI95* | N | N | TATA box-binding protein associated factor (TAF) for RNA pol I; part of SL1 complex, which directs pol I transcription and can independently interaction with rDNA promoters (Friedrich et al., 2005) | non-coding RNA biogenesis (rRNA) |
| **MIG** | **Syn** | **Core**  **Essent.?** | **Ancient**  **Essent.?** | **Function Description** | **Functional Categories** |
| *TCP1* | *CCT1, CCT-alpha, CCTa* | Y | Y | cytosolic chaperonin responsible for stabilizing folding of numerous proteins, including actin (Neal and Joyce, 1992) and tubulin (Yaffe et al., 1992; Yam et al., 2008); involved in ciliogenesis and biogenesis of rod outer segment (Sinha et al., 2014); via chaperone function for Tcab1, controls scaRNA localization and telomerase function (Freund et al., 2014) | protein folding |
| *TRAPPC8* | *GSG1, TRS85* | N | Y | a component of the TRAPP III complex, which functions in ER-to-Golgi transport in the early secretory pathway and in autophagy (Choi et al., 2011; Scrivens et al., 2011; Lamb et al., 2016) | transport (ER to Golgi); autophagy |
| *UBL5* | *HUB1* | Y | Y | ubiquitin-like protein; knock-down experiments indicate a role in stabilization of the spliceosome and in mitotic progression to anaphase (Oka et al., 2014); via this role, important for the proper splicing of the cohesin factor sororin, thereby indirectly promoting sister chromatid cohesion (Oka et al., 2014) | RNA processing (splicing); cell cycle (mitosis) |
| *UFD1L* | *UFD1* | N | Y | an adaptor protein component of the AAA-ATPase Cdc48/p97 complex that functions in endoplasmic reticulum-associated degradation (ERAD) (Chen et al., 2011); required for proper chromosome segregation and mitotic progression, due to role in Aurora B regulation (Dobrynin et al., 2011); mediates the G2/M checkpoint by targeting CDC25A upon DNA damage (Riemer et al., 2014); required for proper localization of the ESCRT-III complex to the nuclear envelope during nuclear envelope reformation during late mitosis (Olmos et al., 2015) | protein turnover; cell cycle (mitosis) |
| *UPF1* | *RENT1* | N | Y | functions in nonsense-mediated mRNA decay (Sun et al., 1998) and genome integrity (Azzalin and Lingner, 2006); constitutive knockout is embryonically lethal (Medghalchi et al., 2001) | RNA metabolism; genome integrity |
| *VPS25* | *DERP9, EAP20, FAP20* | Y | Y | part of ESCRT (endosomal sorting complex required for transport)-II (Yorikawa et al., 2005); role in cargo sorting, especially sorting of ubiquitinated cargo (Im et al., 2009); by regulating receptor number, affects FGF signaling in limbs, indicative of a role in skeletal/limb development (Handschuh et al., 2014); constitutive knockout is embryonically lethal (Dickinson et al., 2016) | transport; limb/skeletal development |
| **MIG** | **Syn** | **Core**  **Essent.?** | **Ancient**  **Essent.?** | **Function Description** | **Functional Categories** |
| *ZPR1* | *ZNF259, ZFP259* | N | Y | depletion disrupts nucleolar function, including pre-ribosomal RNA expression (Galcheva-Gargova et al., 1998); interacts with the Smn protein and colocalizes in Geminins and Cajal bodies; depletion analysis indicates Zfp259 regulates localization of Smn in nuclear bodies (Gangwani et al., 2001); in conditonal knockout mouse, loss of Zfp259 disrupts the subcellular localization of Smn and the spliceosomal snRNPs; early embryonic lethality is observed, showing reduced proliferation and increased apoptosis [378]; depletion blocks S-phase progression, triggers G1 and G2 arrest, and causes mislocalization of Smn and NPAT (Gangwani et al., 2001); in Zfp259-deficient mice, observe axonal pathology and neurodegeneration (Doran et al., 2006); in spinal muscular atrophy, loss of Zpr1 increases motor neuron loss and severity and depleted SMN-containing subnuclear bodies (Ahmad et al., 2012) | RNA processing (splicing); neuron function (survival) |

**Supplementary References**

Achsel, T., Brahms, H., Kastner, B., Bachi, A., Wilm, M., and Luhrmann, R. (1999). A doughnut-shaped heteromer of human Sm-like proteins binds to the 3'-end of U6 snRNA, thereby facilitating U4/U6 duplex formation in vitro. *EMBO J* 18(20)**,** 5789-5802. doi: 10.1093/emboj/18.20.5789.

Agarwal, N., Tochigi, Y., Adhikari, A.S., Cui, S., Cui, Y., and Iwakuma, T. (2011). MTBP plays a crucial role in mitotic progression and chromosome segregation. *Cell Death Differ* 18(7)**,** 1208-1219. doi: 10.1038/cdd.2010.189.

Ahlemann, M., Zeidler, R., Lang, S., Mack, B., Munz, M., and Gires, O. (2006). Carcinoma-associated eIF3i overexpression facilitates mTOR-dependent growth transformation. *Mol Carcinog* 45(12)**,** 957-967. doi: 10.1002/mc.20269.

Ahmad, S., Wang, Y., Shaik, G.M., Burghes, A.H., and Gangwani, L. (2012). The zinc finger protein ZPR1 is a potential modifier of spinal muscular atrophy. *Hum Mol Genet* 21(12)**,** 2745-2758. doi: 10.1093/hmg/dds102.

Antoshechkin, I., and Han, M. (2002). The C. elegans evl-20 gene is a homolog of the small GTPase ARL2 and regulates cytoskeleton dynamics during cytokinesis and morphogenesis. *Dev Cell* 2(5)**,** 579-591.

Argente, J., Flores, R., Gutierrez-Arumi, A., Verma, B., Martos-Moreno, G.A., Cusco, I., et al. (2014). Defective minor spliceosome mRNA processing results in isolated familial growth hormone deficiency. *EMBO Mol Med* 6(3)**,** 299-306. doi: 10.1002/emmm.201303573.

Arnesen, T., Anderson, D., Baldersheim, C., Lanotte, M., Varhaug, J.E., and Lillehaug, J.R. (2005). Identification and characterization of the human ARD1-NATH protein acetyltransferase complex. *Biochem J* 386(Pt 3)**,** 433-443. doi: 10.1042/BJ20041071.

Azzalin, C.M., and Lingner, J. (2006). The human RNA surveillance factor UPF1 is required for S phase progression and genome stability. *Curr Biol* 16(4)**,** 433-439. doi: 10.1016/j.cub.2006.01.018.

Baillat, D., Hakimi, M.A., Naar, A.M., Shilatifard, A., Cooch, N., and Shiekhattar, R. (2005). Integrator, a multiprotein mediator of small nuclear RNA processing, associates with the C-terminal repeat of RNA polymerase II. *Cell* 123(2)**,** 265-276. doi: 10.1016/j.cell.2005.08.019.

Bao, X., Tang, J., Lopez-Pajares, V., Tao, S., Qu, K., Crabtree, G.R., et al. (2013). ACTL6a enforces the epidermal progenitor state by suppressing SWI/SNF-dependent induction of KLF4. *Cell Stem Cell* 12(2)**,** 193-203. doi: 10.1016/j.stem.2012.12.014.

Benecke, H., Luhrmann, R., and Will, C.L. (2005). The U11/U12 snRNP 65K protein acts as a molecular bridge, binding the U12 snRNA and U11-59K protein. *EMBO J* 24(17)**,** 3057-3069. doi: 10.1038/sj.emboj.7600765.

Bertram, K., Agafonov, D.E., Dybkov, O., Haselbach, D., Leelaram, M.N., Will, C.L., et al. (2017a). Cryo-EM Structure of a Pre-catalytic Human Spliceosome Primed for Activation. *Cell* 170(4)**,** 701-713 e711. doi: 10.1016/j.cell.2017.07.011.

Bertram, K., Agafonov, D.E., Liu, W.T., Dybkov, O., Will, C.L., Hartmuth, K., et al. (2017b). Cryo-EM structure of a human spliceosome activated for step 2 of splicing. *Nature* 542(7641)**,** 318-323. doi: 10.1038/nature21079.

Boos, D., Yekezare, M., and Diffley, J.F. (2013). Identification of a heteromeric complex that promotes DNA replication origin firing in human cells. *Science* 340(6135)**,** 981-984. doi: 10.1126/science.1237448.

Boyd, M.T., Vlatkovic, N., and Haines, D.S. (2000). A novel cellular protein (MTBP) binds to MDM2 and induces a G1 arrest that is suppressed by MDM2. *J Biol Chem* 275(41)**,** 31883-31890. doi: 10.1074/jbc.M004252200.

Brady, M., Vlatkovic, N., and Boyd, M.T. (2005). Regulation of p53 and MDM2 activity by MTBP. *Mol Cell Biol* 25(2)**,** 545-553. doi: 10.1128/MCB.25.2.545-553.2005.

Calera, M.R., Zamora-Ramos, C., Araiza-Villanueva, M.G., Moreno-Aguilar, C.A., Pena-Gomez, S.G., Castellanos-Teran, F., et al. (2011). Parcs/Gpn3 is required for the nuclear accumulation of RNA polymerase II. *Biochim Biophys Acta* 1813(10)**,** 1708-1716. doi: 10.1016/j.bbamcr.2011.07.005.

Calero, G., Wilson, K.F., Ly, T., Rios-Steiner, J.L., Clardy, J.C., and Cerione, R.A. (2002). Structural basis of m7GpppG binding to the nuclear cap-binding protein complex. *Nat Struct Biol* 9(12)**,** 912-917. doi: 10.1038/nsb874.

Cang, Y., Zhang, J., Nicholas, S.A., Bastien, J., Li, B., Zhou, P., et al. (2006). Deletion of DDB1 in mouse brain and lens leads to p53-dependent elimination of proliferating cells. *Cell* 127(5)**,** 929-940. doi: 10.1016/j.cell.2006.09.045.

Carre, C., and Shiekhattar, R. (2011). Human GTPases associate with RNA polymerase II to mediate its nuclear import. *Mol Cell Biol* 31(19)**,** 3953-3962. doi: 10.1128/MCB.05442-11.

Chan, Y.L., Olvera, J., and Wool, I.G. (1995). The primary structures of rat ribosomal proteins L4 and L41. *Biochem Biophys Res Commun* 214(3)**,** 810-818. doi: 10.1006/bbrc.1995.2359.

Chari, A., Golas, M.M., Klingenhager, M., Neuenkirchen, N., Sander, B., Englbrecht, C., et al. (2008). An assembly chaperone collaborates with the SMN complex to generate spliceosomal SnRNPs. *Cell* 135(3)**,** 497-509. doi: 10.1016/j.cell.2008.09.020.

Chen, M., Gutierrez, G.J., and Ronai, Z.A. (2011). Ubiquitin-recognition protein Ufd1 couples the endoplasmic reticulum (ER) stress response to cell cycle control. *Proc Natl Acad Sci U S A* 108(22)**,** 9119-9124. doi: 10.1073/pnas.1100028108.

Cheng, H., Dufu, K., Lee, C.S., Hsu, J.L., Dias, A., and Reed, R. (2006). Human mRNA export machinery recruited to the 5' end of mRNA. *Cell* 127(7)**,** 1389-1400. doi: 10.1016/j.cell.2006.10.044.

Cheong, J.H., Yi, M., Lin, Y., and Murakami, S. (1995). Human RPB5, a subunit shared by eukaryotic nuclear RNA polymerases, binds human hepatitis B virus X protein and may play a role in X transactivation. *EMBO J* 14(1)**,** 143-150.

Chi, Y.H., Haller, K., Peloponese, J.M., Jr., and Jeang, K.T. (2007). Histone acetyltransferase hALP and nuclear membrane protein hsSUN1 function in de-condensation of mitotic chromosomes. *J Biol Chem* 282(37)**,** 27447-27458. doi: 10.1074/jbc.M703098200.

Choi, C., Davey, M., Schluter, C., Pandher, P., Fang, Y., Foster, L.J., et al. (2011). Organization and assembly of the TRAPPII complex. *Traffic* 12(6)**,** 715-725. doi: 10.1111/j.1600-0854.2011.01181.x.

Chung, S., Zhou, Z., Huddleston, K.A., Harrison, D.A., Reed, R., Coleman, T.A., et al. (2002). Crooked neck is a component of the human spliceosome and implicated in the splicing process. *Biochim Biophys Acta* 1576(3)**,** 287-297. doi: 10.1016/s0167-4781(02)00368-8.

Clever, M., Funakoshi, T., Mimura, Y., Takagi, M., and Imamoto, N. (2012). The nucleoporin ELYS/Mel28 regulates nuclear envelope subdomain formation in HeLa cells. *Nucleus* 3(2)**,** 187-199. doi: 10.4161/nucl.19595.

Cron, K.R., Zhu, K., Kushwaha, D.S., Hsieh, G., Merzon, D., Rameseder, J., et al. (2013). Proteasome inhibitors block DNA repair and radiosensitize non-small cell lung cancer. *PLoS One* 8(9)**,** e73710. doi: 10.1371/journal.pone.0073710.

Dalwadi, U., and Yip, C.K. (2018). Structural insights into the function of Elongator. *Cell Mol Life Sci* 75(9)**,** 1613-1622. doi: 10.1007/s00018-018-2747-6.

Detter, J.C., Zhang, Q., Mules, E.H., Novak, E.K., Mishra, V.S., Li, W., et al. (2000). Rab geranylgeranyl transferase alpha mutation in the gunmetal mouse reduces Rab prenylation and platelet synthesis. *Proc Natl Acad Sci U S A* 97(8)**,** 4144-4149. doi: 10.1073/pnas.080517697.

Dhar, S.K., Delmolino, L., and Dutta, A. (2001). Architecture of the human origin recognition complex. *J Biol Chem* 276(31)**,** 29067-29071. doi: 10.1074/jbc.M103078200.

Dickinson, M.E., Flenniken, A.M., Ji, X., Teboul, L., Wong, M.D., White, J.K., et al. (2016). High-throughput discovery of novel developmental phenotypes. *Nature* 537(7621)**,** 508-514. doi: 10.1038/nature19356.

Dobrynin, G., Popp, O., Romer, T., Bremer, S., Schmitz, M.H., Gerlich, D.W., et al. (2011). Cdc48/p97-Ufd1-Npl4 antagonizes Aurora B during chromosome segregation in HeLa cells. *J Cell Sci* 124(Pt 9)**,** 1571-1580. doi: 10.1242/jcs.069500.

Doggett, K., Williams, B.B., Markmiller, S., Geng, F.S., Coates, J., Mieruszynski, S., et al. (2018). Early developmental arrest and impaired gastrointestinal homeostasis in U12-dependent splicing-defective Rnpc3-deficient mice. *RNA* 24(12)**,** 1856-1870. doi: 10.1261/rna.068221.118.

Dong, L., Wen, J., Pier, E., Zhang, X., Zhang, B., Dong, F., et al. (2010). Melanocyte-stimulating hormone directly enhances UV-Induced DNA repair in keratinocytes by a xeroderma pigmentosum group A-dependent mechanism. *Cancer Res* 70(9)**,** 3547-3556. doi: 10.1158/0008-5472.CAN-09-4596.

Doran, B., Gherbesi, N., Hendricks, G., Flavell, R.A., Davis, R.J., and Gangwani, L. (2006). Deficiency of the zinc finger protein ZPR1 causes neurodegeneration. *Proc Natl Acad Sci U S A* 103(19)**,** 7471-7475. doi: 10.1073/pnas.0602057103.

Eckley, D.M., Gill, S.R., Melkonian, K.A., Bingham, J.B., Goodson, H.V., Heuser, J.E., et al. (1999). Analysis of dynactin subcomplexes reveals a novel actin-related protein associated with the arp1 minifilament pointed end. *J Cell Biol* 147(2)**,** 307-320. doi: 10.1083/jcb.147.2.307.

Evrony, G.D., Cordero, D.R., Shen, J., Partlow, J.N., Yu, T.W., Rodin, R.E., et al. (2017). Integrated genome and transcriptome sequencing identifies a noncoding mutation in the genome replication factor DONSON as the cause of microcephaly-micromelia syndrome. *Genome Res* 27(8)**,** 1323-1335. doi: 10.1101/gr.219899.116.

Flaherty, S.M., Fortes, P., Izaurralde, E., Mattaj, I.W., and Gilmartin, G.M. (1997). Participation of the nuclear cap binding complex in pre-mRNA 3' processing. *Proc Natl Acad Sci U S A* 94(22)**,** 11893-11898.

Forget, D., Lacombe, A.A., Cloutier, P., Al-Khoury, R., Bouchard, A., Lavallee-Adam, M., et al. (2010). The protein interaction network of the human transcription machinery reveals a role for the conserved GTPase RPAP4/GPN1 and microtubule assembly in nuclear import and biogenesis of RNA polymerase II. *Mol Cell Proteomics* 9(12)**,** 2827-2839. doi: 10.1074/mcp.M110.003616.

Freeman, J.W., Busch, R.K., Gyorkey, F., Gyorkey, P., Ross, B.E., and Busch, H. (1988). Identification and characterization of a human proliferation-associated nucleolar antigen with a molecular weight of 120,000 expressed in early G1 phase. *Cancer Res* 48(5)**,** 1244-1251.

Freund, A., Zhong, F.L., Venteicher, A.S., Meng, Z., Veenstra, T.D., Frydman, J., et al. (2014). Proteostatic control of telomerase function through TRiC-mediated folding of TCAB1. *Cell* 159(6)**,** 1389-1403. doi: 10.1016/j.cell.2014.10.059.

Friedrich, J.K., Panov, K.I., Cabart, P., Russell, J., and Zomerdijk, J.C. (2005). TBP-TAF complex SL1 directs RNA polymerase I pre-initiation complex formation and stabilizes upstream binding factor at the rDNA promoter. *J Biol Chem* 280(33)**,** 29551-29558. doi: 10.1074/jbc.M501595200.

Galcheva-Gargova, Z., Gangwani, L., Konstantinov, K.N., Mikrut, M., Theroux, S.J., Enoch, T., et al. (1998). The cytoplasmic zinc finger protein ZPR1 accumulates in the nucleolus of proliferating cells. *Mol Biol Cell* 9(10)**,** 2963-2971.

Galy, V., Mattaj, I.W., and Askjaer, P. (2003). Caenorhabditis elegans nucleoporins Nup93 and Nup205 determine the limit of nuclear pore complex size exclusion in vivo. *Mol Biol Cell* 14(12)**,** 5104-5115. doi: 10.1091/mbc.e03-04-0237.

Gangwani, L., Mikrut, M., Theroux, S., Sharma, M., and Davis, R.J. (2001). Spinal muscular atrophy disrupts the interaction of ZPR1 with the SMN protein. *Nat Cell Biol* 3(4)**,** 376-383. doi: 10.1038/35070059.

Gebhardt, A., Habjan, M., Benda, C., Meiler, A., Haas, D.A., Hein, M.Y., et al. (2015). mRNA export through an additional cap-binding complex consisting of NCBP1 and NCBP3. *Nat Commun* 6**,** 8192. doi: 10.1038/ncomms9192.

Gerhardt, J., Guler, G.D., and Fanning, E. (2015). Human DNA helicase B interacts with the replication initiation protein Cdc45 and facilitates Cdc45 binding onto chromatin. *Exp Cell Res* 334(2)**,** 283-293. doi: 10.1016/j.yexcr.2015.04.014.

Grandi, P., Dang, T., Pane, N., Shevchenko, A., Mann, M., Forbes, D., et al. (1997). Nup93, a vertebrate homologue of yeast Nic96p, forms a complex with a novel 205-kDa protein and is required for correct nuclear pore assembly. *Mol Biol Cell* 8(10)**,** 2017-2038. doi: 10.1091/mbc.8.10.2017.

Handschuh, K., Feenstra, J., Koss, M., Ferretti, E., Risolino, M., Zewdu, R., et al. (2014). ESCRT-II/Vps25 constrains digit number by endosome-mediated selective modulation of FGF-SHH signaling. *Cell Rep* 9(2)**,** 674-687. doi: 10.1016/j.celrep.2014.09.019.

Haren, L., Remy, M.H., Bazin, I., Callebaut, I., Wright, M., and Merdes, A. (2006). NEDD1-dependent recruitment of the gamma-tubulin ring complex to the centrosome is necessary for centriole duplication and spindle assembly. *J Cell Biol* 172(4)**,** 505-515. doi: 10.1083/jcb.200510028.

Harris, T.E., Chi, A., Shabanowitz, J., Hunt, D.F., Rhoads, R.E., and Lawrence, J.C., Jr. (2006). mTOR-dependent stimulation of the association of eIF4G and eIF3 by insulin. *EMBO J* 25(8)**,** 1659-1668. doi: 10.1038/sj.emboj.7601047.

Hashimoto, M., Sato, T., Muroyama, Y., Fujimura, L., Hatano, M., and Saito, T. (2015). Nepro is localized in the nucleolus and essential for preimplantation development in mice. *Dev Growth Differ* 57(7)**,** 529-538. doi: 10.1111/dgd.12232.

Hawkes, N.A., Otero, G., Winkler, G.S., Marshall, N., Dahmus, M.E., Krappmann, D., et al. (2002). Purification and characterization of the human elongator complex. *J Biol Chem* 277(4)**,** 3047-3052. doi: 10.1074/jbc.M110445200.

Hosoda, N., Kim, Y.K., Lejeune, F., and Maquat, L.E. (2005). CBP80 promotes interaction of Upf1 with Upf2 during nonsense-mediated mRNA decay in mammalian cells. *Nat Struct Mol Biol* 12(10)**,** 893-901. doi: 10.1038/nsmb995.

Hu, P., Wu, S., Sun, Y., Yuan, C.C., Kobayashi, R., Myers, M.P., et al. (2002). Characterization of human RNA polymerase III identifies orthologues for Saccharomyces cerevisiae RNA polymerase III subunits. *Mol Cell Biol* 22(22)**,** 8044-8055.

Huang, B., Johansson, M.J., and Bystrom, A.S. (2005). An early step in wobble uridine tRNA modification requires the Elongator complex. *RNA* 11(4)**,** 424-436. doi: 10.1261/rna.7247705.

Hwang, J., Sato, H., Tang, Y., Matsuda, D., and Maquat, L.E. (2010). UPF1 association with the cap-binding protein, CBP80, promotes nonsense-mediated mRNA decay at two distinct steps. *Mol Cell* 39(3)**,** 396-409. doi: 10.1016/j.molcel.2010.07.004.

Im, Y.J., Wollert, T., Boura, E., and Hurley, J.H. (2009). Structure and function of the ESCRT-II-III interface in multivesicular body biogenesis. *Dev Cell* 17(2)**,** 234-243. doi: 10.1016/j.devcel.2009.07.008.

Ishigaki, Y., Li, X., Serin, G., and Maquat, L.E. (2001). Evidence for a pioneer round of mRNA translation: mRNAs subject to nonsense-mediated decay in mammalian cells are bound by CBP80 and CBP20. *Cell* 106(5)**,** 607-617.

Isken, O., Kim, Y.K., Hosoda, N., Mayeur, G.L., Hershey, J.W., and Maquat, L.E. (2008). Upf1 phosphorylation triggers translational repression during nonsense-mediated mRNA decay. *Cell* 133(2)**,** 314-327. doi: 10.1016/j.cell.2008.02.030.

Ito, S., Horikawa, S., Suzuki, T., Kawauchi, H., Tanaka, Y., Suzuki, T., et al. (2014). Human NAT10 is an ATP-dependent RNA acetyltransferase responsible for N4-acetylcytidine formation in 18 S ribosomal RNA (rRNA). *J Biol Chem* 289(52)**,** 35724-35730. doi: 10.1074/jbc.C114.602698.

Ito, S., Tan, L.J., Andoh, D., Narita, T., Seki, M., Hirano, Y., et al. (2010). MMXD, a TFIIH-independent XPD-MMS19 protein complex involved in chromosome segregation. *Mol Cell* 39(4)**,** 632-640. doi: 10.1016/j.molcel.2010.07.029.

Izaurralde, E., Lewis, J., Gamberi, C., Jarmolowski, A., McGuigan, C., and Mattaj, I.W. (1995). A cap-binding protein complex mediating U snRNA export. *Nature* 376(6542)**,** 709-712. doi: 10.1038/376709a0.

Izaurralde, E., Lewis, J., McGuigan, C., Jankowska, M., Darzynkiewicz, E., and Mattaj, I.W. (1994). A nuclear cap binding protein complex involved in pre-mRNA splicing. *Cell* 78(4)**,** 657-668.

Jarrous, N., Eder, P.S., Guerrier-Takada, C., Hoog, C., and Altman, S. (1998). Autoantigenic properties of some protein subunits of catalytically active complexes of human ribonuclease P. *RNA* 4(4)**,** 407-417.

Kanno, Y., Serikawa, T., Inajima, J., and Inouye, Y. (2012). DP97, a DEAD box DNA/RNA helicase, is a target gene-selective co-regulator of the constitutive androstane receptor. *Biochem Biophys Res Commun* 426(1)**,** 38-42. doi: 10.1016/j.bbrc.2012.08.027.

Khanna-Gupta, A., Sun, H., Zibello, T., Lozovatsky, L., Ghosh, P.K., Link, D.C., et al. (2006). p120 nucleolar-proliferating antigen is a direct target of G-CSF signaling during myeloid differentiation. *J Leukoc Biol* 79(5)**,** 1011-1021. doi: 10.1189/jlb.0205066.

Kim, H.S., Kim, S.H., Park, H.Y., Lee, J., Yoon, J.H., Choi, S., et al. (2013). Functional interplay between Aurora B kinase and Ssu72 phosphatase regulates sister chromatid cohesion. *Nat Commun* 4**,** 2631. doi: 10.1038/ncomms3631.

Kim, J.H., Lane, W.S., and Reinberg, D. (2002). Human Elongator facilitates RNA polymerase II transcription through chromatin. *Proc Natl Acad Sci U S A* 99(3)**,** 1241-1246. doi: 10.1073/pnas.251672198.

Kim, K.M., Cho, H., Choi, K., Kim, J., Kim, B.W., Ko, Y.G., et al. (2009). A new MIF4G domain-containing protein, CTIF, directs nuclear cap-binding protein CBP80/20-dependent translation. *Genes Dev* 23(17)**,** 2033-2045. doi: 10.1101/gad.1823409.

Kong, R., Zhang, L., Hu, L., Peng, Q., Han, W., Du, X., et al. (2011). hALP, a novel transcriptional U three protein (t-UTP), activates RNA polymerase I transcription by binding and acetylating the upstream binding factor (UBF). *J Biol Chem* 286(9)**,** 7139-7148. doi: 10.1074/jbc.M110.173393.

Kosi, N., Alic, I., Kolacevic, M., Vrsaljko, N., Jovanov Milosevic, N., Sobol, M., et al. (2015). Nop2 is expressed during proliferation of neural stem cells and in adult mouse and human brain. *Brain Res* 1597**,** 65-76. doi: 10.1016/j.brainres.2014.11.040.

Krasteva, V., Buscarlet, M., Diaz-Tellez, A., Bernard, M.A., Crabtree, G.R., and Lessard, J.A. (2012). The BAF53a subunit of SWI/SNF-like BAF complexes is essential for hemopoietic stem cell function. *Blood* 120(24)**,** 4720-4732. doi: 10.1182/blood-2012-04-427047.

Krief, P., Augery-Bourget, Y., Plaisance, S., Merck, M.F., Assier, E., Tanchou, V., et al. (1994). A new cytokine (IK) down-regulating HLA class II: monoclonal antibodies, cloning and chromosome localization. *Oncogene* 9(12)**,** 3449-3456.

Krishnamurthy, S., He, X., Reyes-Reyes, M., Moore, C., and Hampsey, M. (2004). Ssu72 Is an RNA polymerase II CTD phosphatase. *Mol Cell* 14(3)**,** 387-394.

Krull, S., Thyberg, J., Bjorkroth, B., Rackwitz, H.R., and Cordes, V.C. (2004). Nucleoporins as components of the nuclear pore complex core structure and Tpr as the architectural element of the nuclear basket. *Mol Biol Cell* 15(9)**,** 4261-4277. doi: 10.1091/mbc.e04-03-0165.

Kufel, J., Allmang, C., Petfalski, E., Beggs, J., and Tollervey, D. (2003). Lsm Proteins are required for normal processing and stability of ribosomal RNAs. *J Biol Chem* 278(4)**,** 2147-2156. doi: 10.1074/jbc.M208856200.

Kufel, J., Allmang, C., Verdone, L., Beggs, J.D., and Tollervey, D. (2002). Lsm proteins are required for normal processing of pre-tRNAs and their efficient association with La-homologous protein Lhp1p. *Mol Cell Biol* 22(14)**,** 5248-5256. doi: 10.1128/mcb.22.14.5248-5256.2002.

Kukimoto, I., Igaki, H., and Kanda, T. (1999). Human CDC45 protein binds to minichromosome maintenance 7 protein and the p70 subunit of DNA polymerase alpha. *Eur J Biochem* 265(3)**,** 936-943. doi: 10.1046/j.1432-1327.1999.00791.x.

Lamb, C.A., Nuhlen, S., Judith, D., Frith, D., Snijders, A.P., Behrends, C., et al. (2016). TBC1D14 regulates autophagy via the TRAPP complex and ATG9 traffic. *EMBO J* 35(3)**,** 281-301. doi: 10.15252/embj.201592695.

Larsen, D.H., Poinsignon, C., Gudjonsson, T., Dinant, C., Payne, M.R., Hari, F.J., et al. (2010). The chromatin-remodeling factor CHD4 coordinates signaling and repair after DNA damage. *J Cell Biol* 190(5)**,** 731-740. doi: 10.1083/jcb.200912135.

Lee, S., Han, S., Jeong, A.L., Park, J.S., and Yang, Y. (2014). Depletion of IK causes mitotic arrest through aberrant regulation of mitotic kinases and phosphatases. *FEBS Lett* 588(17)**,** 2844-2850. doi: 10.1016/j.febslet.2014.06.046.

Lee, S., Jeong, A.L., Park, J.S., Han, S., Jang, C.Y., Kim, K.I., et al. (2016). IK-guided PP2A suppresses Aurora B activity in the interphase of tumor cells. *Cell Mol Life Sci* 73(17)**,** 3375-3386. doi: 10.1007/s00018-016-2162-9.

Lejeune, F., Li, X., and Maquat, L.E. (2003). Nonsense-mediated mRNA decay in mammalian cells involves decapping, deadenylating, and exonucleolytic activities. *Mol Cell* 12(3)**,** 675-687.

Lessard, J., Wu, J.I., Ranish, J.A., Wan, M., Winslow, M.M., Staahl, B.T., et al. (2007). An essential switch in subunit composition of a chromatin remodeling complex during neural development. *Neuron* 55(2)**,** 201-215. doi: 10.1016/j.neuron.2007.06.019.

Leung-Pineda, V., Huh, J., and Piwnica-Worms, H. (2009). DDB1 targets Chk1 to the Cul4 E3 ligase complex in normal cycling cells and in cells experiencing replication stress. *Cancer Res* 69(6)**,** 2630-2637. doi: 10.1158/0008-5472.CAN-08-3382.

Li, J., Wang, Q.E., Zhu, Q., El-Mahdy, M.A., Wani, G., Praetorius-Ibba, M., et al. (2006). DNA damage binding protein component DDB1 participates in nucleotide excision repair through DDB2 DNA-binding and cullin 4A ubiquitin ligase activity. *Cancer Res* 66(17)**,** 8590-8597. doi: 10.1158/0008-5472.CAN-06-1115.

Li, Y., Asahara, H., Patel, V.S., Zhou, S., and Linn, S. (1997). Purification, cDNA cloning, and gene mapping of the small subunit of human DNA polymerase epsilon. *J Biol Chem* 272(51)**,** 32337-32344.

Li, Z., and Putzer, B.M. (2008). Spliceosomal protein E regulates neoplastic cell growth by modulating expression of cyclin E/CDK2 and G2/M checkpoint proteins. *J Cell Mol Med* 12(6A)**,** 2427-2438. doi: 10.1111/j.1582-4934.2008.00244.x.

Liu, S.S., Zheng, H.X., Jiang, H.D., He, J., Yu, Y., Qu, Y.P., et al. (2012). Identification and characterization of a novel gene, c1orf109, encoding a CK2 substrate that is involved in cancer cell proliferation. *J Biomed Sci* 19**,** 49. doi: 10.1186/1423-0127-19-49.

Liu, Y., Boukhelifa, M., Tribble, E., Morin-Kensicki, E., Uetrecht, A., Bear, J.E., et al. (2008). The Sac1 phosphoinositide phosphatase regulates Golgi membrane morphology and mitotic spindle organization in mammals. *Mol Biol Cell* 19(7)**,** 3080-3096. doi: 10.1091/mbc.E07-12-1290.

Lutcke, H., Prehn, S., Ashford, A.J., Remus, M., Frank, R., and Dobberstein, B. (1993). Assembly of the 68- and 72-kD proteins of signal recognition particle with 7S RNA. *J Cell Biol* 121(5)**,** 977-985. doi: 10.1083/jcb.121.5.977.

Maquat, L.E., Hwang, J., Sato, H., and Tang, Y. (2010). CBP80-promoted mRNP rearrangements during the pioneer round of translation, nonsense-mediated mRNA decay, and thereafter. *Cold Spring Harb Symp Quant Biol* 75**,** 127-134. doi: 10.1101/sqb.2010.75.028.

Masutani, M., Sonenberg, N., Yokoyama, S., and Imataka, H. (2007). Reconstitution reveals the functional core of mammalian eIF3. *EMBO J* 26(14)**,** 3373-3383. doi: 10.1038/sj.emboj.7601765.

McCleland, M.L., Kallio, M.J., Barrett-Wilt, G.A., Kestner, C.A., Shabanowitz, J., Hunt, D.F., et al. (2004). The vertebrate Ndc80 complex contains Spc24 and Spc25 homologs, which are required to establish and maintain kinetochore-microtubule attachment. *Curr Biol* 14(2)**,** 131-137.

Medghalchi, S.M., Frischmeyer, P.A., Mendell, J.T., Kelly, A.G., Lawler, A.M., and Dietz, H.C. (2001). Rent1, a trans-effector of nonsense-mediated mRNA decay, is essential for mammalian embryonic viability. *Hum Mol Genet* 10(2)**,** 99-105.

Mehlgarten, C., Jablonowski, D., Wrackmeyer, U., Tschitschmann, S., Sondermann, D., Jager, G., et al. (2010). Elongator function in tRNA wobble uridine modification is conserved between yeast and plants. *Mol Microbiol* 76(5)**,** 1082-1094. doi: 10.1111/j.1365-2958.2010.07163.x.

Metz-Estrella, D., Jonason, J.H., Sheu, T.J., Mroczek-Johnston, R.M., and Puzas, J.E. (2012). TRIP-1: a regulator of osteoblast function. *J Bone Miner Res* 27(7)**,** 1576-1584. doi: 10.1002/jbmr.1611.

Milek, M., Imami, K., Mukherjee, N., Bortoli, F., Zinnall, U., Hazapis, O., et al. (2017). DDX54 regulates transcriptome dynamics during DNA damage response. *Genome Res* 27(8)**,** 1344-1359. doi: 10.1101/gr.218438.116.

Minaker, S.W., Filiatrault, M.C., Ben-Aroya, S., Hieter, P., and Stirling, P.C. (2013). Biogenesis of RNA polymerases II and III requires the conserved GPN small GTPases in Saccharomyces cerevisiae. *Genetics* 193(3)**,** 853-864. doi: 10.1534/genetics.112.148726.

Mitchell, P., Petfalski, E., and Tollervey, D. (1996). The 3' end of yeast 5.8S rRNA is generated by an exonuclease processing mechanism. *Genes Dev* 10(4)**,** 502-513.

Miyao, T., and Woychik, N.A. (1998). RNA polymerase subunit RPB5 plays a role in transcriptional activation. *Proc Natl Acad Sci U S A* 95(26)**,** 15281-15286.

Mizuno, T., Yamagishi, K., Miyazawa, H., and Hanaoka, F. (1999). Molecular architecture of the mouse DNA polymerase alpha-primase complex. *Mol Cell Biol* 19(11)**,** 7886-7896.

Motley, W.W., Talbot, K., and Fischbeck, K.H. (2010). GARS axonopathy: not every neuron's cup of tRNA. *Trends Neurosci* 33(2)**,** 59-66. doi: 10.1016/j.tins.2009.11.001.

Muroyama, Y., and Saito, T. (2009). Identification of Nepro, a gene required for the maintenance of neocortex neural progenitor cells downstream of Notch. *Development* 136(23)**,** 3889-3893. doi: 10.1242/dev.039180.

Neal, B.S., and Joyce, J.N. (1992). Neonatal 6-OHDA lesions differentially affect striatal D1 and D2 receptors. *Synapse* 11(1)**,** 35-46. doi: 10.1002/syn.890110106.

Nemoto, Y., Kearns, B.G., Wenk, M.R., Chen, H., Mori, K., Alb, J.G., Jr., et al. (2000). Functional characterization of a mammalian Sac1 and mutants exhibiting substrate-specific defects in phosphoinositide phosphatase activity. *J Biol Chem* 275(44)**,** 34293-34305. doi: 10.1074/jbc.M003923200.

Newman, L.E., Zhou, C.J., Mudigonda, S., Mattheyses, A.L., Paradies, E., Marobbio, C.M., et al. (2014). The ARL2 GTPase is required for mitochondrial morphology, motility, and maintenance of ATP levels. *PLoS One* 9(6)**,** e99270. doi: 10.1371/journal.pone.0099270.

Novotny, I., Podolska, K., Blazikova, M., Valasek, L.S., Svoboda, P., and Stanek, D. (2012). Nuclear LSm8 affects number of cytoplasmic processing bodies via controlling cellular distribution of Like-Sm proteins. *Mol Biol Cell* 23(19)**,** 3776-3785. doi: 10.1091/mbc.E12-02-0085.

Oka, Y., Varmark, H., Vitting-Seerup, K., Beli, P., Waage, J., Hakobyan, A., et al. (2014). UBL5 is essential for pre-mRNA splicing and sister chromatid cohesion in human cells. *EMBO Rep* 15(9)**,** 956-964. doi: 10.15252/embr.201438679.

Okita, K., Kiyonari, H., Nobuhisa, I., Kimura, N., Aizawa, S., and Taga, T. (2004). Targeted disruption of the mouse ELYS gene results in embryonic death at peri-implantation development. *Genes Cells* 9(11)**,** 1083-1091. doi: 10.1111/j.1365-2443.2004.00791.x.

Olmos, Y., Hodgson, L., Mantell, J., Verkade, P., and Carlton, J.G. (2015). ESCRT-III controls nuclear envelope reformation. *Nature* 522(7555)**,** 236-239. doi: 10.1038/nature14503.

Pan, M.R., Hsieh, H.J., Dai, H., Hung, W.C., Li, K., Peng, G., et al. (2012). Chromodomain helicase DNA-binding protein 4 (CHD4) regulates homologous recombination DNA repair, and its deficiency sensitizes cells to poly(ADP-ribose) polymerase (PARP) inhibitor treatment. *J Biol Chem* 287(9)**,** 6764-6772. doi: 10.1074/jbc.M111.287037.

Pasternack, S.M., Refke, M., Paknia, E., Hennies, H.C., Franz, T., Schafer, N., et al. (2013). Mutations in SNRPE, which encodes a core protein of the spliceosome, cause autosomal-dominant hypotrichosis simplex. *Am J Hum Genet* 92(1)**,** 81-87. doi: 10.1016/j.ajhg.2012.10.022.

Pillai, R.S., Will, C.L., Luhrmann, R., Schumperli, D., and Muller, B. (2001). Purified U7 snRNPs lack the Sm proteins D1 and D2 but contain Lsm10, a new 14 kDa Sm D1-like protein. *EMBO J* 20(19)**,** 5470-5479. doi: 10.1093/emboj/20.19.5470.

Pinto, S., Quintana, D.G., Smith, P., Mihalek, R.M., Hou, Z.H., Boynton, S., et al. (1999). latheo encodes a subunit of the origin recognition complex and disrupts neuronal proliferation and adult olfactory memory when mutant. *Neuron* 23(1)**,** 45-54.

Polo, S.E., Kaidi, A., Baskcomb, L., Galanty, Y., and Jackson, S.P. (2010). Regulation of DNA-damage responses and cell-cycle progression by the chromatin remodelling factor CHD4. *EMBO J* 29(18)**,** 3130-3139. doi: 10.1038/emboj.2010.188.

Price, H.P., Peltan, A., Stark, M., and Smith, D.F. (2010). The small GTPase ARL2 is required for cytokinesis in Trypanosoma brucei. *Mol Biochem Parasitol* 173(2)**,** 123-131.

Qi, J., Dong, Z., Liu, J., and Zhang, J.T. (2014). EIF3i promotes colon oncogenesis by regulating COX-2 protein synthesis and beta-catenin activation. *Oncogene* 33(32)**,** 4156-4163. doi: 10.1038/onc.2013.397.

Raineri, I., Wegmueller, D., Gross, B., Certa, U., and Moroni, C. (2004). Roles of AUF1 isoforms, HuR and BRF1 in ARE-dependent mRNA turnover studied by RNA interference. *Nucleic Acids Res* 32(4)**,** 1279-1288. doi: 10.1093/nar/gkh282.

Rajendran, R.R., Nye, A.C., Frasor, J., Balsara, R.D., Martini, P.G., and Katzenellenbogen, B.S. (2003). Regulation of nuclear receptor transcriptional activity by a novel DEAD box RNA helicase (DP97). *J Biol Chem* 278(7)**,** 4628-4638. doi: 10.1074/jbc.M210066200.

Rasala, B.A., Orjalo, A.V., Shen, Z., Briggs, S., and Forbes, D.J. (2006). ELYS is a dual nucleoporin/kinetochore protein required for nuclear pore assembly and proper cell division. *Proc Natl Acad Sci U S A* 103(47)**,** 17801-17806. doi: 10.1073/pnas.0608484103.

Reynolds, J.J., Bicknell, L.S., Carroll, P., Higgs, M.R., Shaheen, R., Murray, J.E., et al. (2017). Mutations in DONSON disrupt replication fork stability and cause microcephalic dwarfism. *Nat Genet* 49(4)**,** 537-549. doi: 10.1038/ng.3790.

Riemer, A., Dobrynin, G., Dressler, A., Bremer, S., Soni, A., Iliakis, G., et al. (2014). The p97-Ufd1-Npl4 ATPase complex ensures robustness of the G2/M checkpoint by facilitating CDC25A degradation. *Cell Cycle* 13(6)**,** 919-927. doi: 10.4161/cc.27779.

Rohde, H.M., Cheong, F.Y., Konrad, G., Paiha, K., Mayinger, P., and Boehmelt, G. (2003). The human phosphatidylinositol phosphatase SAC1 interacts with the coatomer I complex. *J Biol Chem* 278(52)**,** 52689-52699. doi: 10.1074/jbc.M307983200.

Sauvageau, E., Rochdi, M.D., Oueslati, M., Hamdan, F.F., Percherancier, Y., Simpson, J.C., et al. (2014). CNIH4 interacts with newly synthesized GPCR and controls their export from the endoplasmic reticulum. *Traffic* 15(4)**,** 383-400. doi: 10.1111/tra.12148.

Schmiesing, J.A., Ball, A.R., Jr., Gregson, H.C., Alderton, J.M., Zhou, S., and Yokomori, K. (1998). Identification of two distinct human SMC protein complexes involved in mitotic chromosome dynamics. *Proc Natl Acad Sci U S A* 95(22)**,** 12906-12911. doi: 10.1073/pnas.95.22.12906.

Scrivens, P.J., Noueihed, B., Shahrzad, N., Hul, S., Brunet, S., and Sacher, M. (2011). C4orf41 and TTC-15 are mammalian TRAPP components with a role at an early stage in ER-to-Golgi trafficking. *Mol Biol Cell* 22(12)**,** 2083-2093. doi: 10.1091/mbc.E10-11-0873.

Seburn, K.L., Nangle, L.A., Cox, G.A., Schimmel, P., and Burgess, R.W. (2006). An active dominant mutation of glycyl-tRNA synthetase causes neuropathy in a Charcot-Marie-Tooth 2D mouse model. *Neuron* 51(6)**,** 715-726. doi: 10.1016/j.neuron.2006.08.027.

Selvadurai, K., Wang, P., Seimetz, J., and Huang, R.H. (2014). Archaeal Elp3 catalyzes tRNA wobble uridine modification at C5 via a radical mechanism. *Nat Chem Biol* 10(10)**,** 810-812. doi: 10.1038/nchembio.1610.

Shen, Q., Zheng, X., McNutt, M.A., Guang, L., Sun, Y., Wang, J., et al. (2009). NAT10, a nucleolar protein, localizes to the midbody and regulates cytokinesis and acetylation of microtubules. *Exp Cell Res* 315(10)**,** 1653-1667. doi: 10.1016/j.yexcr.2009.03.007.

Shpakovski, G.V., Acker, J., Wintzerith, M., Lacroix, J.F., Thuriaux, P., and Vigneron, M. (1995). Four subunits that are shared by the three classes of RNA polymerase are functionally interchangeable between Homo sapiens and Saccharomyces cerevisiae. *Mol Cell Biol* 15(9)**,** 4702-4710. doi: 10.1128/mcb.15.9.4702.

Sinha, S., Belcastro, M., Datta, P., Seo, S., and Sokolov, M. (2014). Essential role of the chaperonin CCT in rod outer segment biogenesis. *Invest Ophthalmol Vis Sci* 55(6)**,** 3775-3785. doi: 10.1167/iovs.14-13889.

St-Pierre, B., Liu, X., Kha, L.C., Zhu, X., Ryan, O., Jiang, Z., et al. (2005). Conserved and specific functions of mammalian ssu72. *Nucleic Acids Res* 33(2)**,** 464-477. doi: 10.1093/nar/gki171.

Stehling, O., Mascarenhas, J., Vashisht, A.A., Sheftel, A.D., Niggemeyer, B., Rosser, R., et al. (2013). Human CIA2A-FAM96A and CIA2B-FAM96B integrate iron homeostasis and maturation of different subsets of cytosolic-nuclear iron-sulfur proteins. *Cell Metab* 18(2)**,** 187-198. doi: 10.1016/j.cmet.2013.06.015.

Stoecklin, G., Colombi, M., Raineri, I., Leuenberger, S., Mallaun, M., Schmidlin, M., et al. (2002). Functional cloning of BRF1, a regulator of ARE-dependent mRNA turnover. *EMBO J* 21(17)**,** 4709-4718. doi: 10.1093/emboj/cdf444.

Stursberg, S., Riwar, B., and Jessberger, R. (1999). Cloning and characterization of mammalian SMC1 and SMC3 genes and proteins, components of the DNA recombination complexes RC-1. *Gene* 228(1-2)**,** 1-12. doi: 10.1016/s0378-1119(99)00021-9.

Sun, X., Perlick, H.A., Dietz, H.C., and Maquat, L.E. (1998). A mutated human homologue to yeast Upf1 protein has a dominant-negative effect on the decay of nonsense-containing mRNAs in mammalian cells. *Proc Natl Acad Sci U S A* 95(17)**,** 10009-10014.

Sweet, T., Yen, W., Khalili, K., and Amini, S. (2008). Evidence for involvement of NFBP in processing of ribosomal RNA. *J Cell Physiol* 214(2)**,** 381-388. doi: 10.1002/jcp.21204.

Takata, H., Nishijima, H., Maeshima, K., and Shibahara, K. (2012). The integrator complex is required for integrity of Cajal bodies. *J Cell Sci* 125(Pt 1)**,** 166-175. doi: 10.1242/jcs.090837.

Takemura, M., Yoshida, S., Akiyama, T., Kitagawa, M., and Yamada, Y. (2006). Role of the second-largest subunit of DNA polymerase alpha in the interaction between the catalytic subunit and hyperphosphorylated retinoblastoma protein in late S phase. *Biochim Biophys Acta* 1764(9)**,** 1447-1453. doi: 10.1016/j.bbapap.2006.06.015.

Tan, F.E., and Elowitz, M.B. (2014). Brf1 posttranscriptionally regulates pluripotency and differentiation responses downstream of Erk MAP kinase. *Proc Natl Acad Sci U S A* 111(17)**,** E1740-1748. doi: 10.1073/pnas.1320873111.

Turner, A.J., Knox, A.A., Prieto, J.L., McStay, B., and Watkins, N.J. (2009). A novel small-subunit processome assembly intermediate that contains the U3 snoRNP, nucleolin, RRP5, and DBP4. *Mol Cell Biol* 29(11)**,** 3007-3017. doi: 10.1128/MCB.00029-09.

Urban, M., Joubert, N., Purse, B.W., Hocek, M., and Kuchta, R.D. (2010). Mechanisms by which human DNA primase chooses to polymerize a nucleoside triphosphate. *Biochemistry* 49(4)**,** 727-735. doi: 10.1021/bi9019516.

Urlaub, H., Raker, V.A., Kostka, S., and Luhrmann, R. (2001). Sm protein-Sm site RNA interactions within the inner ring of the spliceosomal snRNP core structure. *EMBO J* 20(1-2)**,** 187-196. doi: 10.1093/emboj/20.1.187.

Urquhart, A.J., Gatei, M., Richard, D.J., and Khanna, K.K. (2011). ATM mediated phosphorylation of CHD4 contributes to genome maintenance. *Genome Integr* 2(1)**,** 1. doi: 10.1186/2041-9414-2-1.

Valdez, B.C., Perlaky, L., Saijo, Y., Henning, D., Zhu, C., Busch, R.K., et al. (1992). A region of antisense RNA from human p120 cDNA with high homology to mouse p120 cDNA inhibits NIH 3T3 proliferation. *Cancer Res* 52(20)**,** 5681-5686.

van Dijk, E.L., Schilders, G., and Pruijn, G.J. (2007). Human cell growth requires a functional cytoplasmic exosome, which is involved in various mRNA decay pathways. *RNA* 13(7)**,** 1027-1035. doi: 10.1261/rna.575107.

van Hille, B., Richener, H., Evans, D.B., Green, J.R., and Bilbe, G. (1993). Identification of two subunit A isoforms of the vacuolar H(+)-ATPase in human osteoclastoma. *J Biol Chem* 268(10)**,** 7075-7080.

Wada, M., Miyazawa, H., Wang, R.S., Mizuno, T., Sato, A., Asashima, M., et al. (2002). The second largest subunit of mouse DNA polymerase epsilon, DPE2, interacts with SAP18 and recruits the Sin3 co-repressor protein to DNA. *J Biochem* 131(3)**,** 307-311.

Waga, S., and Stillman, B. (1998). The DNA replication fork in eukaryotic cells. *Annu Rev Biochem* 67**,** 721-751. doi: 10.1146/annurev.biochem.67.1.721.

Wang, P., and Heitman, J. (2005). The cyclophilins. *Genome Biol* 6(7)**,** 226. doi: 10.1186/gb-2005-6-7-226.

Wang, Z., and Roeder, R.G. (1995). Structure and function of a human transcription factor TFIIIB subunit that is evolutionarily conserved and contains both TFIIB- and high-mobility-group protein 2-related domains. *Proc Natl Acad Sci U S A* 92(15)**,** 7026-7030. doi: 10.1073/pnas.92.15.7026.

Wasmuth, J.J., and Carlock, L.R. (1986). Chromosomal localization of human gene for histidyl-tRNA synthetase: clustering of genes encoding aminoacyl-tRNA synthetases on human chromosome 5. *Somat Cell Mol Genet* 12(5)**,** 513-517.

Watanabe, Y., Miyasaka, K.Y., Kubo, A., Kida, Y.S., Nakagawa, O., Hirate, Y., et al. (2017). Notch and Hippo signaling converge on Strawberry Notch 1 (Sbno1) to synergistically activate Cdx2 during specification of the trophectoderm. *Sci Rep* 7**,** 46135. doi: 10.1038/srep46135.

Watrin, E., and Peters, J.M. (2009). The cohesin complex is required for the DNA damage-induced G2/M checkpoint in mammalian cells. *EMBO J* 28(17)**,** 2625-2635. doi: 10.1038/emboj.2009.202.

Wei, W., Dorjsuren, D., Lin, Y., Qin, W., Nomura, T., Hayashi, N., et al. (2001). Direct interaction between the subunit RAP30 of transcription factor IIF (TFIIF) and RNA polymerase subunit 5, which contributes to the association between TFIIF and RNA polymerase II. *J Biol Chem* 276(15)**,** 12266-12273. doi: 10.1074/jbc.M009634200.

Wei, W., Gu, J.X., Zhu, C.Q., Sun, F.Y., Dorjsuren, D., Lin, Y., et al. (2003). Interaction with general transcription factor IIF (TFIIF) is required for the suppression of activated transcription by RPB5-mediating protein (RMP). *Cell Res* 13(2)**,** 111-120. doi: 10.1038/sj.cr.7290155.

Xue, Y., Wong, J., Moreno, G.T., Young, M.K., Cote, J., and Wang, W. (1998). NURD, a novel complex with both ATP-dependent chromatin-remodeling and histone deacetylase activities. *Mol Cell* 2(6)**,** 851-861.

Yaffe, M.B., Farr, G.W., Miklos, D., Horwich, A.L., Sternlicht, M.L., and Sternlicht, H. (1992). TCP1 complex is a molecular chaperone in tubulin biogenesis. *Nature* 358(6383)**,** 245-248. doi: 10.1038/358245a0.

Yam, A.Y., Xia, Y., Lin, H.T., Burlingame, A., Gerstein, M., and Frydman, J. (2008). Defining the TRiC/CCT interactome links chaperonin function to stabilization of newly made proteins with complex topologies. *Nat Struct Mol Biol* 15(12)**,** 1255-1262. doi: 10.1038/nsmb.1515.

Yang, W., Itoh, F., Ohya, H., Kishimoto, F., Tanaka, A., Nakano, N., et al. (2011). Interference of E2-2-mediated effect in endothelial cells by FAM96B through its limited expression of E2-2. *Cancer Sci* 102(10)**,** 1808-1814. doi: 10.1111/j.1349-7006.2011.02022.x.

Yeh, J.I., Levine, A.S., Du, S., Chinte, U., Ghodke, H., Wang, H., et al. (2012). Damaged DNA induced UV-damaged DNA-binding protein (UV-DDB) dimerization and its roles in chromatinized DNA repair. *Proc Natl Acad Sci U S A* 109(41)**,** E2737-2746. doi: 10.1073/pnas.1110067109.

Yeh, P.C., Yeh, C.C., Chen, Y.C., and Juang, Y.L. (2012). RED, a spindle pole-associated protein, is required for kinetochore localization of MAD1, mitotic progression, and activation of the spindle assembly checkpoint. *J Biol Chem* 287(15)**,** 11704-11716. doi: 10.1074/jbc.M111.299131.

Yeh, T.Y., Kowalska, A.K., Scipioni, B.R., Cheong, F.K., Zheng, M., Derewenda, U., et al. (2013). Dynactin helps target Polo-like kinase 1 to kinetochores via its left-handed beta-helical p27 subunit. *EMBO J* 32(7)**,** 1023-1035. doi: 10.1038/emboj.2013.30.

Yoo, H., Son, D., Jang, Y.J., and Hong, K. (2016). Indispensable role for mouse ELP3 in embryonic stem cell maintenance and early development. *Biochem Biophys Res Commun* 478(2)**,** 631-636. doi: 10.1016/j.bbrc.2016.07.120.

Yorikawa, C., Shibata, H., Waguri, S., Hatta, K., Horii, M., Katoh, K., et al. (2005). Human CHMP6, a myristoylated ESCRT-III protein, interacts directly with an ESCRT-II component EAP20 and regulates endosomal cargo sorting. *Biochem J* 387(Pt 1)**,** 17-26. doi: 10.1042/BJ20041227.

Zeng, F., Hua, Y., Liu, X., Liu, S., Lao, K., Zhang, Z., et al. (2018). Gpn2 and Rba50 Directly Participate in the Assembly of the Rpb3 Subcomplex in the Biogenesis of RNA Polymerase II. *Mol Cell Biol* 38(13). doi: 10.1128/MCB.00091-18.

Zeng, L., Zhou, Z., Xu, J., Zhao, W., Wang, W., Huang, Y., et al. (2001). Molecular cloning, structure and expression of a novel nuclear RNA-binding cyclophilin-like gene (PPIL4) from human fetal brain. *Cytogenet Cell Genet* 95(1-2)**,** 43-47. doi: 10.1159/000057015.

Zhan, R., Yamamoto, M., Ueki, T., Yoshioka, N., Tanaka, K., Morisaki, H., et al. (2013). A DEAD-box RNA helicase Ddx54 protein in oligodendrocytes is indispensable for myelination in the central nervous system. *J Neurosci Res* 91(3)**,** 335-348. doi: 10.1002/jnr.23162.

Zhang, Y., LeRoy, G., Seelig, H.P., Lane, W.S., and Reinberg, D. (1998). The dermatomyositis-specific autoantigen Mi2 is a component of a complex containing histone deacetylase and nucleosome remodeling activities. *Cell* 95(2)**,** 279-289. doi: 10.1016/s0092-8674(00)81758-4.

Zhao, H., Han, Z., Liu, X., Gu, J., Tang, F., Wei, G., et al. (2017). The chromatin remodeler Chd4 maintains embryonic stem cell identity by controlling pluripotency- and differentiation-associated genes. *J Biol Chem* 292(20)**,** 8507-8519. doi: 10.1074/jbc.M116.770248.

Zhou, C., Cunningham, L., Marcus, A.I., Li, Y., and Kahn, R.A. (2006). Arl2 and Arl3 regulate different microtubule-dependent processes. *Mol Biol Cell* 17(5)**,** 2476-2487. doi: 10.1091/mbc.e05-10-0929.
