# Supplementary material for: An Integrated Model of Minor Intron Emergence and Conservation
Source: Front Genet. 2019 Nov 13;10:1113. doi: 10.3389/fgene.2019.01113 (PMC6865273; doi:10.3389/fgene.2019.01113)
Supplement: Supplementary file 4 [file DataSheet_1.docx]

**Supplementary Methods**

**Analysis of gene enrichment in the essentialome**

The Broad Institute essentiality data for the 342 cancer cell lines was used to identify genes essential for the survival of each cancer cell line, based on the thresholding described by Meyers et al., 2017. In accordance with recommendations from the Broad Institute, we removed the PK59_PANCREAS cell line from our analysis, since it had failed subsequent quality controls (Cancer, 2017). Classification of the remaining 341 cell lines by cancer origin/type was performed based on the cell line data provided in Supplementary Table 1 by Meyers et al., 2017. Statistical significance for MIG enrichment was calculated by Fisher’s exact test.

**Analysis of cell cycle RNAseq data**

RNAseq data from Singh et al. (2013) was processed through our previously reported bioinformatics pipeline (Baumgartner et al., 2018). A gene was deemed expressed if it showed TPM≥1 in at least one stage of cell cycle. Differential expression was called if a gene showed ≥2-fold change (*P*≤0.01) expression difference between two stages of cell cycle.
